# Methylfolate Trap Promotes Bacterial Thymineless Death by Sulfa Drugs

**DOI:** 10.1371/journal.ppat.1005949

**Published:** 2016-10-19

**Authors:** Marissa B. Guzzo, Hoa T. Nguyen, Thanh H. Pham, Monika Wyszczelska-Rokiel, Hieronim Jakubowski, Kerstin A. Wolff, Sam Ogwang, Joseph L. Timpona, Soumya Gogula, Michael R. Jacobs, Markus Ruetz, Bernhard Kräutler, Donald W. Jacobsen, Guo-Fang Zhang, Liem Nguyen

**Affiliations:** 1 Department of Molecular Biology and Microbiology, Case Western Reserve University School of Medicine, Cleveland, Ohio, United States of America; 2 Department of Pathology, Case Western Reserve University School of Medicine, Cleveland, Ohio, United States of America; 3 Department of Nutrition, Case Western Reserve University School of Medicine, Cleveland, Ohio, United States of America; 4 Department of Microbiology, Biochemistry and Molecular Genetics, Rutgers University, New Jersey Medical School, Newark, New Jersey, United States of America; 5 Department of Environmental Chemistry, University of Lodz, Lodz, Poland; 6 Institute of Bioorganic Chemistry, Polish Academy of Sciences, Poznań, Poland; 7 Department of Biochemistry and Biotechnology, Life Sciences University, Poznań, Poland; 8 Institute of Organic Chemistry and Center of Molecular Biosciences, University of Innsbruck, Innsbruck, Austria; 9 Department of Cellular and Molecular Medicine, Lerner Research Institute, Cleveland Clinic, Cleveland, Ohio, United States of America; National Institutes of Health, UNITED STATES

## Abstract

The methylfolate trap, a metabolic blockage associated with anemia, neural tube defects, Alzheimer’s dementia, cardiovascular diseases, and cancer, was discovered in the 1960s, linking the metabolism of folate, vitamin B_12_, methionine and homocysteine. However, the existence or physiological significance of this phenomenon has been unknown in bacteria, which synthesize folate *de novo*. Here we identify the methylfolate trap as a novel determinant of the bacterial intrinsic death by sulfonamides, antibiotics that block *de novo* folate synthesis. Genetic mutagenesis, chemical complementation, and metabolomic profiling revealed trap-mediated metabolic imbalances, which induced thymineless death, a phenomenon in which rapidly growing cells succumb to thymine starvation. Restriction of B_12_ bioavailability, required for preventing trap formation, using an “antivitamin B_12_” molecule, sensitized intracellular bacteria to sulfonamides. Since boosting the bactericidal activity of sulfonamides through methylfolate trap induction can be achieved in Gram-negative bacteria and mycobacteria, it represents a novel strategy to render these pathogens more susceptible to existing sulfonamides.

## Author Summary

Sulfonamides were the first agents to successfully treat bacterial infections, but their use later declined due to the emergence of resistant organisms. Restoration of these drugs may be achieved through inactivation of molecular mechanisms responsible for resistance. A chemo-genomic screen first identified 50 chromosomal loci representing the whole-genome antifolate resistance determinants in *Mycobacterium smegmatis*. Interestingly, many determinants resembled components of the methylfolate trap, a metabolic blockage exclusively described in mammalian cells. Targeted mutagenesis, genetic and chemical complementation, followed by chemical analyses established the methylfolate trap as a novel mechanism of sulfonamide sensitivity, ubiquitously present in mycobacteria and Gram-negative bacterial pathogens. Furthermore, metabolomic analyses revealed trap-mediated interruptions in folate and related metabolic pathways. These metabolic imbalances induced thymineless death, which was reversible with exogenous thymine supplementation. Chemical restriction of vitamin B_12_, an important molecule required for prevention of the methylfolate trap, sensitized intracellular bacteria to sulfonamides. Thus, pharmaceutical promotion of the methylfolate trap represents a novel folate antagonistic strategy to render pathogenic bacteria more susceptible to available, clinically approved sulfonamides.

## Introduction

**Fig 1:**
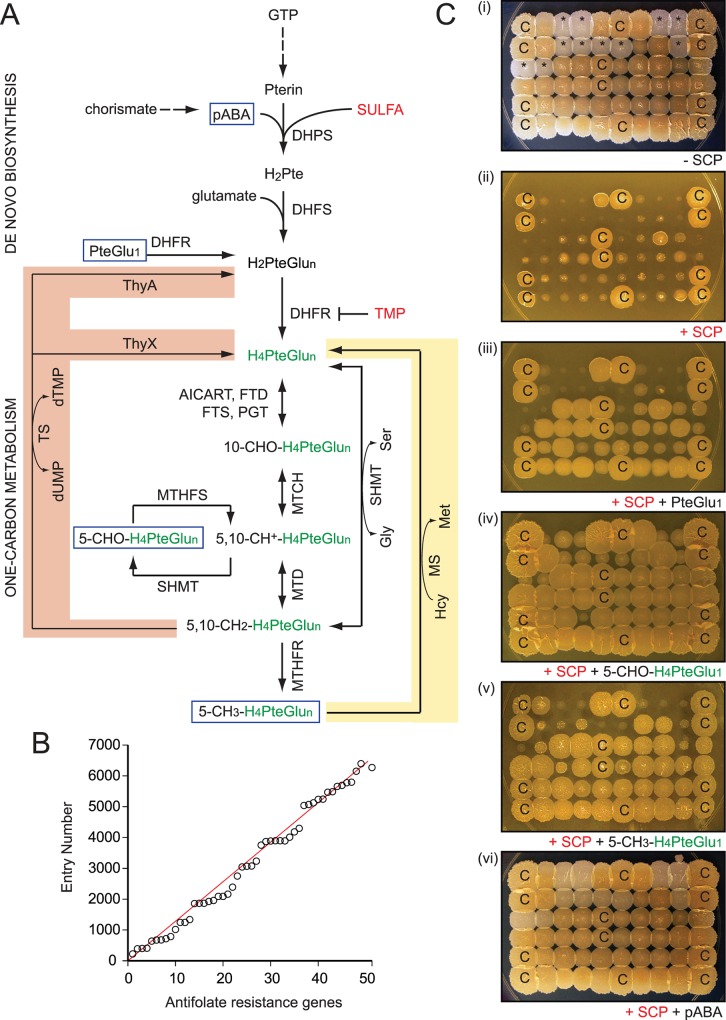
Chemo-genomic characterization of antifolate resistance determinants in *M*. *smegmatis*. (**A**) Simplified enzymatic conversions of folate derivatives in *de novo* biosynthesis and the one-carbon metabolic network in bacteria. Abbreviations: H_4_PteGlu_n_, tetrahydrofolate (green) serves as carrier for one-carbon groups. AICART, aminoimidazolecarboxamide ribonucleotide transferase; DHFS, dihydrofolate synthase; DHFR, dihydrofolate reductase; DHPS, dihydropteroate synthase; FTD, 10-formyltetrahydrofolate dehydrogenase; FTS, 10-formyltetrahydrofolate synthetase; Gly, glycine; GTP, guanosine triphosphate; H_2_PteGlu_n_, dihydrofolate; Hcy, homocysteine; Met, methionine; MS, methionine synthase; MTCH, methylenetetrahydrofolate cyclohydrolase; MTD, methylenetetrahydrofolate dehydrogenase; MTHFR, methylenetetrahydrofolate reductase; MTHFS, 5,10-methenyltetrahydrofolate synthetase; pABA, para-aminobenzoic acid; PGT, phosphoribosyl glycinamide transferase; Pte, pteroate; PteGlu_1_, folic acid; Ser, serine; SHMT, serine hydroxymethyltransferase; TS, thymidylate synthase. Two different types of TS have been described: ThyA and ThyX. While most organisms contain either ThyA or ThyX, some organisms including *M*. *tuberculosis* have both. Reactions directly involved in the methylfolate trap (MS) and thymineless death (TS) are highlighted in yellow and red, respectively. (**B**) Genome distributions of antifolate resistance determinants in *M*. *smegmatis*. Laboratory assigned catalog numbers (n = 1–50, S1 Table) were plotted against their corresponding locus tags (*msmeg_*No.). (**C**) A typical SULFA susceptibility and chemical complementation assay of *M*. *smegmatis* strains. A pool of antifolate sensitive mutants was replicated onto NE plates, in top-down order: (*i*) control, (*ii*) SCP, (*iii*) SCP plus PteGlu_1_, (*iv*) SCP plus 5-CHO-H_4_PteGlu_1_, (*v*) SCP plus 5-CH_3_-H_4_PteGlu_1_, and (*vi*) SCP plus pABA. SCP was used at 10.5 μg/ml while supplements were used at 0.3 mM final concentration. Colonies marked with “C” were from the parental strain mc^2^155, which was used as a control. Colonies marked with asterisks were from the “white” mutants.

Sulfonamides, or SULFA drugs, were the first chemical substances systematically used to treat and prevent bacterial infections [1, 2], but the use of these drugs gradually declined because of the emergence of resistant organisms [3]. To increase SULFAs’ potency and prevent further resistance, trimethoprim (TMP), which provides synergy, was later developed [4]. Combination regimens using TMP and SULFAs have effectively treated acute urinary tract infections, bacterial meningitis, *Pneumocystis jiroveci* pneumonia, and shigellosis, and are commonly used as prophylaxis against recurrent and drug resistant infections [3, 5, 6]. Unfortunately, TMP has been the only SULFA booster approved for clinical use, and resistance to both TMP and SULFAs has emerged [7]. In addition, the synergistic effect of TMP remains questionable in many bacteria, including *Mycobacterium tuberculosis* and *Pseudomonas aeruginosa* [8, 9]. To protect the efficacy of SULFAs and safely expand their clinical use [10], novel SULFA boosters are required. A recent strategy for developing antibiotic boosters is “resisting resistance” [11], in which inhibitors that suppress resistance mechanisms are used to sensitize host bacteria to antibiotics. Our laboratory recently suggested that targeting antifolate resistance may lead to the development of such adjunctive chemotherapies for SULFAs and TMP [12]. We found that disruption of 5,10-methenyltetrahydrofolate synthase (MTHFS), an enzyme responsible for the conversion of *N* ^5^-formyltetrahydrofolate (5-CHO-H_4_PteGlu_n_) to *N* ^5^,*N* ^10^-methenyltetrahydrofolate (5,10-CH^+^-H_4_PteGlu_n_) in the folate-dependent one-carbon metabolic network (Fig 1A), led to severe defects in cellular folate homeostasis thus weakening the intrinsic antifolate resistance in bacteria [12].

TMP and SULFAs are bacteriostatic in minimal media. However, they become more bactericidal in rich media, particularly when cellular levels of glycine, methionine and purines are high. In such conditions, the multifactorial deficiency caused by SULFAs is reduced to a single deficiency of thymine (Fig 1A, highlighted in red), and cells undergoing such “unbalanced growth” succumb to thymineless death [13–17]. Exogenous thymine supplementation reduces the bactericidal activity of SULFAs and TMP [14, 15]. Classified as folate antagonists, or antifolates, these drugs inhibit bacterial *de novo* folate biosynthesis (Fig 1A), which is absent in mammalian cells. While SULFAs target dihydropteroate synthase (DHPS), TMP inhibits dihydrofolate reductase (DHFR). Both of these enzymes are required for the formation of folate, a vitamin essential for cell growth across all kingdoms of life. The dominant form of folate in the cell is tetrahydrofolate (H_4_PteGlu_n_, with n indicating the number of glutamate moieties). This reduced folate molecule functions as a carrier of one-carbon units in multiple metabolic reactions that are required for the production of purines, thymidine, amino acids, and the recycling of homocysteine (Hcy), a non-protein amino acid harmful to long half-life proteins (Fig 1A) [18].

Antifolate-mediated folate deficiency affects the biosynthesis of nucleic acids and proteins, as well as other important cellular processes including methylation and homeostasis of Hcy [18]. In humans, defects in Hcy homeostasis, or hyperhomocysteinemia, are often associated with folate and vitamin B_12_ deficiencies observed in medical conditions such as anemia, neural tube defects, cardiovascular diseases, Alzheimer’s dementia, stroke, cancers, and others [18]. This interconnected metabolic syndrome has been explained by the “methylfolate trap” hypothesis that assigns its cause to defects in the multi-cycling reaction catalyzed by the B_12_-dependent methionine synthase (MetH, EC:2.1.1.13) (Fig 1A, highlighted in yellow) [19–21]. This reaction depends on three components: (*i*) *N* ^5^-methyltetrahydrofolate (5-CH_3_-H_4_PteGlu_n_), a methyl donor, (*ii*) B_12_, the intermediate carrier for the methyl group, and (*iii*) the catalytic activity provided by MetH. Besides the methylation of Hcy to form methionine, this reaction recycles 5-CH_3_-H_4_PteGlu_n_ back to free H_4_PteGlu_n_ which can be further converted to other folate forms (Fig 1A) [20, 21]. This reaction can be compromised by B_12_ deficiency and/or mutations affecting MetH enzymatic activity. Consequently, the cellular pool of H_4_PteGlu_n_ is trapped in the methylated form (5-CH_3_-H_4_PteGlu_n_), thus interrupting the normal flow of the one-carbon metabolic network (Fig 1A) [21–24]. 5-CH_3_-H_4_PteGlu_n_ is generated from *N* ^5^,*N* ^10^-methylenetetrahydrofolate (5,10-CH_2_-H_4_PteGlu_n_) in an upstream reaction catalyzed by methylenetetrahydrofolate reductase (MTHFR), which is irreversible *in vivo* [25] and suppressed by *S*-adenosylmethionine (SAM) [26]. Since SAM is produced from methionine, inhibition of MetH activity leads to reduced SAM levels, thus resulting in derepression of MTHFR, further accelerating the accumulation of 5-CH_3_-H_4_PteGlu_n_ and Hcy [26]. Attempts to delete *metH* in mice were unsuccessful as homozygous knockout embryos all died following implantation [27]. Although it has been studied in humans, and *ex vivo* in mammalian cells, the existence or physiological significance of the methylfolate trap in bacteria has never been documented.

Here we report the identification of the methylfolate trap as a novel determinant of SULFA resistance in bacteria. Upon its formation in response to SULFAs, the methylfolate trap causes impaired homeostasis of folate and related metabolites, including a progressive accumulation of Hcy-thiolactone that is known to be cytotoxic. More importantly, cells undergoing the methylfolate trap are also unable to deplete glycine and nucleotides, and suffer thymineless death induced by SULFAs. This metabolic blockage renders pathogenic bacteria, including *M*. *tuberculosis*, *P*. *aeruginosa*, *Escherichia coli* and *Salmonella typhimurium* more susceptible to existing SULFAs both *in vitro* and in host macrophages. Furthermore, chemical induction of the methylfolate trap, as shown in our experiments, represents a viable method for boosting the antimicrobial activity of available, clinically approved SULFAs against bacterial pathogens.

## Results

### Genome-wide characterization of antifolate resistance in *M*. *smegmatis*

A screen of 13,500 *Himar1*-transposon *M*. *smegmatis* mutants (details can be found in Supplemental S1 Text) identified a collection of strains that displayed normal growth in the absence of antifolates but suffered defects in antifolate resistance. After 2 rounds of drug susceptibility tests, the disrupted genes were mapped using nested PCRs, followed by sequencing. Of the 50 chromosomal loci identified as being responsible for the intrinsic antifolate resistance of *M*. *smegmatis* (S1 Table), 31 genes (62%) encoded enzymatic activities, 14 of which (28%) were predicted to be involved in folate metabolism or related pathways. The identification of many genes whose functions are related to folate metabolism indicated that the screen was successful. Overall, the resistance determinants were evenly distributed throughout the *M*. *smegmatis* genome with some relatively discrete regions and gaps (Fig 1B).

Besides many genes encoding homologs of enzymes of the one-carbon metabolic network and related metabolism of amino acids or nucleotides (*fmt*, *dcd*, *gabD*, *cobIJ*, *metH*, *glyA*, *ygfA*, and *ygfZ*), genetic mapping revealed *Himar1* insertions in genes that encode proteins previously known to provide non-specific antibiotic resistance (*pknG*, *mshB*, *cspB*, *fbpA*, and *treS*) [28–33] (S1 Table). In addition, insertions were mapped to chromosomal loci potentially affecting regulatory or signaling processes (*mprA*, *sigB*, *sigE*, *pknG*, *pafA*, *pup*, *pcrB*, and *pcrA*), transsulfuration (*cysH* and *mshB*), transport (*mmpL* and *pstC*), and other cellular activities (S1 Table).

Mutants were further profiled using chemical complementation. Para-aminobenzoic acid (pABA) or a folate derivative (Fig 1A, blue rectangles, & Fig 1C) was added exogenously to support growth in the presence of SULFAs or TMP, which inhibit *de novo* folate synthesis. These analyses provided useful geno-chemo-phenotypic information to each individual antifolate resistance determinant (S1 Table).

### Methylfolate trap as a SULFA resistance determinant in *M*. *smegmatis*

**Fig 2:**
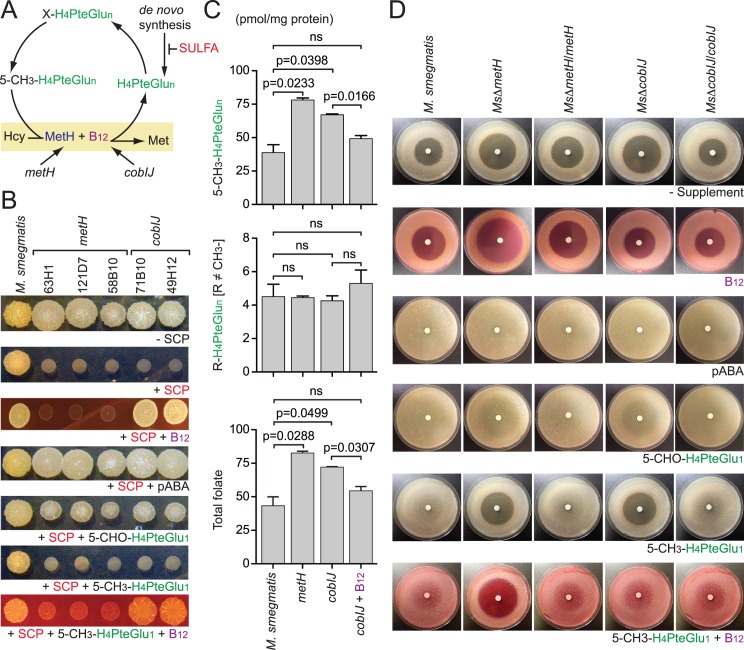
Methylfolate trap in *Mycobacterium smegmatis*. (**A**) A model depicting the chemical conversions and factors involved in the methylfolate trap-mediated SULFA sensitivity. The CH_3_- group in 5-CH_3_-H_4_PteGlu_n_ is first transferred to the B_12_ cofactor, which further transfers it to homocysteine (Hcy) to make methionine (Met). The MetH reaction thereby recycles 5-CH_3_-H_4_PteGlu_n_ back to free H_4_PteGlu_n_ which continues the flow of the one-carbon network. (**B**) Chemical complementation of *M*. *smegmatis* “white” mutants mapped to *metH* or *cobIJ*. The strains exhibited increased SULFA susceptibility and impaired 5-CH_3_-H_4_PteGlu_1_ utilization. Approximately 5x10^3^ cells were spotted onto NE medium added with 10.5 μg/ml SCP with or without exogenous supplements. Unlike wild type and other mutants, these mutants were unable to use 5-CH_3_-H_4_PteGlu_1_ to antagonize SCP. Exogenous B_12_ restored 5-CH_3_-H_4_PteGlu_1_ utilization and SCP resistance to *cobIJ* but not *metH* mutants. (**C**) Effect of *metH* and *cobIJ* on the folate pool in *M*. *smegmatis*. Growing cultures of *M*. *smegmatis* strains were treated with 285 μg/ml SCP for 30 min followed by folate extraction and LC-MS/MS analysis. Data shows the combined levels of all 5-CH_3_-H_4_PteGlu_n_ species (top), all non-methyl folate species (middle), and the total folate (bottom). Bars represent means of biological triplicates with standard deviations. P values are shown above the bars and were calculated using unpaired Student’s t-test; ns, no significant difference between the indicated strains. (**D**) Targeted mutagenesis confirms the roles of *metH* and *cobIJ* in methylfolate trap-induced SULFA sensitivity and 5-CH_3_-H_4_PteGlu_1_ utilization in *M*. *smegmatis*. Paper discs were embedded with 0.5 mg SCP and placed at the center of the medium surface, seeded with bacterial strains. Exogenous B_12_ and 5-CH_3_-H_4_PteGlu_n_ were used at 0.3 and 1 mM, respectively. Genetic complementation was achieved by *in trans* expression of *metH* or *cobIJ*. B_12_ alone restored wild type SULFA resistance level to *Ms*Δ*cobIJ*, whereas the combination of 5-CH_3_-H_4_PteGlu_1_ and B_12_ completely abolished SULFA resistance to all strains but *Ms*Δ*metH*.

Chemical complementation identified a group of SULFA-sensitive, “white” mutants that lost the yellow pigment typically displayed by *M*. *smegmatis* (Fig 1C, marked with asterisks). The mutants were unable to use exogenous 5-CH_3_-H_4_PteGlu_1_ to antagonize SULFAs (Fig 1C, panel (*v*)). Genetic mapping showed that four mutants in this subgroup had *Himar1* insertions at three different TA dinucleotides within the same gene, *msmeg_4185* (2xTA^499-500^, 1xTA^2881-2882^, and 1xTA^3091-3092^, S1 Fig), which encodes a homolog of B_12_-dependent methionine synthase. Two other mutants had insertions at TA^112-113^ of *msmeg_3873*, which encodes an enzyme (CobIJ) that catalyzes two methylation steps, precorrin-2 C20 methyltransferase [CobI, EC:2.1.1.130] and precorrin-3B C17-methyltransferase [CobJ, EC:2.1.1.131], of the B_12_ (cobalamin) biosynthetic pathway. Interestingly, the function of these 5-CH_3_-H_4_PteGlu_n_-related genes were reminiscent of factors involved in the methylfolate trap, a metabolic disorder thus far only described in mammalian cells (Fig 2A). Whereas the *metH*-encoded enzyme catalyzes the reaction, *cobIJ* is required for the *de novo* biosynthesis of B_12_, the cofactor required for MetH activity.

Exogenous B_12_ restored both SULFA resistance and 5-CH_3_-H_4_PteGlu_1_ utilization to *cobIJ*, but failed to restore the *metH* strains (Fig 2B), resembling the “pseudo-folate deficiency” phenomenon previously observed in anemia patients (described in the Discussion) [19]. To detect the methylfolate trap at a metabolic level, *M*. *smegmatis* strains growing in a liquid medium were challenged with sulfachloropyridazine (SCP) for half an hour to starve the cells from newly synthesized folate. Cultures were immediately harvested and total folate was extracted in subdued light. Samples added with internal standards were analyzed by LC-MS/MS as previously described [12]. Both *metH* and *cobIJ* exhibited 5-CH_3_-H_4_PteGlu_n_ accumulation compared to wild type *M*. *smegmatis* (Fig 2C). Exogenous B_12_ significantly reduced 5-CH_3_-H_4_PteGlu_n_ accumulation in the *cobIJ* mutant, though not to the level of wild type (Fig 2C). This B_12_-responsive alteration in the cellular folate pool of *cobIJ* explained its pseudo-folate deficiency-like behavior in susceptibility tests (Fig 2B). In the *cobIJ* mutant, the *metH* gene remained intact but its encoded protein did not have enough B_12_, due to the *Himar1* insertion into *cobIJ* disrupting *de novo* B_12_ biosynthesis, to activate its methionine synthase activity. When B_12_ was exogenously supplemented, the cofactor activated MetH activity, thus bypassing the B_12_ synthetic defect allowing for the release of the methylfolate trap.

To confirm that MetH and CobIJ contribute to the intrinsic SULFA resistance, and 5-CH_3_-H_4_PteGlu_n_ metabolism, the encoding genes, *msmeg_4185* and *msmeg_3873*, respectively, were individually deleted by homologous recombination [34]. Similar to the transposon mutants, the targeted null mutants, *Ms*Δ*metH* and *Ms*Δ*cobIJ*, displayed increased SULFA susceptibility and impaired utilization of exogenous 5-CH_3_-H_4_PteGlu_1_ whereas *in trans* expression of *metH* and *cobIJ*, respectively, restored both phenotypes (Table 1, Fig 2D). Exogenous B_12_ restored both SULFA resistance and 5-CH_3_-H_4_PteGlu_1_ utilization to *Ms*Δ*cobIJ*, but failed to do so for *Ms*Δ*metH*. Although the mutants were hypersusceptible to all SULFAs tested (S2 Fig), resistance to non-antifolate antibiotics remained unaffected (S3 Fig). While *M*. *smegmatis* encodes a B_12_-independent methionine synthase (MetE, EC: 2.1.1.14) [35], deletion of *metE* did not affect SULFA sensitivity (S4 Fig and S5 Fig). These observations confirmed that MetH is essential for normal 5-CH_3_-H_4_PteGlu_n_ metabolism, which is required for the intrinsic SULFA resistance in *M*. *smegmatis*.

**Table 1:** Susceptibility of bacterial strains to antifolates.

MIC (μg/ml) ^a^	SMZ	TMP	SMZ/TMP ^b^
*P*. *aeruginosa*	800	250	712.5/37.5
*Pa*.*metH*	80	30	47.5/2.5
*Pa*.*btuB*	70	30	38/2
*Pa*.*cobI*	135	30	57/3
*Pa*.*cobJ*	135	30	57/3
*Pa*.*cobH*	135	30	57/3
*E*. *coli*	255	0.375	2.85/0.15
*Ec*Δ*metH*	30	0.325	2.85/0.15
*Ec*Δ*btuB*	30	0.325	2.85/0.15
*Ec*Δ*btuCED*	255	0.375	2.85/0.15
*Ec*Δ*btuCED*Δ*btuB*	30	0.375	2.85/0.15
*S*. *typhimurium*	385	0.1	1.425/0.075
*St*.*metE*	385	0.1	1.425/0.075
*St*.*metE metH*	55	0.1	1.425/0.075
*St*.*metE btuB*	35	0.1	1.425/0.075
*St*.*metE cobI cobJ*	385	0.1	1.425/0.075
*St*.*metE cobI cobJ btuB*	35	0.1	1.425/0.075
*St*.*metE cobI*	385	0.1	1.425/0.075
*M*. *smegmatis*	1	ND ^e^	ND
*Ms*Δ*metH*	0.125	ND	ND
*Ms*Δ*metH*/*metH*	2	ND	ND
*Ms*Δ*cobIJ*	0.125	ND	ND
*Ms*Δ*cobIJ*/*cobIJ*	1	ND	ND
*Ms*.*metH*	0.125	ND	ND
*Ms*.*cobIJ*	0.125	ND	ND
*M*. *tuberculosis* H37Rv ^c^	25	ND	ND
RvΔ*metH*	0.1	ND	ND
RvΔ*metH* ^d^	1	ND	ND
RvΔ*metH*/*metH*	25	ND	ND
RvΔ*cobIJ*	25	ND	ND

Luria Broth was used for Gram-negative bacteria; 7H9-S and Dubos were used for *M*. *tuberculosis* and *M*. *smegmatis*, respectively.

^a^ Abbreviations: MIC, minimal inhibitory concentration, defined as the lowest concentration of an antibiotic that inhibits the visible growth of bacteria; *Pa*, *Pseudomonas aeruginosa*; *Ec*, *Escherichia coli*; *St*, *Salmonella typhimurium*; *Ms*, *Mycobacterium smegmatis*; *Mtb*, *Mycobacterium tuberculosis*; SMZ, sulfamethoxazole. TMP, trimethoprim.

^b^ SMZ/TMP used at ratio 19/1.

^c^ MIC tests for *M*. *tuberculosis* were performed in the presence of 0.3 mM B_12_.

^d^ Methionine was added at 1 mM concentration.

^e^ Not determined.

### Methylfolate trap in *M*. *tuberculosis*

**Fig 3:**
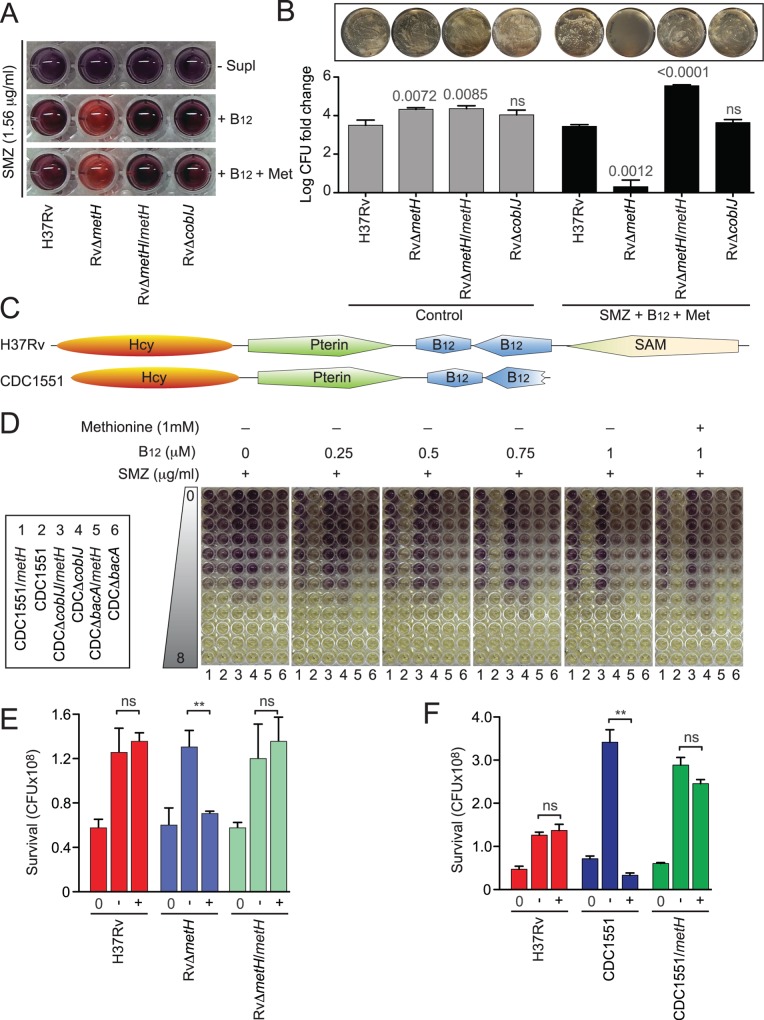
Methylfolate trap in *Mycobacterium tuberculosis*. (**A**) SULFA sensitivity of H37Rv-derived strains in 7H9-S medium, in the absence or presence of exogenous B_12_ and/or methionine (Met), was analyzed using the MTT method. Cultures grown to an OD_600_ of 2 were washed and diluted in 7H9-S. Wells were inoculated with 10^4^ cells in the presence of 1.56 μg/ml SMZ supplemented with 0.3 mM B_12_ alone and in combination with 1 mM methionine. Plates were incubated for 7 days at 37°C. MTT solution prepared in 1X PBS, pH 6.8, was added to each well and incubated for 24 hours. The reaction was stopped by adding SDS-DMF solution followed by incubation at 37°C for an additional 24 hours. Purple formazan indicates living cells. (**B**) H37Rv-derived strains were grown to OD_600_ of 1 and 5 μl cultures were spotted onto 7H10-OADC or the same medium supplemented with 5.7 μg/ml SCP, 0.5 mM B_12_, and 1 mM methionine. Plates were incubated at 37°C for 4 weeks. The spotted cell suspension for each strain under both conditions was collected and suspended in 7H9-OADC. Suspensions underwent 10-fold serial dilutions from which 100 μl aliquots were plated onto 7H10-OADC in triplicate. After 4 weeks of incubation at 37°C, viability was determined by counting colony forming unit (c.f.u.) and normalized to the c.f.u. values of the input inoculum. The y-axis represents c.f.u. fold-change on a log10 scale. Bars represent standard deviations from experimental triplicates. P values are shown above the bars and were calculated using unpaired Student’s t-test; ns, no significant difference compared to corresponding H37Rv sample in same condition. Representative 10^−6^ dilution plates provide a visual comparison between strains in viability (top). (**C**) Domain alignment of MetH proteins from H37Rv and CDC1551 using PROSITE (http://prosite.expasy.org). Domains are labeled as the cofactors to which they bind. (**D**) SULFA sensitivity of CDC1551-derived strains in Dubos medium in the absence or presence of B_12_ and methionine was analyzed using the MTT method. Cultures growing at an OD_600_ of 2 were washed and diluted in Dubos medium. Wells containing two-fold increasing SMZ concentrations (0–8 μg/ml) were inoculated with 10^4^ cells of each strain, as indicated in the box on the left. Test plates, supplemented with varying concentrations of B_12_ (0.25–1 μM), without or with 1 mM methionine, were incubated for 7 days at 37°C. MTT solution was added to each well and incubated for 24 hours. The reaction was stopped by adding SDS-DMF solution followed by incubation at 37°C for an additional 24 hours. Purple formazan indicates living cells. (**E**) Survival of H37Rv (Red), its derived *metH* mutant (RvΔ*metH*, Blue) and the complemented strain (RvΔ*metH*/*metH*, Green) in macrophages, non-treated or treated with 40 μg/ml SMZ. Presented data are the c.f.u. values of internalized bacteria at 0 h (0) and after 72 h chase without (-) or with (+) 40 μg/ml SMZ. Shown are means of biological triplicates with standard deviations. ** p<0.01; ns, no significant difference compared to corresponding H37Rv sample. The data presented is the representative of four independent experiments. (**F**) Survival of H37Rv (Red), CDC1551 (Blue), and the CDC1551 strain *in trans* expressing the intact *metH* gene from H37Rv (CDC1551/*metH*, Green) in macrophages, non-treated or treated with 40 μg/ml SMZ. Presented data are the c.f.u. values of internalized bacteria at 0 h (0) and after 72 h chase without (-) or with (+) 40 μg/ml SMZ. Shown are means of biological triplicates with standard deviations. ** p<0.01; ns, no significant difference compared to H37Rv.

Mutants lacking *metH* or *cobIJ* genes were first constructed from the *M*. *tuberculosis* laboratory strain H37Rv (see S1 Text). Sensitivity tests using the MTT method were performed with two minimal media, 7H9-S or Dubos, in the absence or presence of exogenous B_12_ (tested range: 1 μM—0.3 mM). In the absence of B_12_, SULFA susceptibility of the H37Rv-derived strains were similar. However, with B_12_ supplementation, significant differences in SULFA resistance among strains were observed (Table 1, Fig 3A). While RvΔ*metH* displayed high susceptibility to sulfamethoxazole (SMZ), the sensitivity level of RvΔ*cobIJ* was unchanged compared to wild type (Table 1, Fig 3A). *In trans* expression of *metH* completely restored wild type SULFA resistance to RvΔ*metH* (Table 1, Fig 3A). These results indicated that the methylfolate trap was able to sensitize *M*. *tuberculosis* H37Rv to SULFA drugs. Such trap formation, however, requires the absence of methionine synthase activities.

In agreement with previous studies [36, 37], our data suggested that H37Rv is unable to synthesize B_12_ *de novo*, and that this organism relies on its uptake system for obtaining B_12_ from the environment. In the complete absence of B_12_, H37Rv employed the B_12_-independent methionine synthase MetE to prevent the methylfolate trap. When B_12_ was added exogenously, MetE activity was inhibited, making RvΔ*metH* completely null of methionine synthases. In such a condition, the methylfolate trap was formed sensitizing RvΔ*metH* to SULFA drugs. It is important to note that exogenous supplementation of methionine only partially enhanced SMZ resistance of RvΔ*metH* (Table 1), indicating that the lack of methionine due to defective methionine synthases [37, 38] is not the sole contributor to the enhanced SULFA susceptibility. To further characterize the methionine-unrelated methylfolate trap-mediated SULFA sensitivity, survival of the *M*. *tuberculosis* strains treated with SMZ, B_12_, and methionine were assayed by serial dilution and colony forming unit (c.f.u.) counting. With similar inputs, the survival of RvΔ*metH* was 3 log10 lower than that of wild type *M*. *tuberculosis* H37Rv and the RvΔ*cobIJ* mutant (Fig 3B). This result not only confirmed our observation from the growth inhibition assays (Table 1, Fig 3A), but further suggested that the methylfolate trap may induce the intrinsic bactericidal activity of SULFA drugs.

To further characterize the methylfolate trap in *M*. *tuberculosis*, we used CDC1551, a clinical strain isolated in a 1994–1996 tuberculosis outbreak in the United States [39], for constructing several strains related to methylfolate trap formation (S2 Table). CDC1551 is a natural *metH* deletion mutant due to a 1,196-bp truncation located at the 3’-terminus of its encoding gene (*mt2183*) (Fig 3C) [38, 40]. Similar to the *M*. *smegmatis* methylfolate trap mutants, colonies of CDC1551 displayed a “white” morphology, differing from the yellow appearance of H37Rv, which resembles wild type *M*. *smegmatis* (S6 Fig). To better understand the molecular mechanisms affecting trap formation, SULFA sensitivity tests were performed with a minimal medium (Dubos) and a gradient of increasing B_12_ concentrations (Fig 3D). In the absence of exogenous B_12_, CDC1551 (numbered 2) displayed higher SULFA sensitivity compared to the CDC1551 strain *in trans* expressing the intact *metH* gene from H37Rv (CDC1551/*metH*, numbered 1), indicating that, unlike H37Rv, the B_12_ biosynthesis is functional in CDC1551 (Fig 3D). The level of internally synthesized B_12_ was likely enough to partially repress the expression of *metE* and to activate MetH activity (see Discussion). When *cobIJ* was deleted (CDCΔ*cobIJ*/*metH* and CDCΔ*cobIJ*, numbered 3 and 4 respectively), SULFA resistance increased (Fig 3D), possibly due to the derepression of *metE* in the complete absence of B_12_ (similar to H37Rv in minimal medium). Deletion of *bacA* (numbered 5 and 6), encoding the B_12_ uptake system in *M*. *tuberculosis* [37], did not have any effect in this condition (far left panel). In the presence of as low as 0.25 μM B_12_, *metE* expression appeared to be further suppressed, making CDC1551 highly susceptible to SMZ compared to CDC1551/*metH* (Fig 3D, second panel from left). The higher the concentration of exogenous B_12_, the less SULFA resistance was displayed by CDCΔ*cobIJ*, most likely due to increased suppression of *metE*. This was not seen in the case of CDCΔ*cobIJ*/*metH* since MetH was further activated in the presence of B_12_, thus compensating for *metE* suppression. Unlike CDC1551, CDCΔ*bacA* did not show a severe reduction in SULFA resistance when B_12_ was added due to its lack of B_12_ uptake activity. Similarly, but conversely, CDCΔ*bacA*/*metH* did not show an increased SULFA resistance compared to CDC1551/*metH* in response to exogenous B_12_. Most importantly, as seen with the H37Rv background (Fig 3A), exogenous methionine did not enhance the SULFA resistance of CDC1551-derived strains (Fig 3D).

Previous studies suggested that *M*. *tuberculosis* is able to uptake and metabolize B_12_ from its host [41]. To evaluate if the methylfolate trap can form thus affecting the SULFA sensitivity of *M*. *tuberculosis* residing within macrophages, strains were used to infect the macrophage cell line J774.A1, grown in a medium containing 10% fetal bovine serum. The infected macrophages were treated with SMZ, followed by serial plating of the intracellular bacteria and c.f.u. counting. In both the H37Rv (Fig 3E) and the CDC1551 backgrounds (Fig 3F), strains lacking *metH* exhibited significantly increased sensitivity to SULFA treatment. *In trans* expression of H37Rv *metH* (*rv2124c*) restored SULFA resistance to both RvΔ*metH* and CDC1551 (Fig 3E and 3F). As previously suggested [39], the proliferation of CDC1551 in macrophages in the absence of SMZ was much faster compared to H37Rv (Fig 3F). However, its survival was more severely reduced compared to H37Rv when the infected macrophages were treated with SMZ (Fig 3F). This enhanced bactericidal activity of SMZ against CDC1551 was reduced in CDC1551/*metH*, confirming the correlation of MetH activity and the intrinsic resistance of CDC1551 to SULFAs.

Together, these results demonstrated that (*i*) the methylfolate trap, when successfully formed, can sensitize *M*. *tuberculosis* to SULFAs both *in vitro* and during infection of host macrophages, (*ii*) the methylfolate trap promotes the bactericidal activity of SULFA drugs, (*iii*) because of its non-functional B_12_ biosynthetic pathway, H37Rv relies on its uptake system to obtain exogenous B_12_, (*iv*) trace amounts of B_12_ are sufficient to suppress *metE* expression giving *metH* a more important role in preventing methylfolate trap formation, and (*v*) because of its truncated *metH* gene, CDC1551 is intrinsically more susceptible to methylfolate trap formation, rendering it more sensitive to SULFAs both *in vitro* and during macrophage infection [42] (Fig 3D and 3F). Our laboratory is currently investigating how mutations in *metH* and genes involved in B_12_ biosynthesis affect SULFA sensitivity among *M*. *tuberculosis* clinical isolates.

### Methylfolate trap in Gram-negative bacteria

To assess if the methylfolate trap plays a similar role in SULFA sensitivity in Gram-negative bacteria, we investigated its role in a selected group of significant pathogens with distinct metabolic capacities.

**Fig 4:**
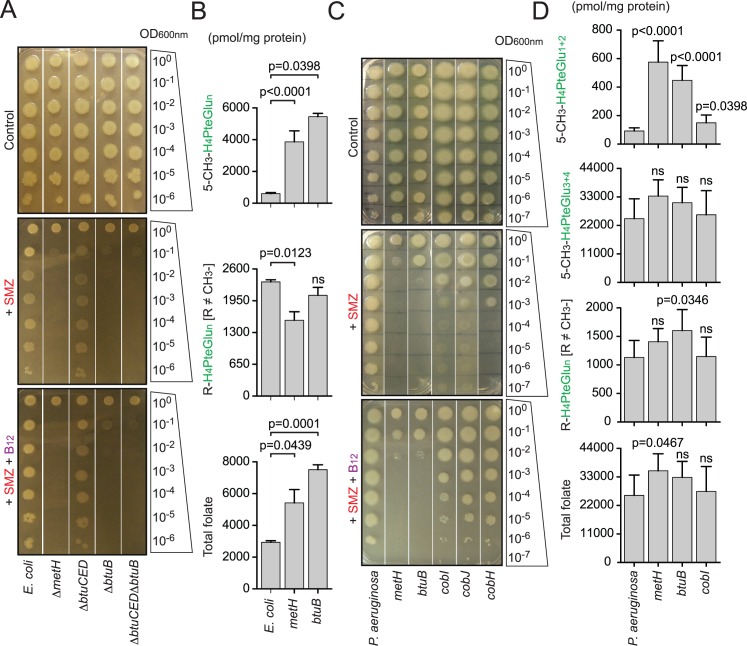
Methylfolate trap and its role in Gram-negative bacteria. (**A**) SULFA susceptibility in *Escherichia coli* strains was analyzed by 10-fold serial dilutions. 5 μl of 10X diluted cell suspensions starting from OD1 were spotted on LB agar in the absence or presence of 125 μg/ml SMZ. Exogenous B_12_ was added at 2 nM final concentration. Growth was recorded after 48 h of incubation at 37°C. (**B**) Effect of *metH* and *btuB* on the folate pool of *E*. *coli*. Growing cultures (OD1) of *E*. *coli* strains were treated with 2.5 mg/ml SMZ for 15 min followed by folate extraction and LC-MS/MS analysis. Data shown, from top to bottom, are the combined levels of all 5-CH_3_-H_4_PteGlu_n_ species, all non-methylated folate species, and the total folate, respectively. Bars represent means of biological triplicates with standard deviations. ns, no significant difference between the indicated mutant and wild type *E*. *coli*. (**C**) Role of the methylfolate trap in SULFA sensitivity of *Pseudomonas aeruginosa* strains. Cultures underwent 10-fold serial dilutions, and 5 μl of diluted cultures were spotted onto solid media in the absence or presence of 150 μg/ml SMZ. Exogenous B_12_ was added at 2 nM final concentration. Growth was recorded after 48 h of incubation at 37°C. (**D**) Effect of *metH*, *btuB* and *cobI* on the folate pool of *P*. *aeruginosa*. Growing cultures (OD1) of *P*. *aeruginosa* strains were treated with 2.5 mg/ml SMZ for 15 min followed by folate extraction and LC-MS/MS analysis. Data shown, from top to bottom, are the combined levels of mono- and di-glutamylated methyl folate species (5-CH_3_-H_4_PteGlu_1-2_), tri- and tetra-glutamylated methyl folate species (5-CH_3_-H_4_PteGlu_3-4_), all non-methylated folate species, and the total folate. Bars represent means of biological triplicates with standard deviations. ns, no significant difference between the indicated mutant and wild type *P*. *aeruginosa*.

Similar to the *M*. *tuberculosis* H37Rv strain, *E*. *coli* does not synthesize B_12_, instead it imports the vitamin via the transport system BtuBCED [43, 44]. Whereas mutations in *btuC*, *btuE*, and/or *btuD* partially reduce uptake, mutations in *btuB* completely abolish B_12_ transport [45]. On a complex medium, an *E*. *coli* Δ*btuCED* (*b1711*, *b1710*, and *b1709*, respectively) triple mutant remained SULFA resistant, whereas Δ*metH* (*b4019*), Δ*btuB* (*b3966*), and a Δ*btuB*Δ*CED* quadruple mutant all became hypersusceptible (Fig 4A, Table 1). In serial dilution-spot tests using 125 μg/ml SMZ, these mutants displayed >10^4^ times increased susceptibility compared to wild type BW25113 (Fig 4A). Exogenous B_12_ was unable to restore SMZ resistance in these mutants due to the absence of MetH or B_12_ transport activity (Fig 4A). The increased SULFA sensitivity was verified by measuring minimal inhibitory concentrations (MIC, Table 1), which is defined as the lowest concentration of an antibiotic that inhibits the visible growth of bacteria. To demonstrate methylfolate trap formation at the metabolic level, *E*. *coli* cultures were treated with SMZ and total folate was immediately extracted and analyzed by LC-MS/MS [12]. As shown in Fig 4B, 5-CH_3_-H_4_PteGlu_n_ markedly accumulated in Δ*metH* and Δ*btuB* compared to the parental strain, confirming methylfolate trap formation. Because of its inability to synthesize B_12_ *de novo*, *E*. *coli* relies entirely on import to prevent the methylfolate trap.

*P*. *aeruginosa* is capable of not only synthesizing *de novo* but also importing B_12_ from the environment. Transposon mutants with insertions in genes encoding *metH* (PA1843), *cobI* (PA2904), *cobJ* (PA2903), *cobH* (PA2905) and *btuB* (PA1271) were obtained from the Pseudomonas Transposon Mutant Collection (Manoil Laboratory, University of Washington Genome Sciences) [46] (S2 Table). The mutants were subjected to antifolate susceptibility tests, followed by folate analysis as described above. All *P*. *aeruginosa* mutants became more susceptible to SULFA drugs on a complex medium (Fig 4C, Table 1). The *P*. *aeruginosa metH* and *btuB* mutants displayed identical, and the most severe susceptibility to SULFAs. These strains were at least 10^5^ times more susceptible than wild type as revealed by serial dilution-spotting assays using 125 μg/ml SMZ (Fig 4C). *cob* mutants were less susceptible compared to these two strains, suggesting that B_12_ import is more important than *de novo* synthesis in the condition tested (Fig 4C, Table 1). Indeed, exogenous B_12_ reinstated growth of the *cob* mutants but failed to do the same for *metH* and *btuB* (Fig 4C). Chemical analyses also revealed accumulation of the methylfolate trap marker, 5-CH_3_-H_4_PteGlu_n_, in both *metH* and *btuB* (Fig 4D).

Similar experiments with *S*. *typhimurium* strains (John Roth Laboratory, UC Davis, S2 Table) confirmed the correlation of the methylfolate trap and increased SULFA susceptibility in bacteria (Table 1, S7 Fig, and further studies below).

### Dynamics of folate and related metabolites during methylfolate trap formation

**Fig 5:**
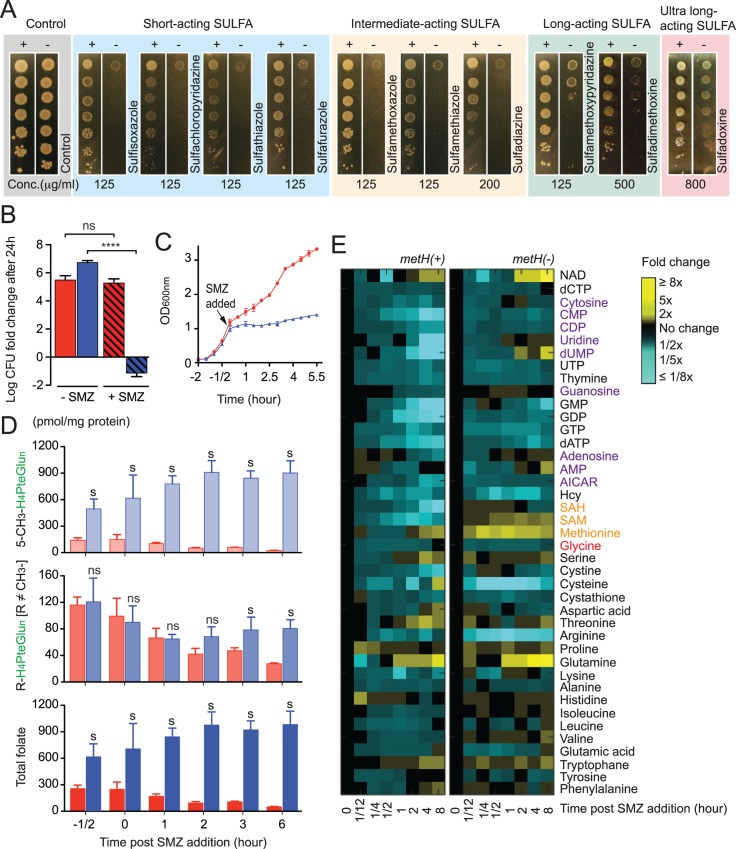
Metabolic dynamics of the methylfolate trap in *Salmonella typhimurium* SULFA resistance. (**A**) Wide-spectrum SULFA susceptibility of *S*. *typhimurium metH*(+) and *metH*(-) analyzed by 10X serial dilution. Cultures were diluted starting with OD1 and 5 μl cell suspensions were spotted onto LB agar in the absence (control) or presence of different SULFAs, used at the indicated concentrations. These SULFA drugs are classified into all four subgroups, in left-right order: short-acting (blue), intermediate-acting (yellow), long-acting (green), and ultra-long-acting (pink), respectively. Growth was recorded after 48 h at 37°C. (**B**) Viability of *S*. *typhimurium metH*(+) (red) and *metH*(-) (blue) on LB agar 24 h post-SMZ addition (125 μg/ml). Colony forming units (c.f.u.) were determined and normalized to c.f.u. values of the inoculation input (0 h). The y-axis represents c.f.u. fold-change on a log10 scale of SMZ-treated (+SMZ, hatched bars) and control non-treated samples (-SMZ, empty bars). Error bars represent standard deviations from biological triplicates. **** p<0.0001; ns, no significant difference. (**C**) SULFA susceptibility of *S*. *typhimurium* strains in liquid LB medium. Cultures of *metH*(+) (red) and *metH*(-) (blue) growing at OD1 was added with 2.5 mg/ml SMZ (arrow). Growth was monitored by measuring OD_600_. (**D**) Dynamics of the folate pool in *S*. *typhimurium metH*(+) (red) and *metH*(-) (blue) strains. At selected time points following SULFA treatment, cells were collected and folate extracted and analyzed by LC-MS/MS. Bars represent the combined levels of all 5-CH_3_-H_4_PteGlu_n_ species (top), all non-methylated folate species (middle), and total folate (bottom) following SMZ addition. s, significant difference between *metH*(-) and corresponding *metH*(+) samples; ns, no significant difference. (**E**) Dynamics of 41 metabolites in *metH*(+) (upper) and *metH*(-) (lower) strains. Metabolites are shown with their fold change over time (0–8 hours post SMZ addition). At selected time points following SMZ treatment, cells were collected and metabolites extracted and analyzed by LC-MS/MS. Signal intensity was normalized to OD_600nm_ at each time point. Relative levels are expressed as the log ratio of the normalized signal intensity of SMZ-treated cells at each time point to the normalized signal intensity of the no drug control sample at t = 0 (n = 3). The data shown in all figures represents the mean of biological repeats (n ≥ 3) with standard deviations. In the experiments demonstrated in Fig 5C–5E, SMZ was added at 2.5 mg/ml when cultures reached OD1.

Similar to *M*. *smegmatis* and other Gram-negative bacteria, the deletion of *metH*, but not *metE*, resulted in the methylfolate trap and reduced SULFA resistance in *S*. *typhimurium* on complex media (S7 Fig). The absence of *metH*, hence the methylfolate trap, led to increased susceptibility to SULFA drugs classified in all categories (Fig 5A), but not to folate-unrelated antibiotics (S8 Fig). To investigate if the effect of the methylfolate trap was bactericidal or bacteriostatic, *S*. *typhimurium metH*(+) and *metH*(-) strains were spotted on filters (~10^4^ cells/filter), which were placed on the surface of Luria-Bertani (LB) agar plates supplemented with or without SMZ. Following 24 h of incubation at 37°C, cells from the inoculated filters were resuspended, and colony forming units (c.f.u.) were measured by serial dilution and plating. On LB agar free of SULFA, both *metH*(+) and *metH*(-) proliferated to 10^6^ times more cells than the input (Fig 5B). In the presence of 125 μg/ml SMZ, growth of *metH*(+) was normal whereas only 0–8.5% of the *metH*(-) input survived (Fig 5B), indicating an enhanced bactericidal effect of SMZ due to the methylfolate trap. In liquid LB, addition of 2.5 mg/ml SMZ similarly reduced growth of *metH*(-) while still allowing growth of *metH*(+) (Fig 5C), confirming the correlation between SULFA resistance and MetH activity.

To investigate if the increased susceptibility was due to enhanced import, the SULFA uptake of *S*. *typhimurium* strains was measured using radioactive SMZ. However, both *metH*(+) and *metH*(-) displayed identical uptake following the addition of SMZ to the medium (S9 Fig, panel A). We next examined the effect of the methylfolate trap on the synthesis of macromolecules (DNA, RNA and protein) during SULFA treatment. Cells of *metH*(+) or *metH*(-) bacteria, growing in the presence of SMZ, were labeled using [^3^H]-thymidine, [^3^H]-uracil, or [^35^S]-methionine, respectively. While DNA and protein synthesis were not affected by the methylfolate trap during SULFA treatment, RNA synthesis was significantly reduced in cells suffering the metabolic blockage (S9 Fig, panels B-D).

To assess changes in the folate pool during SULFA-induced methylfolate trap formation, *S*. *typhimurium* cells growing in liquid LB medium were treated with SMZ, followed by sample collection. Folate was extracted and individual species quantified using LC-MS/MS. In the presence of MetH, combined levels of both methylated (5-CH_3_-H_4_PteGlu_n_) and non-methylated folate species (R-H_4_PteGlu_n_ R ≠ CH_3_) immediately and continuously declined in response to SMZ (Fig 5D, top and middle panels, red bars; see also S10 Fig for the dynamics of individual species). In contrast, in *metH*(-) cells, 5-CH_3_-H_4_PteGlu_n_ gradually accumulated following SMZ treatment (Fig 5D, top panel, blue bars). Levels of non-methylated folate species in *metH*(-) gradually declined for the first hour, then remained constant for the remainder of the experiment (Fig 5D, middle panel, blue bars). This result indicated possible cellular feedback, either through an increase in *de novo* H_4_PteGlu_n_ synthesis or rearrangement in the inter-conversion network of one-carbon metabolism.

**Fig 6:**
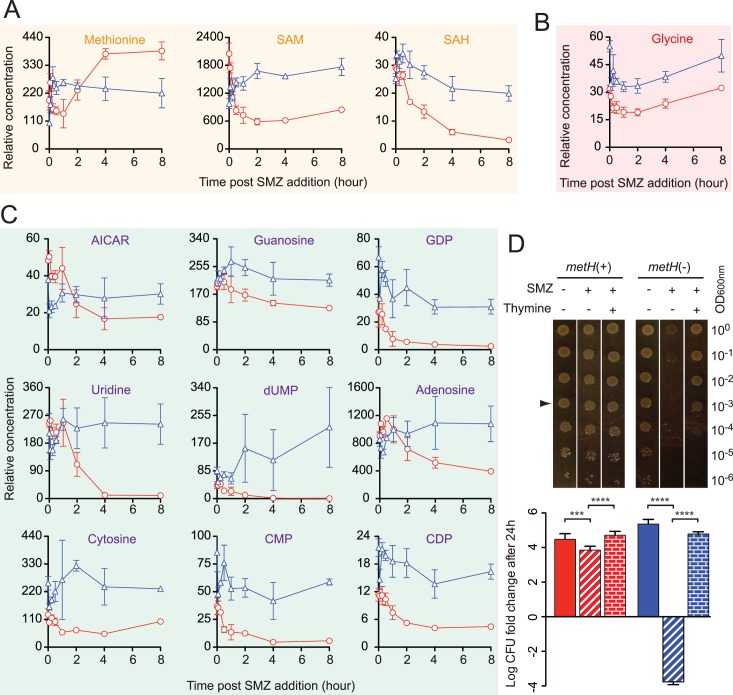
Methylfolate trap-mediated thymineless death. (**A**) Cellular levels of methionine, SAM, and SAH in *S*. *typhimurium* cells following SMZ treatment. *metH*(-) (blue triangle) displayed a lower level of methionine but higher levels of SAM and SAH than its parent *metH*(+) (red circle). (**B**) Higher level of glycine in *metH*(-) (blue triangle) compared to *metH*(+) (red circle). (**C**) Dynamics of nucleotide pool in *S*. *typhimurium* cells during SMZ treatment. While the levels of nucleotides and intermediates were sharply reduced in response to SMZ in *metH*(+) (red circle), cells of *metH*(-) (blue triangle) failed to deplete these metabolites. (**D**) Thymine abolishes SULFA-induced cell death and restores growth in *metH*(-). *Salmonella* cultures were 10X serially diluted and 5 μl of diluted cultures were spotted on LB agar in the absence or presence of 125 μg/ml SMZ and 2 mM thymine. Growth on test plates (top panel) was recorded after 24 h of incubation at 37°C. Corresponding 24-hour viability of colonies grown from spotted OD0.001 cell suspensions (arrow) was determined by measuring c.f.u. and normalized to c.f.u. values of the input inoculum (lower panel). The y-axis represents c.f.u. fold-change on a log10 scale. Bars represent standard deviations from biological triplicates. *** p<0.001; **** p<0.0001.

To further analyze metabolic alterations in response to such folate homeostatic defects, post-SMZ treatment levels of 41 metabolites were profiled using LC-MS/MS-based metabolomics. Cells were sampled from growth curves similar to those in Fig 5C from which metabolites were extracted and analyzed by the Metabolomics Lab at the Roy J. Carver Biotechnology Center (University of Illinois at Urbana-Champaign). Metabolic abnormalities caused by the SMZ-induced methylfolate trap include the accumulation of intermediates within the methionine-homocysteine cycle (Figs 5E and 6A, orange), glycine (Figs 5E and 6B, red) and nucleotides (Figs 5E and 6C, purple), as discussed in more detail below.

### Thymineless death caused by the methylfolate trap

The MetH reaction connects the one-carbon metabolic network with the methionine cycle through its conversion (methylation) of Hcy to methionine (S9 Fig, panel E). Therefore, impaired MetH would lead to the accumulation of not only 5-CH_3_-H_4_PteGlu_n_, but also Hcy, causing hyperhomocysteinemia. In the cell, Hcy is further converted to Hcy-thiolactone, which is cytotoxic due to its interaction with physiologically important proteins [47, 48]. Because it is neutral at physiological pH (pKa = 6.67), Hcy-thiolactone is steadily secreted into exogenous media following its production from Hcy [48]. Besides harvesting cells for folate and metabolomic analyses (Fig 5D and 5E), culture filtrates from *metH*(+) and *metH*(-) growing in the presence of SMZ were also collected for Hcy-thiolactone analysis (S1 Text) [49]. As shown in Fig 6A, cells of *metH*(-) accumulated *S*-adenosylhomocysteine (SAH), which led to higher levels of Hcy-thiolactone in the medium compared to *metH*(+) (S9 Fig, panel F).

In the presence of MetH (red circle), production of methionine (Fig 6A) and glycine (Fig 6B) rapidly dropped while levels of nucleotides (Fig 6C) including aminoimidazole carboxamide ribonucleotide (AICAR), a precursor of purine synthesis, slightly increased during the first half an hour to one hour of SMZ treatment. Thereafter, synthesis of methionine and glycine resumed but nucleotides underwent continuous depletion. In the absence of MetH (blue triangle), methionine synthesis slightly increased (Fig 6A), most likely due to increased uptake, nucleotides levels also increased (Fig 6C), but glycine levels slightly declined (Fig 6B) in the first hour. After this time period, nucleotides, especially dUMP, remained highly elevated, methionine levels declined and remained constant while glycine levels increased and remained elevated.

Antifolate-responsive depletion of intracellular glycine and purines was recently proposed as an *E*. *coli* mechanism to escape thymineless death [15]. To test if thymine plays a role in the methylfolate trap-promoted bactericidal activity of SULFA, this nucleotide precursor was added to medium and the survival of strains was evaluated by serial dilution and plating method. Interestingly, thymine abolished the SULFA-induced death of the *metH*(-) strain, and restored its growth (Fig 6D). These results suggest that the methylfolate trap promotes the intrinsic thymineless death of bacteria by SULFA drugs, by causing an imbalance in nucleotide levels while preventing cellular depletion of glycine.

### Methylfolate trap-mediated SULFA sensitization in a monocyte infection model

**Fig 7:**
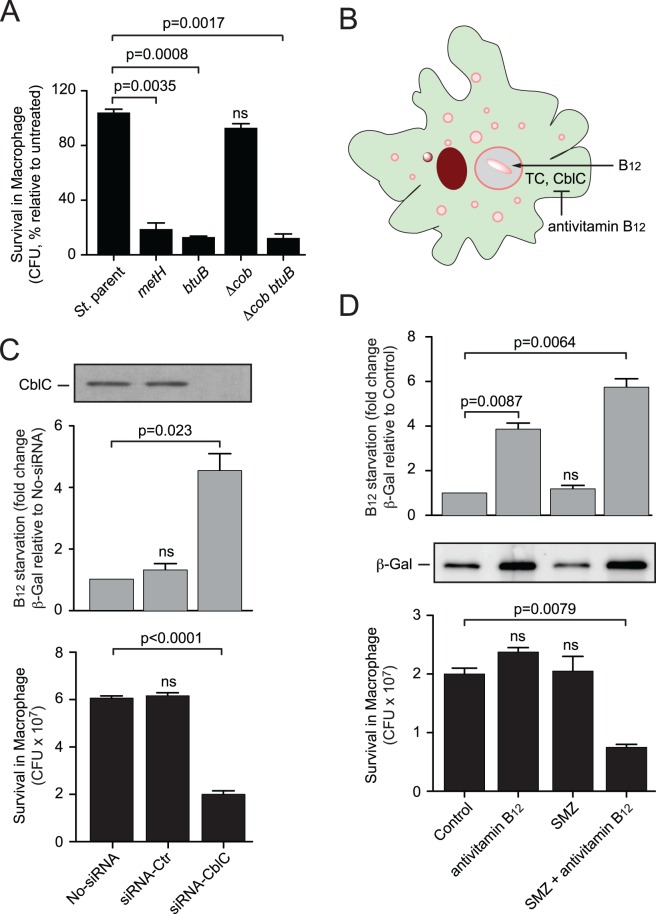
Genetic and chemical induction of the methylfolate trap during *Salmonella* infection of macrophages. (**A**) Survival of *Salmonella* strains in macrophages treated with SULFAs. Macrophages J774A.1 were infected for 1 h followed by 18 h chase, during which cells were untreated or treated with 1 mg/ml SMZ. Colony forming units (c.f.u.) were determined by serial dilution and plating method. (**B**) Cellular uptake and conversion of exogenous B_12_ in mammalian cells requires transcobalamin (TC) and CblC proteins, respectively. Antivitamin B_12_ molecules such as EtPhCbl inhibit transcobalamin and CblC, thereby restricting B_12_ bioavailability to intracellular bacteria. (**C**) Depletion of CblC expression, detected by Western Blot using a specific antibody (top), caused B_12_ starvation (middle) and increased SULFA sensitivity (bottom) of intracellular *Salmonella*. siRNA transfected THP-1 macrophages were infected with *S*. *typhimurium* cells expressing β-galactosidase from a B_12_ starvation-responsive promoter for 1 h, followed by 18 h chase, during which the infected macrophages were treated without or with 1 mg/ml SMZ. B_12_ starvation was estimated by determining β-galactosidase activity while *Salmonella* survival measured by c.f.u. counting. (**D**) Chemical restriction of B_12_ sensitizes intracellular *S*. *typhimurium* to SULFA treatment. Macrophages J774A.1 were infected with *S*. *typhimurium* cells harboring a B_12_ molecular probe for 1 h followed by 18 h chase, during which cells were untreated or treated with 1 mg/ml SMZ or/and 50 nM EtPhCbl. B_12_ starvation was estimated through measuring enzymatic activity (top) and expression of β-galactosidase by Western Blot (middle). *Salmonella* survival from the corresponding macrophages was measured through c.f.u. counting (bottom). Error bars represent standard deviations from biological triplicates. ns, no significant difference compared to control groups.

To investigate if the methylfolate trap renders bacteria more susceptible to SULFAs in a host cell environment, we first monitored the intracellular survival of *S*. *typhimurium* strains in J774A.1, a macrophage cell line commonly used for antibiotic sensitivity testing [50]. When the infected macrophages were treated with SMZ at a concentration sub-inhibitory for the *S*. *typhimurium* parental strain, mutants undergoing the methylfolate trap displayed significant defects in survival (Fig 7A). The survival of the *S*. *typhimurium* strains in macrophages resembled the patterns of *in vitro* sensitivity (S7 Fig), suggesting a similar role of the methylfolate trap in promoting SULFA susceptibility of intracellular bacteria.

To assess if SULFA susceptibility of the intracellular bacteria can be promoted through pharmacological induction of the methylfolate trap, we sought to restrict B_12_ bioavailability using a chemical approach (Fig 7B). The cellular uptake and conversion of exogenous B_12_ (cyanocobalamin) to biologically active cofactors (adenosylcobalamin and methylcobalamin) in mammalian cells requires the enzymatic activity of CblC, also known as MMACHC (for methylmalonic aciduria (cobalamin deficiency) *cblC* type, with homocystinuria) [51]. To investigate if B_12_ bioavailability, hence SULFA sensitivity, of intracellular *S*. *typhimurium* could be controlled through CblC inhibition, expression of *cblC* in macrophages THP-1 was depleted using RNA interference. Transfection with *cblC*-specific siRNA effectively reduced CblC expression, detected by Western Blot using a CblC monoclonal antibody (Fig 7C, top panel). The reduced *cblC* expression was found to correlate with increased B_12_ starvation of the intracellular *S*. *typhimurium* bacillus as detected by a B_12_ molecular probe (Fig 7C, middle) [52]. Within such CblC-depleted macrophages, *S*. *typhimurium* became more SMZ susceptible as determined by c.f.u plating assays (Fig 7C, bottom).

We recently developed Co_β_-4-ethylphenylcob-(III)alamin (EtPhCbl) [53], a cobalamin analog that can function as a vitamin B_12_ antagonist (or “antivitamin B_12_”) [53, 54]. EtPhCbl effectively binds to CblC but resists dissociation from the protein, thereby blocking CblC from its normal functions of decyanation and dealkylation of newly internalized cyanocobalamin and methylcobalamin, respectively [55, 56]. Because bacteria do not have CblC homologs, EtPhCbl had no effect when used directly on bacterial cells (S11 Fig). To test whether EtPhCbl increases methylfolate trap-mediated SULFA susceptibility in bacteria residing within host cells, macrophages were first infected with *S*. *typhimurium*. Thereafter, the infected cells were treated with SMZ, EtPhCbl, or their combination. Cells were then lysed and intracellular bacteria determined by c.f.u. plating assays. Whereas SMZ or EtPhCbl alone did not affect the intracellular survival of *S*. *typhimurium*, their combination resulted in both B_12_ starvation (Fig 7D, top and middle), and a significant c.f.u. reduction (Fig 7D, bottom) to the intracellular bacillus.

## Discussion

We first constructed a large library of transposon insertion mutants in *M*. *smegmatis*. The size of the library was approximately 2 times the number of genes in the *M*. *smegmatis* genome (6,717 protein-coding genes and 54 RNA-coding genes, http://www.genome.jp/kegg-bin/show_organism?org=msm). Screening this library, we identified 50 chromosomal loci responsible for the intrinsic antifolate resistance in *M*. *smegmatis* (S1 Table). Further investigation of the inserted genes revealed many novel pathways previously unknown to be involved in bacterial intrinsic antifolate resistance. For example, we recently reported the role of 5,10-methenyltetrahydrofolate synthase (MTHFS, encoded by *msmeg_5472*), which converts 5-CHO-H_4_PteGlu_n_, a proposed storage form of folate, to 5,10-CH^+^-H_4_PteGlu_n_, in cellular folate homeostasis and bacterial antifolate resistance [12]. These studies, including the current work reported in this paper, confirm the richness of potential drug targets in this pathway as previously postulated [57, 58]. The fact that many loci were repeatedly identified in the screen confirmed the saturation of the library. It is however important to note that our screening procedures only selected mutants that showed “normal growth” in the absence of antifolates; thus resistance determinants encoded by essential genes or genes whose mutation affected *M*. *smegmatis* growth on NE medium in the absence of antifolates were not included in this pool.

In further studies of the mutant library, we have now discovered another novel mechanism of intrinsic SULFA resistance in bacteria referred to as the methylfolate trap, which occurs when cellular H_4_PteGlu_n_ is trapped in a single methylated form, 5-CH_3_-H_4_PteGlu_n_ [21, 59]. We show that the methylfolate trap increases the bactericidal activity of SULFA drugs against mycobacteria and Gram-negative bacteria. The methylfolate trap hypothesis was first proposed by Herbert and Zalusky in 1962 to explain the cause of megaloblastic anemia observed in patients deficient in folate and vitamin B_12_ [19]. Besides the typical low blood count and macrocytosis, cells from those patients encountered a “pseudo-folate deficient” state, in which folic acid injected into tissues rapidly disappeared while 5-CH_3_-H_4_PteGlu_n_ “piled up” in sera. However, simultaneous treatment of many of those patients with vitamin B_12_ immediately corrected the folate leakage and blood count normalized [20, 59, 60]. These phenomena were hypothesized to be a result of deficiencies in the B_12_-dependent methionine synthase (MetH) activity, which converts 5-CH_3_-H_4_PteGlu_n_ and Hcy to H_4_PteGlu_n_ and methionine, respectively. This hypothesis was supported by the fact that all patients with inborn genetic errors in the *metH* gene suffer from anemia or developmental delay; and exhibit accumulation of 5-CH_3_-H_4_PteGlu_n_ and Hcy [61, 62]. However, direct genetic evidence connecting *metH* and the methylfolate trap has not been established because constructing *metH* knockout mice has proved unsuccessful thus far [27]. Nonetheless, the methylfolate trap hypothesis is now widely accepted to explain the relationships of B_12_, folate, and Hcy homeostasis in many human diseases [63].

The methylfolate trap and its physiological consequences have never been described in bacteria, plants or microbial eukaryotes, possibly because these organisms are able to synthesize folate *de novo*, thus minimizing the trap’s effects. Interestingly, our data show that the methylfolate trap is lethal to bacteria when it is formed in the presence of SULFA drugs, which inhibit *de novo* folate biosynthesis. Due to the lack of *de novo* folate synthesis, mammalian cells undergoing the methylfolate trap exhibit a depletion of non-methyl folate species, consequently leading to reduced synthesis of amino acids and nucleotides from the one-carbon metabolic network. By contrast, the levels of non-methyl folate species in bacterial cells experiencing the trap only modestly reduced or did not change, while total folate elevated because of the increase in 5-CH_3_-H_4_PteGlu_n_ levels (Figs 2C, 4B, 4D and 5D). This was most likely due to an increase in *de novo* folate synthesis in response to the continuous loss of folate molecules trapped in the irreversible 5-CH_3_-H_4_PteGlu_n_ form. Such a response leads to two possible lethal consequences: (*i*) a wasteful cycle of synthesis and loss of H_4_PteGlu_n_ which rapidly depletes cellular resources, or (*ii*) an uncoordinated increase in activity of the early steps preceding the MetH reaction in the one-carbon metabolic network (Fig 1A). Because thymidylate synthase is a rate-limiting reaction, such an increase in H_4_PteGlu_n_ influx in the absence of MetH would lead to increased synthesis of some amino acids and nucleotides while levels of thymidine nucleotides remain low, thus promoting “unbalanced” growth that causes thymineless death [13]. Our metabolomic data shed light on these possibilities. Besides the extracellular accumulation of Hcy-thiolactone (S9 Fig, panel F), which may be deleterious to exogenously functioning molecules, cells undergoing the methylfolate trap were unable to deplete glycine and nucleotides (Fig 6B and 6C). Cellular depletion of glycine and purines was found necessary for bacterial escape from thymineless death, a known contributor to the bactericidal activity of antifolates [15, 16]. Although thymidine triphosphate (dTTP) was not detectable in cells subjected to our experimental conditions, the level of deoxyuridine monophosphate (dUMP), a precursor of dTTP, increased 700 fold in the absence of MetH after 8 hours of SMZ treatment (Fig 6C, 219.26 in *metH*(-) versus 0.31 in *metH*(+), p = 0.0371), indicating low activities of thymidylate synthase (TS, Fig 1A) in the presence of the methylfolate trap. Cellular accumulation of dUMP, leading to robust dUTP production, has been known to contribute to thymineless death by causing misincorporation of uracil into DNA [64]. Importantly, exogenous supplementation of thymine completely abolished the SULFA-induced death in *metH*(-) (Fig 6D). In addition, cells suffering the methylfolate trap displayed unchanged synthesis of proteins and DNA but reduced synthesis of RNA (S9 Fig, panel C), a hallmark exhibited by bacterial cells that undergo thymineless death [65, 66]. Together, our studies suggest that the methylfolate trap boosts the bactericidal activity of SULFAs by inducing thymineless death.

It is important to note that many bacteria also encode MetE, a B_12_-independent methionine synthase [35]. However, catalytic activity of MetE is more than a hundred fold lower than that of MetH [67, 68], and the expression of *metE* is sensitive to B_12_ exposure [38], making MetH the dominant methionine synthase. In fact, our data indicated that neither deletion nor overexpression of *metE* affected SULFA susceptibility in *M*. *smegmatis* (S4 Fig and S5 Fig) and *S*. *typhimurium* (S7 Fig), and that *de novo* synthesized B_12_ contributes to partially inhibiting *metE* expression in autotrophic bacteria. In the complete absence of exogenous B_12_ in minimal media, B_12_ auxotrophic bacteria such as the *M*. *tuberculosis* laboratory strain H37Rv are able to use MetE activity to prevent trap formation. However, exposure to minute amounts of B_12_ is enough to suppress *metE* expression. With previous studies showing that functionally adequate levels of B_12_ are accessible to bacterial pathogens during vertebrate host infections [41], the role of MetE in the methylfolate trap-mediated SULFA sensitization is likely negligible. The fact that *metH* deletion leads to increased SULFA sensitivity in H37Rv during macrophage infection (Fig 3E) further suggested that this bacterium is able to acquire B_12_ from the host cell, and that the acquired B_12_ is sufficient for preventing methylfolate trap formation.

Similar to mammalian cells, bacteria undergoing restricted *de novo* folate synthesis caused by SULFAs relied on vitamin B_12_ for preventing methylfolate trap formation. Accordingly, reduced B_12_ bioavailability could sensitize some bacterial pathogens to SULFAs. Our experiments presented in Fig 7 provide a proof-of-concept that this folate antagonistic strategy, namely the chemical promotion of the methylfolate trap, is feasible for inducing the killing of pathogenic bacteria by SULFAs. However, targeting B_12_ bioavailability by general antivitamin B_12_ molecules may not be effective for some bacteria, providing the heterogeneity of B_12_ biosynthesis and uptake. In addition, it is currently not known if such antivitamin B_12_ compounds play a role in the regulation of B_12_ synthesis or uptake in the targeted bacterial pathogens. Another challenge is how to develop methylfolate trap inducers that are specific for bacteria, thus causing no significant toxicity to mammalian cells. In this regard, targeting bacterial proteins involved in B_12_ uptake and salvage, which are distinct from those of the mammalian counterparts, may provide a possible strategy. As we have previously proposed [12, 57, 58], antifolate resistance determinants such as the methylfolate trap represent potential targets for the development of SULFA boosters, which not only protect the efficacy of SULFAs but also increase their potency against drug resistant pathogens. With the increasing use of co-trimoxazole (SMZ plus TMP) in prophylactic treatments of HIV positive patients throughout the world [10], such SULFA boosters are urgently needed.

## Materials and Methods

### Bacterial strains, plasmids, primers, and growth media

Strains, plasmids, and primers used in this study are listed in S2, S3 and S4 Tables of the Supporting Information, which also contain information on the genetic screen and identification of antifolate-sensitive mutants, targeted gene deletion, genetic and chemical complementation, extraction and analysis of cellular folate derivatives, and antibiotic susceptibility tests (S1 Text). *M*. *smegmatis* mc^2^155 and its derived transposon mutants were grown in LB broth or 7H9 (Difco) supplemented with glucose and 0.5% Tween 80. *M*. *tuberculosis* strains were grown in 7H10-OADC or Dubos-ADC media (Difco). Unless otherwise stated, Gram-negative bacteria were grown in LB broth or LB agar.

### Statistical analysis

Statistical analyses were conducted using GraphPad Prism 5.0f software (La Jolla, CA). Students two-tailed *t*-test was used to analyze the statistical significance of differences between groups.

### Addition methods

Other methods used in this study can be found in the Supporting Information (S1 Text).

## Supporting Information

S1 Fig *Himar1* insertions and MetH truncation mutants in *M*. *smegmatis*. (**A**) *Himar1* insertion into the *metH* (*msmeg_4185*) gene in 124H4, 63H1, 121D7 and 58B10. Arrows indicate the positions of the TA dinucleotides where *Himar1* inserted. (**B**) Domain alignment of MetH truncation mutants compared to wild type using PROSITE (http://prosite.expasy.org). The truncated proteins in 58B10 and 121D7 are similar to that of CDC1551 shown Fig 3C.
(PDF)

S2 Fig Role of *metH* in *M*. *smegmatis* SULFA resistance. A representative disc diffusion test shows the effect of *metH* deletion on *M*. *smegmatis* SULFA resistance. Cells of wild type (top left), *Ms*Δ*metH* (top right), and complemented strain (bottom right) were seeded onto the surface of NE medium. Discs containing SULFA drugs classified in different subgroups were applied at the positions indicated in the bottom left panel. Colors indicate the groups to which the antibiotics belong. Non-SULFA antifolates were included as controls.
(PDF)

S3 Fig Role of *metH* in *M*. *smegmatis* susceptibility to non-antifolates. A representative disc diffusion test shows that *metH* is not involved in *M*. *smegmatis* resistance to non-antifolate drugs. Cells of wild type (top left) and *Ms*Δ*metH* (top right) were seeded onto the surface of NE. Antibiotic discs were applied at the positions indicated in the bottom left panel. Colors indicate the classification of the antibiotics tested (bottom right).
(PDF)

S4 Fig Role of *metE* in *M*. *smegmatis* SULFA resistance tested on rich media. **(A**) SULFA susceptibility tested by 10X serial dilution on NE medium. Cultures growing at OD1 were 10X serially diluted, and 5 μl cell suspensions were spotted onto NE without (-) or with (+) 10.5 μg/ml SCP. Growth was recorded after 5 days of incubation at 37°C. (**B**) SULFA susceptibility tested by disc diffusion on NE medium. Cells of *M*. *smegmatis* strains were seeded onto the surface of NE plates and paper discs embedded with 1 mg SCP were placed at the center. Susceptibility, visualized as the zone of inhibition surrounding the discs, was recorded after 5 days of incubation at 37°C. Neither deletion nor overexpression of *metE* altered *M*. *smegmatis* SULFA resistance. Similar experiments performed on LB agar were demonstrated in figures (**C**) and (**D**), respectively.
(PDF)

S5 Fig Role of *metE* in *M*. *smegmatis* SULFA resistance tested on a minimal medium. **(A**) SULFA susceptibility tested by 10X serial dilution on 7H10 medium. Cultures growing at OD1 were 10X serially diluted, and 5 μl cell suspensions were spotted onto 7H10 without (-) or with (+) 5 μg/ml SCP. Growth was recorded after 5 days of incubation at 37°C. (**B**) SULFA susceptibility tested by disc diffusion on 7H10 medium. Cells of *M*. *smegmatis* strains were seeded onto the surface of 7H10 plates and paper discs embedded with 1 mg SCP were placed at the center. Susceptibility, visualized as the zone of inhibition surrounding the discs, was recorded after 5 days of incubation at 37°C.
(PDF)

S6 Fig Morphological differences of *M*. *tuberculosis* strains. Cells of H37Rv, CDC1551, and the CDC1551 strain *in trans* expressing the *metH* gene from H37Rv (CDC1551/*metH*), were inoculated on the surface of a solid rich medium (NE-OADC, top) or a minimal medium (7H10-OADC, bottom). Morphology was recorded after 2 and 4 weeks of growth at 37°C. Colonies of CDC1551 resembled the *M*. *smegmatis* “white” mutants while *in trans* expression of the *metH* gene from H37Rv results in a morphology similar to H37Rv.
(PDF)

S7 Fig SULFA susceptibility of *S*. *typhimurium* strains. (**A**) SULFA susceptibility tested by 10X serial dilution. Cultures growing at OD1 were 10X serially diluted, and 5 μl cell suspensions were spotted on LB agar without (-) or with (+) 125 μg/ml SMZ. Growth was recorded after 48 h at 37°C. (**B**) Effects of methionine synthases and B_12_ related genes on the folate pool of *S*. *typhimurium* growing in the complex LB medium. Shown are cellular levels of methyl folate (top), non-methyl folate (middle) and total folate (bottom) in *S*. *typhimurium* strains treated with SMZ. Bars represent means of biological triplicates with standard deviations. ns, no significant difference compared to the parental strain.
(PDF)

S8 Fig Role of *metH* in *S*. *typhimurium* susceptibility to non-antifolates. A representative disc diffusion test shows that *metH* does not affect *S*. *typhimurium* resistance to non-antifolates. Cells of *metH*(+) (top left) and *metH*(-) (top right) were seeded onto the surface of LB agar. Antibiotic discs were applied at positions indicated in the bottom left panel. Bottom right panel indicates the antibiotics’ classification.
(PDF)

S9 Fig Additional characterization of the methylfolate trap in *S*. *typhimurium*. (**A**) SULFA uptake by *S*. *typhimurium* strains. Cultures of *metH*(+) (red) and *metH*(-) (blue) were grown to OD1 when 1 μCi/ml [^3^H]-SMZ was added. At selected time points, samples were collected and cells were filtered and washed. Incorporated radioactivity was measured by liquid scintillation counting. Bars represent means of biological triplicates with standard deviations. ns, no significant difference. (**B-D**) Synthesis of DNA, RNA, and protein of *S*. *typhimurium metH*(+) (red) and *metH*(-) (blue) strains following SULFA treatment. 2.5 mg/ml SMZ was added when cultures reached OD1. At selected time points post-SMZ treatment, samples from each strain were collected and treated with 10 μCi/ml [^3^H]-thymidine (B), 10 μCi/ml [^3^H]-uracil (C), or 8 μCi/ml [^35^S]-methionine (D), respectively, for 20 min at 37°C. Following treatment with 1 M NaOH for 30 min at 50°C, macromolecules were precipitated with cold TCA, filtered onto Whatman glass microfibers, and washed. Incorporated radioactivity was measured by liquid scintillation counting. Bars represent means of biological triplicates with standard deviations. *, significant differences in RNA synthesis between *metH*(+) and *metH*(-), p<0.05; ns, no significant difference. (**E**) Diagram depicting the interaction of one-carbon metabolism and the methionine-homocysteine cycle. When the reaction catalyzed by B_12_-dependent methionine synthase fails, the methylfolate trap occurs, resulting in the accumulation of not only 5-CH_3_-H_4_PteGlun but also SAM and SAH. Besides the sulfate assimilation pathway, bacteria can convert SAH to Hcy, either directly or through the formation of *S*-ribosylhomocysteine (SRH). Hcy is further converted to Hcy-thiolactone (HTL), which interacts with selected proteins thus affecting their functions. (**F**) Extracellular accumulation of Hcy-thiolactone (HTL, μM) in *metH*(-) cultures (blue) compared to *metH*(+) (red) during growth in the presence of SULFAs. Cultures were collected following the addition of 2.5 mg/ml SMZ and cells were removed by centrifugation. Samples were separated by HPLC with fluorescence detection. Bars represent means of biological triplicates with standard deviations. Bars represent means of biological triplicates with standard deviations. ** p < 0.01; *** p< 0.001; ns, no significant difference.
(PDF)

S10 Fig Dynamics of individual folate species in *S*. *typhimurium* strains. Dynamics of individual folate species in *S*. *typhimurium metH*(+) (top) and *metH*(-) (bottom) cells following SULFA treatment. At selected time points following the addition of 2.5 mg/ml SMZ, cells were collected, and folate was extracted and analyzed by LC-MS/MS.
(PDF)

S11 Fig Effect of EtPhCbl on *in vitro* SULFA susceptibility. SULFA susceptibility in *E*. *coli*, *S*. *typhimurium*, *P*. *aeruginosa*, and *M*. *smegmatis* was analyzed by 10X serial dilution. 5 μl cell suspensions were spotted onto LB agar in the absence or presence of 125 μg/ml SMZ, and varying concentrations of B_12_ or EtPhCbl (antivitamin B_12_). Growth was recorded after 48 h at 37°C.
(PDF)

S1 Table Whole-genome antifolate resistance determinants in *M*. *smegmatis*. (DOC)

S2 Table Strains used in this study. (DOC)

S3 Table Plasmids used in this study. (DOC)

S4 Table Oligonucleotides used in this study. (DOC)

S1 Text Additional methods used in this study. (DOC)

## References

[1] Domagk G. Ein Beitrag zur Chemotherapie der bakteriellen Infektionen. Deutsch Med Wschr. 1935;61(15):250–3.

[2] Lesch JE. The first miracle drugs: how the sulfa drugs transformed medicine New York: Oxford University Press; 2007 x, 364 p. p.

[3] Libecco JA, Powell KR. Trimethoprim/sulfamethoxazole: clinical update. Pediatr Rev. 2004;25(11):375–80. Epub 2004/11/03. 15520082

[4] Grunberg E, DeLorenzo WF. Potentiation of sulfonamides and antibiotics by trimethoprim [2,4-diamino-5-(3,4,5-trimethoxybenzyl) pyrimidine]. Antimicrob Agents Chemother. 1966;6:430–3. 5985268

[5] Levitz RE, Quintiliani R. Trimethoprim-sulfamethoxazole for bacterial meningitis. Annals of internal medicine. 1984;100(6):881–90. 6372565 10.7326/0003-4819-100-6-881

[6] Grim SA, Rapp RP, Martin CA, Evans ME. Trimethoprim-sulfamethoxazole as a viable treatment option for infections caused by methicillin-resistant *Staphylococcus aureus* . Pharmacotherapy. 2005;25(2):253–64. Epub 2005/03/16. 10.1592/phco.25.2.253.56956 15767239

[7] Bermingham A, Derrick JP. The folic acid biosynthesis pathway in bacteria: evaluation of potential for antibacterial drug discovery. Bioessays. 2002;24(7):637–48. Epub 2002/07/12. 10.1002/bies.10114 12111724

[8] Huang TS, Kunin CM, Yan BS, Chen YS, Lee SS, Syu W Jr. Susceptibility of *Mycobacterium tuberculosis* to sulfamethoxazole, trimethoprim and their combination over a 12 year period in Taiwan. J Antimicrob Chemother. 2012;67(3):633–7. Epub 2011/12/01. 10.1093/jac/dkr501 22127584

[9] Scholar E, Pratt W. The Antimicrobial Drugs 2nd ed. Oxford: Oxford University Press; 2000 xii, 607 p. p.

[10] Date AA, Vitoria M, Granich R, Banda M, Fox MY, Gilks C. Implementation of co-trimoxazole prophylaxis and isoniazid preventive therapy for people living with HIV. Bull World Health Organ. 2010;88(4):253–9. Epub 2010/05/01. PubMed Central PMCID: PMC2855598. 10.2471/BLT.09.066522 20431788 PMC2855598

[11] Wright GD. Resisting resistance: new chemical strategies for battling superbugs. Chem Biol. 2000;7(6):R127–32. Epub 2000/06/30. 10873842 10.1016/s1074-5521(00)00126-5

[12] Ogwang S, Nguyen HT, Sherman M, Bajaksouzian S, Jacobs MR, Boom WH, et al Bacterial conversion of folinic acid is required for antifolate resistance. J Biol Chem. 2011;286(17):15377–90. Epub 2011/03/05. PubMed Central PMCID: PMC3083218. 10.1074/jbc.M111.231076 21372133 PMC3083218

[13] Cohen SS. On the nature of thymineless death. Ann N Y Acad Sci. 1971;186:292–301. 4944291 10.1111/j.1749-6632.1971.tb31155.x

[14] Then R, Angehrn P. Sulphonamide-induced 'thymineless death' in Escherichia coli. J Gen Microbiol. 1973;76(2):255–63. 10.1099/00221287-76-2-255 4579126

[15] Kwon YK, Higgins MB, Rabinowitz JD. Antifolate-induced depletion of intracellular glycine and purines inhibits thymineless death in *E*. *coli* . ACS Chem Biol. 2010;5(8):787–95. PubMed Central PMCID: PMC2945287. 10.1021/cb100096f 20553049 PMC2945287

[16] Barner HD, Cohen SS. The induction of thymine synthesis by T2 infection of a thymine requiring mutant of *Escherichia coli* . J Bacteriol. 1954;68(1):80–8. PubMed Central PMCID: PMC357338. 13183905 10.1128/jb.68.1.80-88.1954 PMC357338

[17] Khodursky A, Guzman EC, Hanawalt PC. Thymineless Death Lives On: New Insights into a Classic Phenomenon. Annu Rev Microbiol. 2015;69:247–63. 10.1146/annurev-micro-092412-155749 26253395

[18] Carmel R, Jacobsen DW. Homocysteine in health and disease Cambridge; New York: Cambridge University Press; 2001 xvi, 510 p. p.

[19] Herbert V, Zalusky R. Interrelations of vitamin B_12_ and folic acid metabolism: folic acid clearance studies. J Clin Invest. 1962;41:1263–76. Epub 1962/06/01. PubMed Central PMCID: PMC291041. 10.1172/JCI104589 13906634 PMC291041

[20] Sauer H, Wilmanns W. Cobalamin dependent methionine synthesis and methyl-folate-trap in human vitamin B_12_ deficiency. Br J Haematol. 1977;36(2):189–98. Epub 1977/06/01. 871432 10.1111/j.1365-2141.1977.tb00639.x

[21] Nijhout HF, Reed MC, Budu P, Ulrich CM. A mathematical model of the folate cycle: new insights into folate homeostasis. J Biol Chem. 2004;279(53):55008–16. Epub 2004/10/22. 10.1074/jbc.M410818200 15496403

[22] Fujii K, Nagasaki T, Huennekens FM. Accumulation of 5-methyltetrahydrofolate in cobalamin-deficient L1210 mouse leukemia cells. J Biol Chem. 1982;257(5):2144–6. Epub 1982/03/10. 7061412

[23] Danishpajooh IO, Gudi T, Chen Y, Kharitonov VG, Sharma VS, Boss GR. Nitric oxide inhibits methionine synthase activity *in vivo* and disrupts carbon flow through the folate pathway. J Biol Chem. 2001;276(29):27296–303. Epub 2001/05/24. 10.1074/jbc.M104043200 11371572

[24] Nicolaou A, Kenyon SH, Gibbons JM, Ast T, Gibbons WA. *In vitro* inactivation of mammalian methionine synthase by nitric oxide. Eur J Clin Invest. 1996;26(2):167–70. Epub 1996/02/01. 8904527 10.1046/j.1365-2362.1996.122254.x

[25] Green JM, Ballou DP, Matthews RG. Examination of the role of methylenetetrahydrofolate reductase in incorporation of methyltetrahydrofolate into cellular metabolism. FASEB J. 1988;2(1):42–7. Epub 1988/01/01. 3335280 10.1096/fasebj.2.1.3335280

[26] Matthews RG, Daubner SC. Modulation of methylenetetrahydrofolate reductase activity by *S*-adenosylmethionine and by dihydrofolate and its polyglutamate analogues. Adv Enzyme Regul. 1982;20:123–31. Epub 1982/01/01. 7051769 10.1016/0065-2571(82)90012-7

[27] Swanson DA, Liu ML, Baker PJ, Garrett L, Stitzel M, Wu J, et al Targeted disruption of the methionine synthase gene in mice. Mol Cell Biol. 2001;21(4):1058–65. PubMed Central PMCID: PMC99560. 10.1128/MCB.21.4.1058-1065.2001 11158293 PMC99560

[28] Wolff KA, Nguyen HT, Cartabuke RH, Singh A, Ogwang S, Nguyen L. Protein kinase G is required for intrinsic antibiotic resistance in mycobacteria. Antimicrob Agents Chemother. 2009;53(8):3515–9. Epub 2009/06/17. PubMed Central PMCID: PMC2715596. 10.1128/AAC.00012-09 19528288 PMC2715596

[29] Buchmeier NA, Newton GL, Koledin T, Fahey RC. Association of mycothiol with protection of *Mycobacterium tuberculosis* from toxic oxidants and antibiotics. Mol Microbiol. 2003;47(6):1723–32. 12622824 10.1046/j.1365-2958.2003.03416.x

[30] Xu X, Vilcheze C, Av-Gay Y, Gomez-Velasco A, Jacobs WR Jr. Precise null deletion mutations of the mycothiol synthesis genes reveal their role in isoniazid and ethionamide resistance in *Mycobacterium smegmatis* . Antimicrob Agents Chemother. 2011;55(7):3133–9. Epub 2011/04/20. PubMed Central PMCID: PMC3122461. 10.1128/AAC.00020-11 21502624 PMC3122461

[31] Duval BD, Mathew A, Satola SW, Shafer WM. Altered growth, pigmentation, and antimicrobial susceptibility properties of *Staphylococcus aureus* due to loss of the major cold shock gene *cspB* . Antimicrob Agents Chemother. 2010;54(6):2283–90. Epub 2010/04/07. PubMed Central PMCID: PMC2876397. 10.1128/AAC.01786-09 20368405 PMC2876397

[32] Nguyen L, Chinnapapagari S, Thompson CJ. FbpA-Dependent biosynthesis of trehalose dimycolate is required for the intrinsic multidrug resistance, cell wall structure, and colonial morphology of *Mycobacterium smegmatis* . J Bacteriol. 2005;187(19):6603–11. 10.1128/JB.187.19.6603-6611.2005 16166521 PMC1251576

[33] Gebhardt H, Meniche X, Tropis M, Kramer R, Daffe M, Morbach S. The key role of the mycolic acid content in the functionality of the cell wall permeability barrier in Corynebacterineae. Microbiology. 2007;153(Pt 5):1424–34. Epub 2007/04/28. 10.1099/mic.0.2006/003541-0 17464056

[34] van Kessel JC, Hatfull GF. Recombineering in *Mycobacterium tuberculosis* . Nat Methods. 2007;4(2):147–52. 10.1038/nmeth996 17179933

[35] Pejchal R, Ludwig ML. Cobalamin-independent methionine synthase (MetE): a face-to-face double barrel that evolved by gene duplication. PLoS Biol. 2005;3(2):e31 Epub 2005/01/05. PubMed Central PMCID: PMC539065. 10.1371/journal.pbio.0030031 15630480 PMC539065

[36] Savvi S, Warner DF, Kana BD, McKinney JD, Mizrahi V, Dawes SS. Functional characterization of a vitamin B12-dependent methylmalonyl pathway in *Mycobacterium tuberculosis*: implications for propionate metabolism during growth on fatty acids. J Bacteriol. 2008;190(11):3886–95. PubMed Central PMCID: PMCPMC2395058. 10.1128/JB.01767-07 18375549 PMC2395058

[37] Gopinath K, Venclovas C, Ioerger TR, Sacchettini JC, McKinney JD, Mizrahi V, et al A vitamin B12 transporter in *Mycobacterium tuberculosis* . Open biology. 2013;3(2):120175 PubMed Central PMCID: PMC3603451. 10.1098/rsob.120175 23407640 PMC3603451

[38] Warner DF, Savvi S, Mizrahi V, Dawes SS. A riboswitch regulates expression of the coenzyme B_12_-independent methionine synthase in *Mycobacterium tuberculosis*: implications for differential methionine synthase function in strains H37Rv and CDC1551. J Bacteriol. 2007;189(9):3655–9. Epub 2007/02/20. PubMed Central PMCID: PMC1855906. 10.1128/JB.00040-07 17307844 PMC1855906

[39] Valway SE, Sanchez MP, Shinnick TF, Orme I, Agerton T, Hoy D, et al An outbreak involving extensive transmission of a virulent strain of *Mycobacterium tuberculosis* . N Engl J Med. 1998;338(10):633–9. Epub 1998/03/05. 10.1056/NEJM199803053381001 9486991

[40] Fleischmann RD, Alland D, Eisen JA, Carpenter L, White O, Peterson J, et al Whole-genome comparison of *Mycobacterium tuberculosis* clinical and laboratory strains. J Bacteriol. 2002;184(19):5479–90. Epub 2002/09/10. PubMed Central PMCID: PMC135346. 10.1128/JB.184.19.5479-5490.2002 12218036 PMC135346

[41] Munoz-Elias EJ, Upton AM, Cherian J, McKinney JD. Role of the methylcitrate cycle in *Mycobacterium tuberculosis* metabolism, intracellular growth, and virulence. Mol Microbiol. 2006;60(5):1109–22. 10.1111/j.1365-2958.2006.05155.x 16689789

[42] Vilcheze C, Jacobs WR Jr. The combination of sulfamethoxazole, trimethoprim, and isoniazid or rifampin is bactericidal and prevents the emergence of drug resistance in *Mycobacterium tuberculosis* . Antimicrob Agents Chemother. 2012;56(10):5142–8. Epub 2012/07/25. 10.1128/AAC.00832-12 22825115 PMC3457372

[43] Raux E, Lanois A, Levillayer F, Warren MJ, Brody E, Rambach A, et al *Salmonella typhimurium* cobalamin (vitamin B_12_) biosynthetic genes: functional studies in *S*. *typhimurium* and *Escherichia coli* . J Bacteriol. 1996;178(3):753–67. Epub 1996/02/01. PubMed Central PMCID: PMC177722. 8550510 10.1128/jb.178.3.753-767.1996 PMC177722

[44] Sekowska A, Kung HF, Danchin A. Sulfur metabolism in *Escherichia coli* and related bacteria: facts and fiction. J Mol Microbiol Biotechnol. 2000;2(2):145–77. Epub 2000/08/12. 10939241

[45] Cadieux N, Bradbeer C, Reeger-Schneider E, Koster W, Mohanty AK, Wiener MC, et al Identification of the periplasmic cobalamin-binding protein BtuF of *Escherichia coli* . J Bacteriol. 2002;184(3):706–17. Epub 2002/01/16. PubMed Central PMCID: PMC139523. 10.1128/JB.184.3.706-717.2002 11790740 PMC139523

[46] Jacobs MA, Alwood A, Thaipisuttikul I, Spencer D, Haugen E, Ernst S, et al Comprehensive transposon mutant library of *Pseudomonas aeruginosa* . Proc Natl Acad Sci U S A. 2003;100(24):14339–44. Epub 2003/11/18. PubMed Central PMCID: PMC283593. 10.1073/pnas.2036282100 14617778 PMC283593

[47] Jakubowski H. Proofreading *in vivo*: editing of homocysteine by methionyl-tRNA synthetase in *Escherichia coli* . Proc Natl Acad Sci U S A. 1990;87(12):4504–8. PubMed Central PMCID: PMC54144. 2191291 10.1073/pnas.87.12.4504 PMC54144

[48] Jakubowski H. Homocysteine in Protein Structure/Function and Human Disease Chemical Biology of Homocysteine-containing Proteins Wien-Heidelberg-New York-Dordrecht-London: Springer; 2013.

[49] Jakubowski H. The determination of homocysteine-thiolactone in biological samples. Anal Biochem. 2002;308(1):112–9. 12234471 10.1016/s0003-2697(02)00224-5

[50] Morris RP, Nguyen L, Gatfield J, Visconti K, Nguyen K, Schnappinger D, et al Ancestral antibiotic resistance in *Mycobacterium tuberculosis* . Proc Natl Acad Sci U S A. 2005;102(34):12200–5. 10.1073/pnas.0505446102 16103351 PMC1186028

[51] Lerner-Ellis JP, Tirone JC, Pawelek PD, Dore C, Atkinson JL, Watkins D, et al Identification of the gene responsible for methylmalonic aciduria and homocystinuria, *cblC* type. Nat Genet. 2006;38(1):93–100. 10.1038/ng1683 16311595

[52] Fowler CC, Brown ED, Li Y. Using a riboswitch sensor to examine coenzyme B(12) metabolism and transport in *E*. *coli* . Chem Biol. 2010;17(7):756–65. 10.1016/j.chembiol.2010.05.025 20659688

[53] Ruetz M, Gherasim C, Gruber K, Fedosov S, Banerjee R, Krautler B. Access to organometallic arylcobaltcorrins through radical synthesis: 4-ethylphenylcobalamin, a potential "antivitamin B(12)". Angewandte Chemie. 2013;52(9):2606–10. PubMed Central PMCID: PMC3843227. 10.1002/anie.201209651 23404623 PMC3843227

[54] Mutti E, Ruetz M, Birn H, Krautler B, Nexo E. 4-ethylphenyl-cobalamin impairs tissue uptake of vitamin B_12_ and causes vitamin B_12_ deficiency in mice. PLoS One. 2013;8(9):e75312 PubMed Central PMCID: PMC3779197. 10.1371/journal.pone.0075312 24073261 PMC3779197

[55] Kim J, Gherasim C, Banerjee R. Decyanation of vitamin B_12_ by a trafficking chaperone. Proc Natl Acad Sci U S A. 2008;105(38):14551–4. PubMed Central PMCID: PMC2567227. 10.1073/pnas.0805989105 18779575 PMC2567227

[56] Hannibal L, Kim J, Brasch NE, Wang S, Rosenblatt DS, Banerjee R, et al Processing of alkylcobalamins in mammalian cells: A role for the MMACHC (*cblC*) gene product. Mol Genet Metab. 2009;97(4):260–6. PubMed Central PMCID: PMC2709701. 10.1016/j.ymgme.2009.04.005 19447654 PMC2709701

[57] Wolff KA, Nguyen L. Strategies for potentiation of ethionamide and folate antagonists against *Mycobacterium tuberculosis* . Expert Rev Anti Infect Ther. 2012;10(9):971–81. 10.1586/eri.12.87 23106273 PMC3971469

[58] Nguyen L. Targeting antibiotic resistance mechanisms in *Mycobacterium tuberculosis*: recharging the old magic bullets. Expert Rev Anti Infect Ther. 2012;10(9):963–5. Epub 2012/10/31. 10.1586/eri.12.85 23106271 PMC3970175

[59] Hoffbrand AV, Jackson BF. Correction of the DNA synthesis defect in vitamin B_12_ deficiency by tetrahydrofolate: evidence in favour of the methyl-folate trap hypothesis as the cause of megaloblastic anaemia in vitamin B_12_ deficiency. Br J Haematol. 1993;83(4):643–7. Epub 1993/04/01. 8518179 10.1111/j.1365-2141.1993.tb04704.x

[60] Dierkes J, Domrose U, Ambrosch A, Schneede J, Guttormsen AB, Neumann KH, et al Supplementation with vitamin B_12_ decreases homocysteine and methylmalonic acid but also serum folate in patients with end-stage renal disease. Metabolism. 1999;48(5):631–5. Epub 1999/05/25. 10337865 10.1016/s0026-0495(99)90062-8

[61] Scott JM. Folate-vitamin B_12_ interrelationships in the central nervous system. Proc Nutr Soc. 1992;51(2):219–24. Epub 1992/08/01. 1438330 10.1079/pns19920032

[62] Wilson A, Leclerc D, Saberi F, Campeau E, Hwang HY, Shane B, et al Functionally null mutations in patients with the cblG-variant form of methionine synthase deficiency. American journal of human genetics. 1998;63(2):409–14. PubMed Central PMCID: PMC1377317. 10.1086/301976 9683607 PMC1377317

[63] Shane B, Stokstad EL. Vitamin B_12_-folate interrelationships. Annu Rev Nutr. 1985;5:115–41. Epub 1985/01/01. 10.1146/annurev.nu.05.070185.000555 3927946

[64] Aherne GW, Brown S. The Role of Uracil Misincorporation in Thymineless Death In: Jackman AL, editor. Cancer Drug Discovery and Development. Antifolate Drugs in Cancer Therapy. Totowa, NJ: Humana Press: Imprint: Humana Press,; 1999 p. 409–21.

[65] Luzzati D. Effect of thymine starvation on messenger ribonucleic acid synthesis in *Escherichia coli* . J Bacteriol. 1966;92(5):1435–46.; PubMed Central PMCID: PMC276442. 5332402 10.1128/jb.92.5.1435-1446.1966 PMC276442

[66] Ahmad SI, Kirk SH, Eisenstark A. Thymine metabolism and thymineless death in prokaryotes and eukaryotes. Annu Rev Microbiol. 1998;52:591–625. 10.1146/annurev.micro.52.1.591 9891809

[67] Whitfield CD, Steers EJ Jr., Weissbach H. Purification and properties of 5-methyltetrahydropteroyltriglutamate-homocysteine transmethylase. J Biol Chem. 1970;245(2):390–401. 4904482

[68] Gonzalez JC, Banerjee RV, Huang S, Sumner JS, Matthews RG. Comparison of cobalamin-independent and cobalamin-dependent methionine synthases from *Escherichia coli*: two solutions to the same chemical problem. Biochemistry. 1992;31(26):6045–56. Epub 1992/07/07. 1339288 10.1021/bi00141a013

