# Supplementary figures and images for: Methylfolate Trap Promotes Bacterial Thymineless Death by Sulfa Drugs
Source: PLoS Pathog. 2016 Oct 19;12(10):e1005949. doi: 10.1371/journal.ppat.1005949 (PMC5070874; doi:10.1371/journal.ppat.1005949)

Figure S1

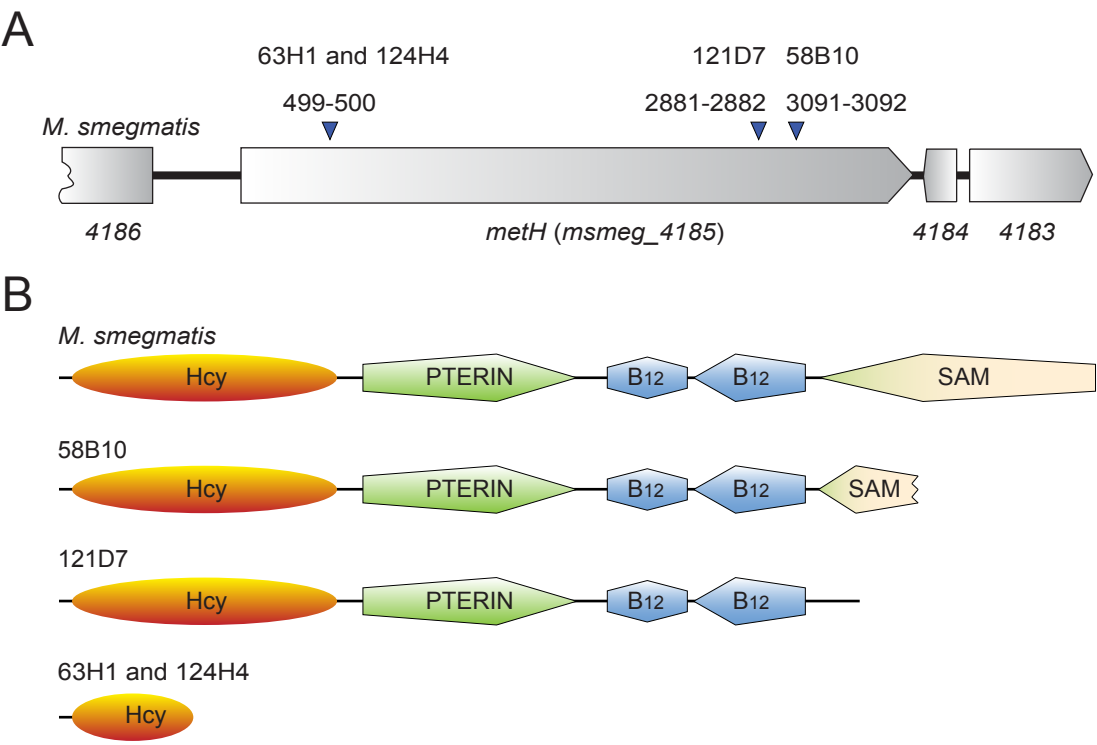

Supplement: S1 Fig — (A) Himar1 insertion into the metH (msmeg_4185) gene in 124H4, 63H1, 121D7 and 58B10. Arrows indicate the positions of the TA dinucleotides where Himar1 inserted. (B) Domain alignment of MetH truncation mutants compared to wild type using PROSITE (http://prosite.expasy.org). The truncated proteins in 58B10 and 121D7 are similar to that of CDC1551 shown Fig 3C. (PDF) [file ppat.1005949.s001.pdf]

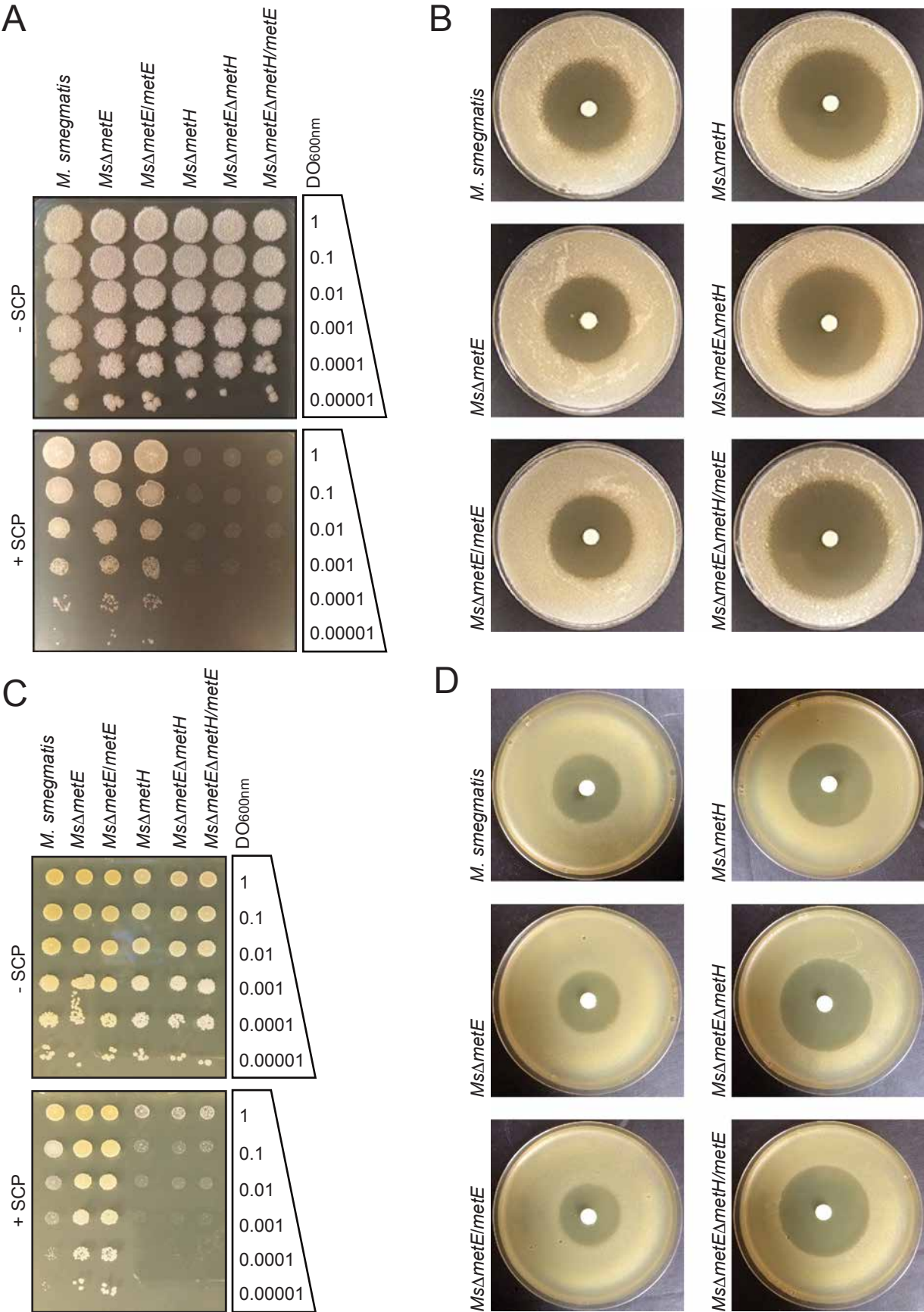

Supplement: S4 Fig — (A) SULFA susceptibility tested by 10X serial dilution on NE medium. Cultures growing at OD1 were 10X serially diluted, and 5 μl cell suspensions were spotted onto NE without (-) or with (+) 10.5 μg/ml SCP. Growth was recorded after 5 days of incubation at 37°C. (B) SULFA susceptibility tested by disc diffusion on NE medium. Cells of M. smegmatis strains were seeded onto the surface of NE plates and paper discs embedded with 1 mg SCP were placed at the center. Susceptibility, visualized as the zone of inhibition surrounding the discs, was recorded after 5 days of incubation at 37°C. Neither deletion nor overexpression of metE altered M. smegmatis SULFA resistance. Similar experiments performed on LB agar were demonstrated in figures (C) and (D), respectively. (PDF) [file ppat.1005949.s004.pdf]

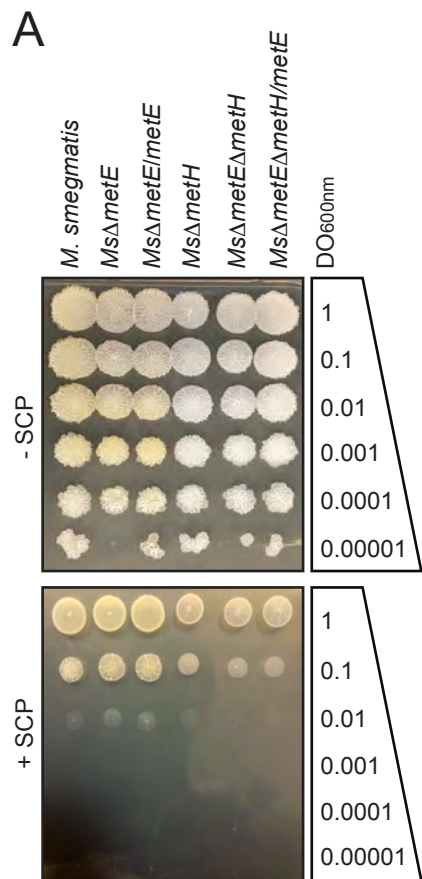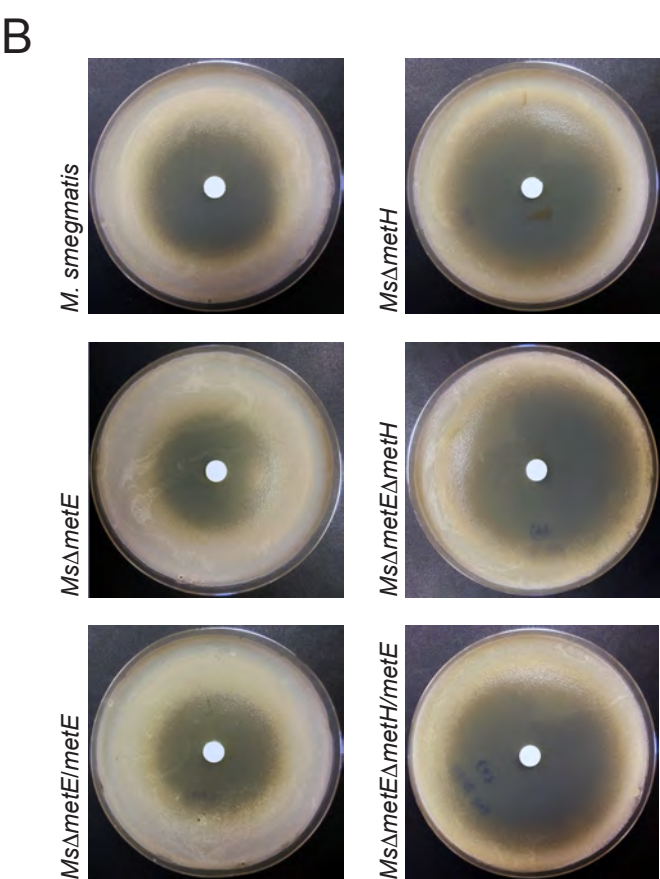

Supplement: S5 Fig — (A) SULFA susceptibility tested by 10X serial dilution on 7H10 medium. Cultures growing at OD1 were 10X serially diluted, and 5 μl cell suspensions were spotted onto 7H10 without (-) or with (+) 5 μg/ml SCP. Growth was recorded after 5 days of incubation at 37°C. (B) SULFA susceptibility tested by disc diffusion on 7H10 medium. Cells of M. smegmatis strains were seeded onto the surface of 7H10 plates and paper discs embedded with 1 mg SCP were placed at the center. Susceptibility, visualized as the zone of inhibition surrounding the discs, was recorded after 5 days of incubation at 37°C. (PDF) [file ppat.1005949.s005.pdf]

Figure S6

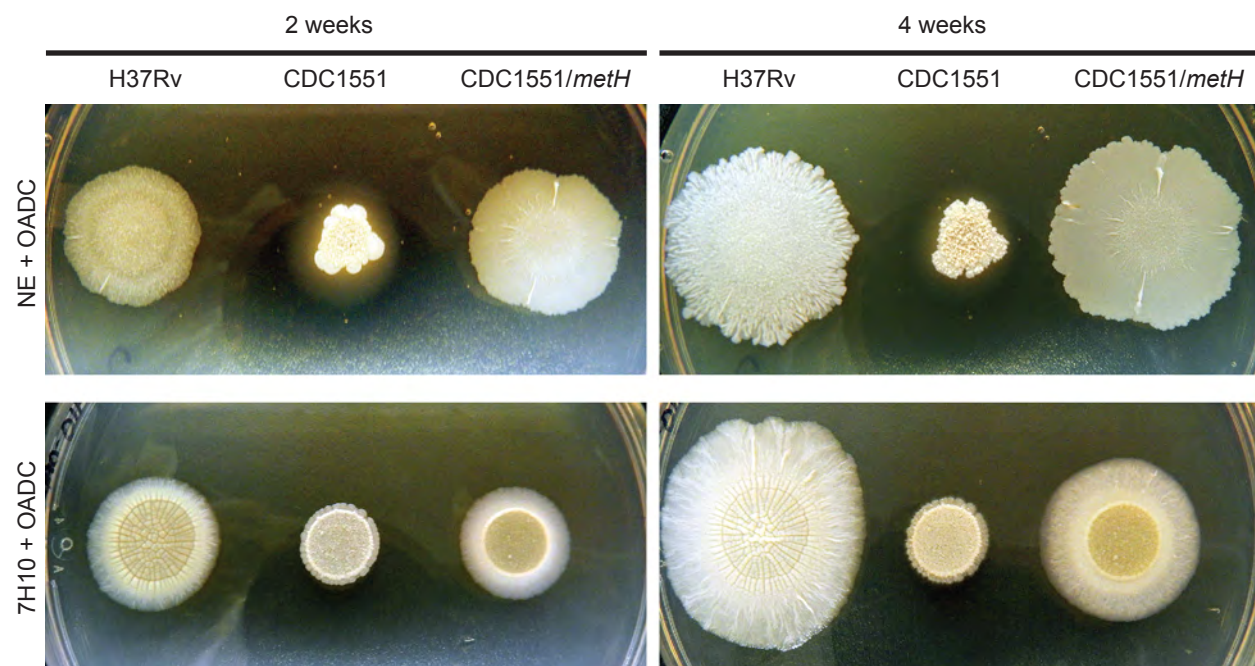

Supplement: S6 Fig — Cells of H37Rv, CDC1551, and the CDC1551 strain in trans expressing the metH gene from H37Rv (CDC1551/metH), were inoculated on the surface of a solid rich medium (NE-OADC, top) or a minimal medium (7H10-OADC, bottom). Morphology was recorded after 2 and 4 weeks of growth at 37°C. Colonies of CDC1551 resembled the M. smegmatis “white” mutants while in trans expression of the metH gene from H37Rv results in a morphology similar to H37Rv. (PDF) [file ppat.1005949.s006.pdf]

Figure S7

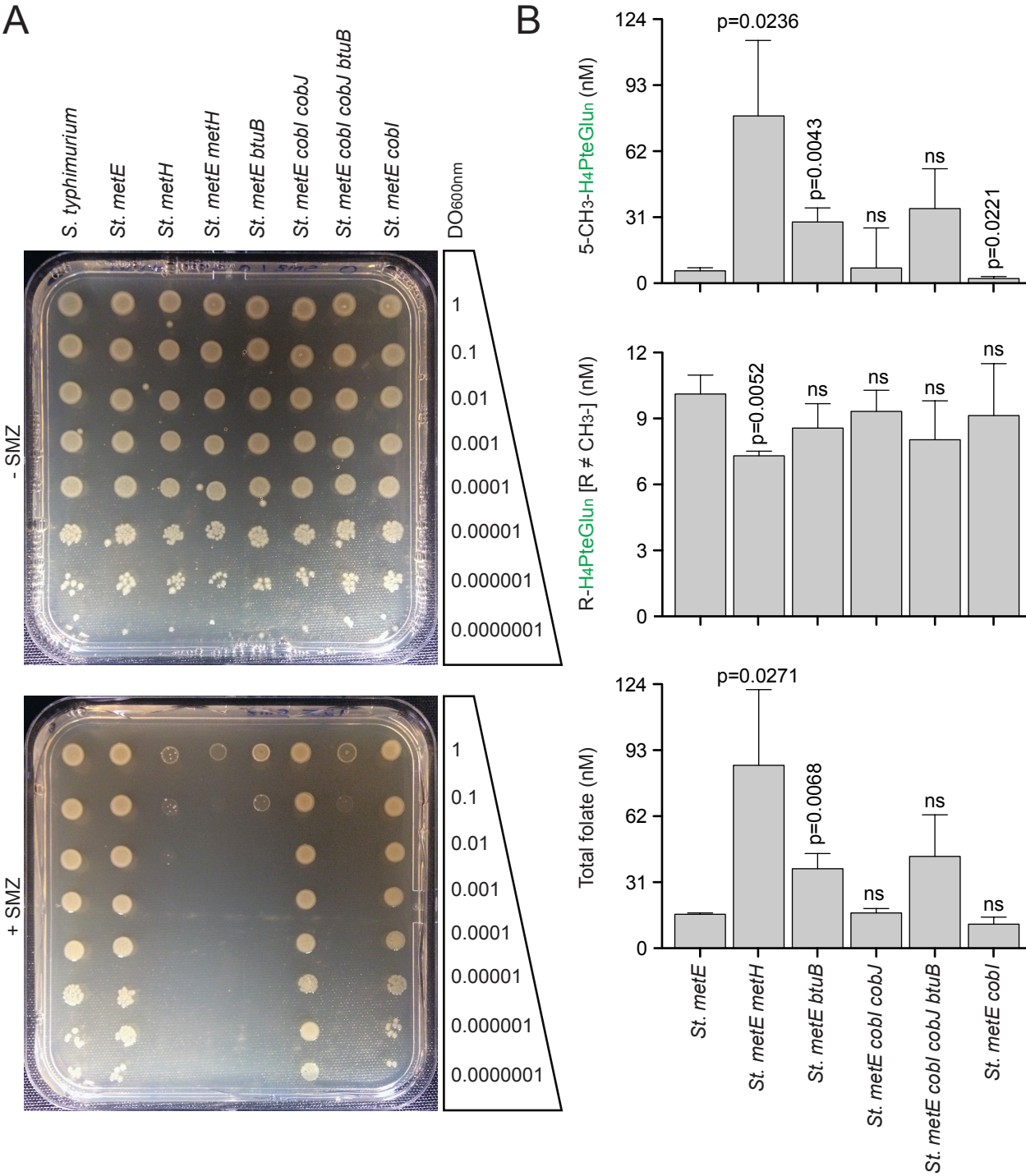

Supplement: S7 Fig — (A) SULFA susceptibility tested by 10X serial dilution. Cultures growing at OD1 were 10X serially diluted, and 5 μl cell suspensions were spotted on LB agar without (-) or with (+) 125 μg/ml SMZ. Growth was recorded after 48 h at 37°C. (B) Effects of methionine synthases and B12 related genes on the folate pool of S. typhimurium growing in the complex LB medium. Shown are cellular levels of methyl folate (top), non-methyl folate (middle) and total folate (bottom) in S. typhimurium strains treated with SMZ. Bars represent means of biological triplicates with standard deviations. ns, no significant difference compared to the parental strain. (PDF) [file ppat.1005949.s007.pdf]

Figure S9

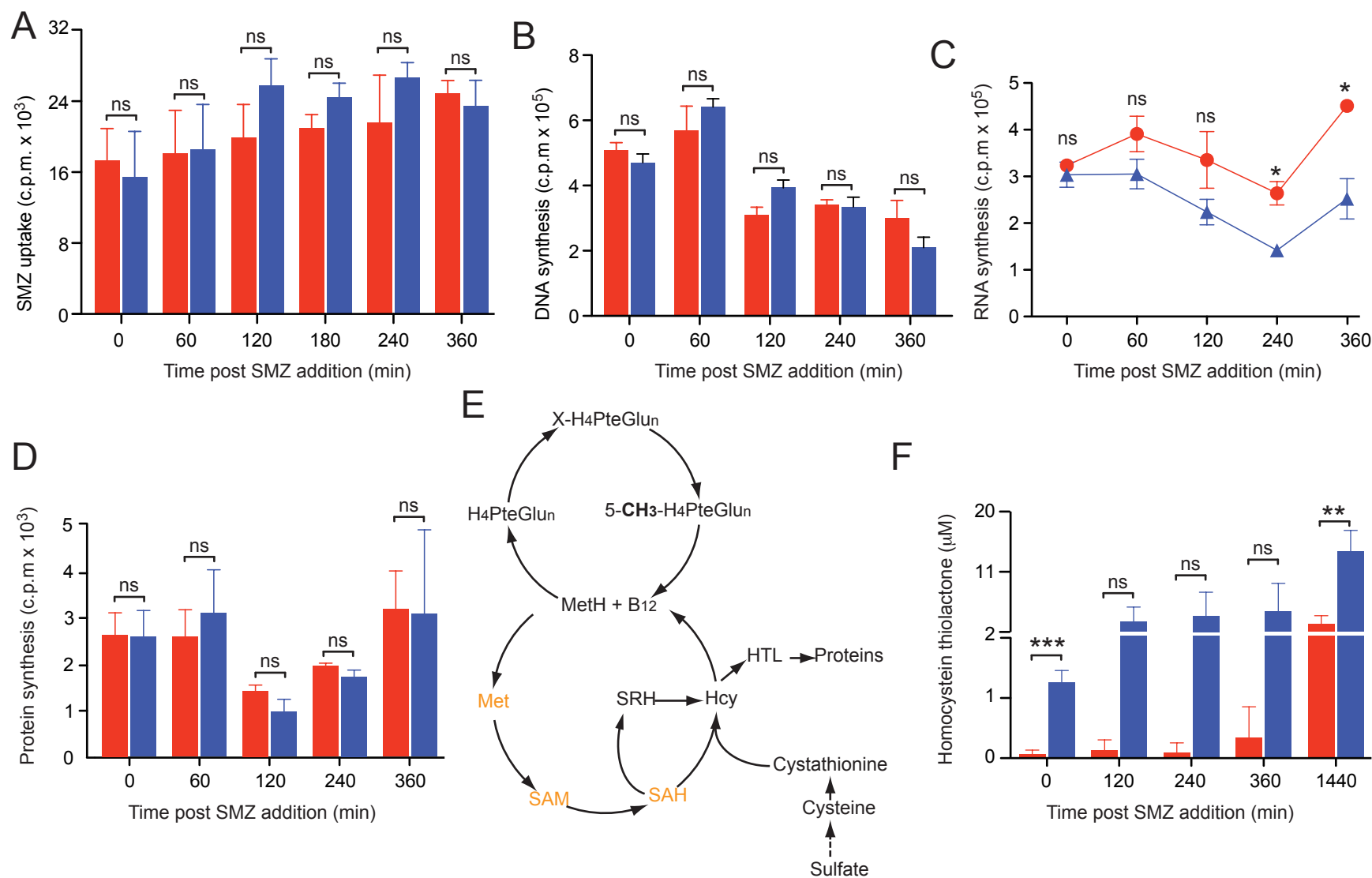

Supplement: S9 Fig — (A) SULFA uptake by S. typhimurium strains. Cultures of metH(+) (red) and metH(-) (blue) were grown to OD1 when 1 μCi/ml [3H]-SMZ was added. At selected time points, samples were collected and cells were filtered and washed. Incorporated radioactivity was measured by liquid scintillation counting. Bars represent means of biological triplicates with standard deviations. ns, no significant difference. (B-D) Synthesis of DNA, RNA, and protein of S. typhimurium metH(+) (red) and metH(-) (blue) strains following SULFA treatment. 2.5 mg/ml SMZ was added when cultures reached OD1. At selected time points post-SMZ treatment, samples from each strain were collected and treated with 10 μCi/ml [3H]-thymidine (B), 10 μCi/ml [3H]-uracil (C), or 8 μCi/ml [35S]-methionine (D), respectively, for 20 min at 37°C. Following treatment with 1 M NaOH for 30 min at 50°C, macromolecules were precipitated with cold TCA, filtered onto Whatman glass microfibers, and washed. Incorporated radioactivity was measured by liquid scintillation counting. Bars represent means of biological triplicates with standard deviations. *, significant differences in RNA synthesis between metH(+) and metH(-), p<0.05; ns, no significant difference. (E) Diagram depicting the interaction of one-carbon metabolism and the methionine-homocysteine cycle. When the reaction catalyzed by B12-dependent methionine synthase fails, the methylfolate trap occurs, resulting in the accumulation of not only 5-CH3-H4PteGlun but also SAM and SAH. Besides the sulfate assimilation pathway, bacteria can convert SAH to Hcy, either directly or through the formation of S-ribosylhomocysteine (SRH). Hcy is further converted to Hcy-thiolactone (HTL), which interacts with selected proteins thus affecting their functions. (F) Extracellular accumulation of Hcy-thiolactone (HTL, μM) in metH(-) cultures (blue) compared to metH(+) (red) during growth in the presence of SULFAs. Cultures were collected following the addition of 2.5 mg/ml SMZ and cel [file ppat.1005949.s009.pdf]

Figure S10

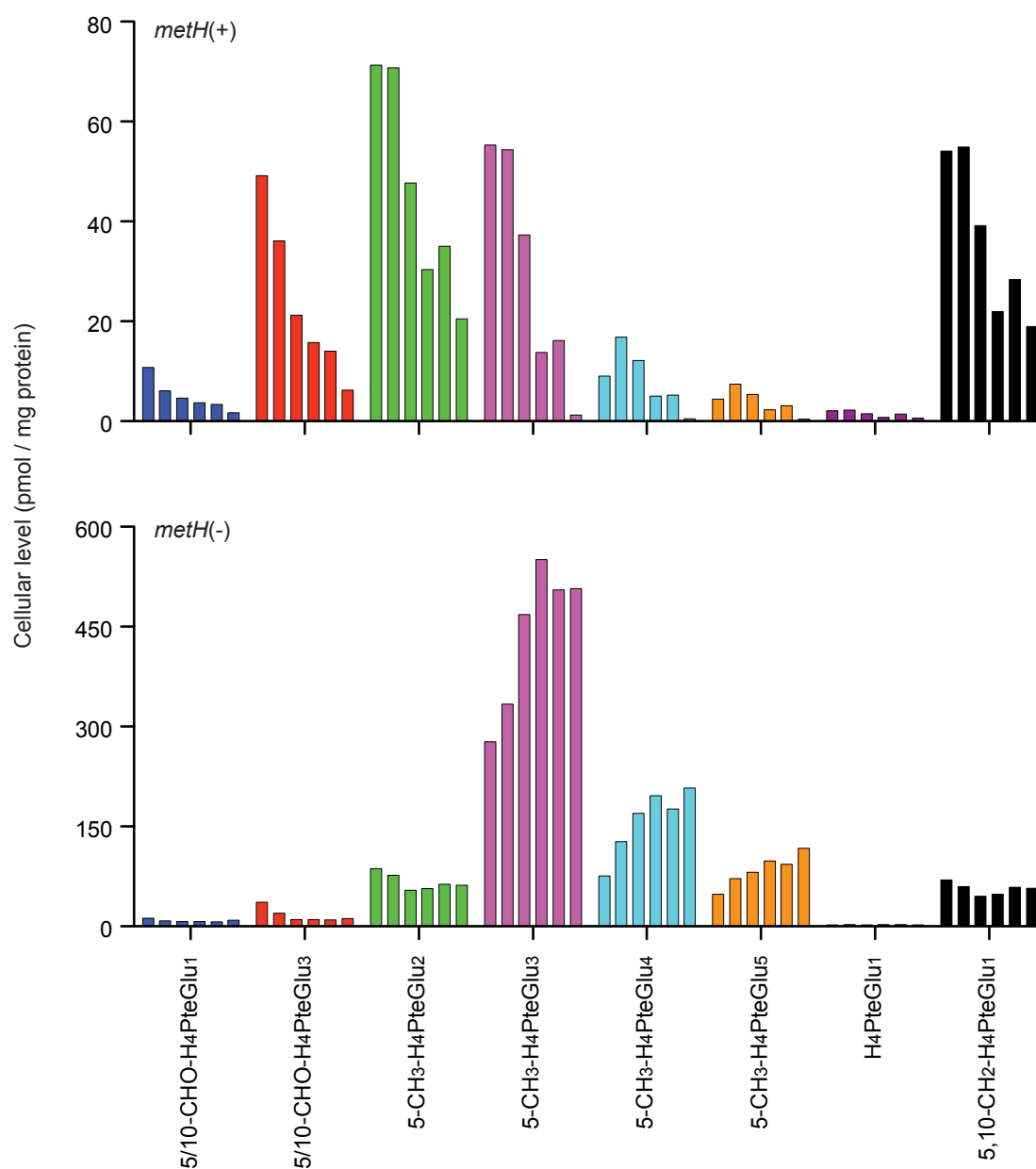

Supplement: S10 Fig — Dynamics of individual folate species in S. typhimurium metH(+) (top) and metH(-) (bottom) cells following SULFA treatment. At selected time points following the addition of 2.5 mg/ml SMZ, cells were collected, and folate was extracted and analyzed by LC-MS/MS. (PDF) [file ppat.1005949.s010.pdf]

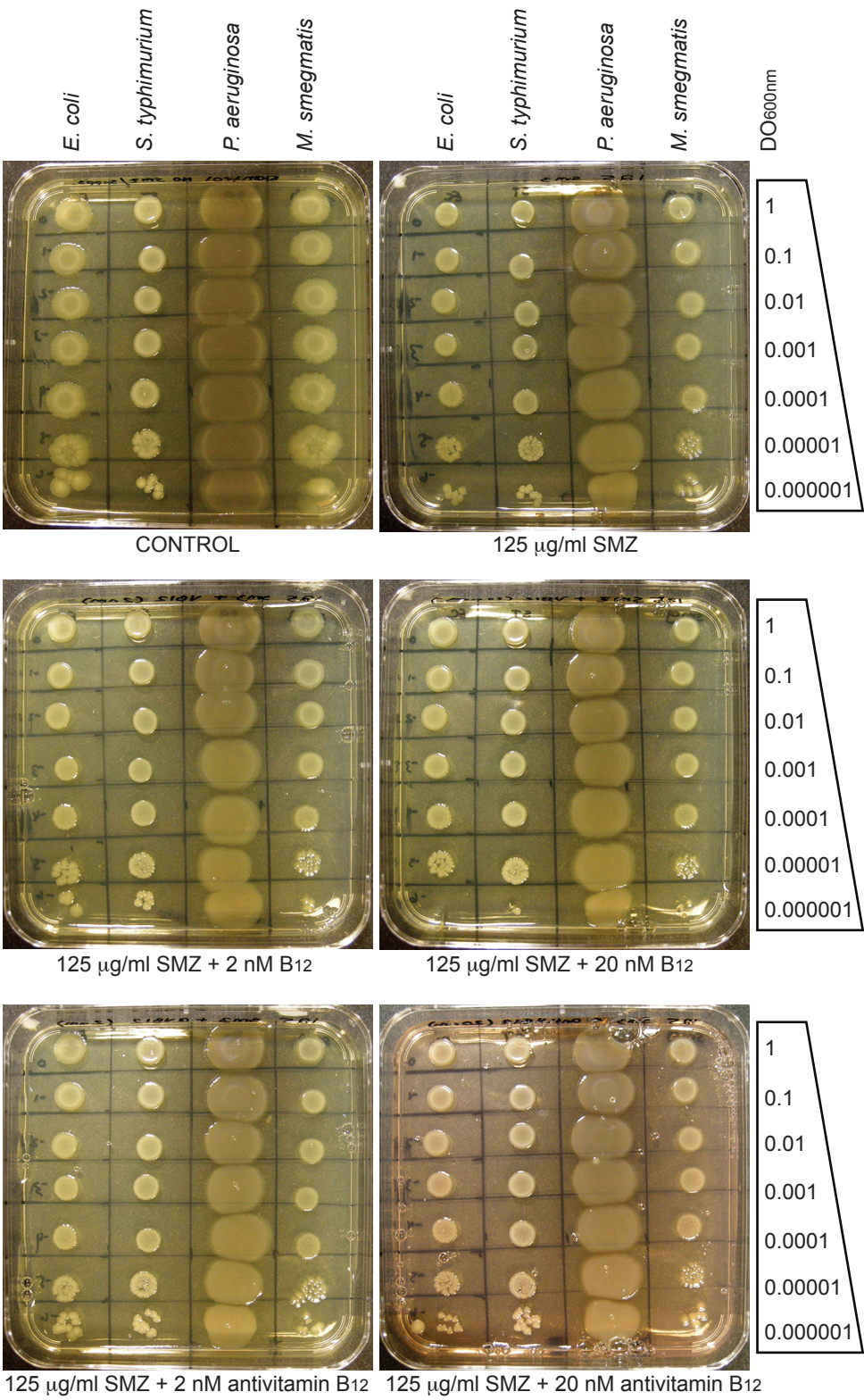

Supplement: S11 Fig — SULFA susceptibility in E. coli, S. typhimurium, P. aeruginosa, and M. smegmatis was analyzed by 10X serial dilution. 5 μl cell suspensions were spotted onto LB agar in the absence or presence of 125 μg/ml SMZ, and varying concentrations of B12 or EtPhCbl (antivitamin B12). Growth was recorded after 48 h at 37°C. (PDF) [file ppat.1005949.s011.pdf]
