# Supplementary material for: Methylfolate Trap Promotes Bacterial Thymineless Death by Sulfa Drugs
Source: PLoS Pathog. 2016 Oct 19;12(10):e1005949. doi: 10.1371/journal.ppat.1005949 (PMC5070874; doi:10.1371/journal.ppat.1005949)
Supplement: S2 Fig — A representative disc diffusion test shows the effect of metH deletion on M. smegmatis SULFA resistance. Cells of wild type (top left), MsΔmetH (top right), and complemented strain (bottom right) were seeded onto the surface of NE medium. Discs containing SULFA drugs classified in different subgroups were applied at the positions indicated in the bottom left panel. Colors indicate the groups to which the antibiotics belong. Non-SULFA antifolates were included as controls. (PDF) [file ppat.1005949.s002.pdf]

Figure S2

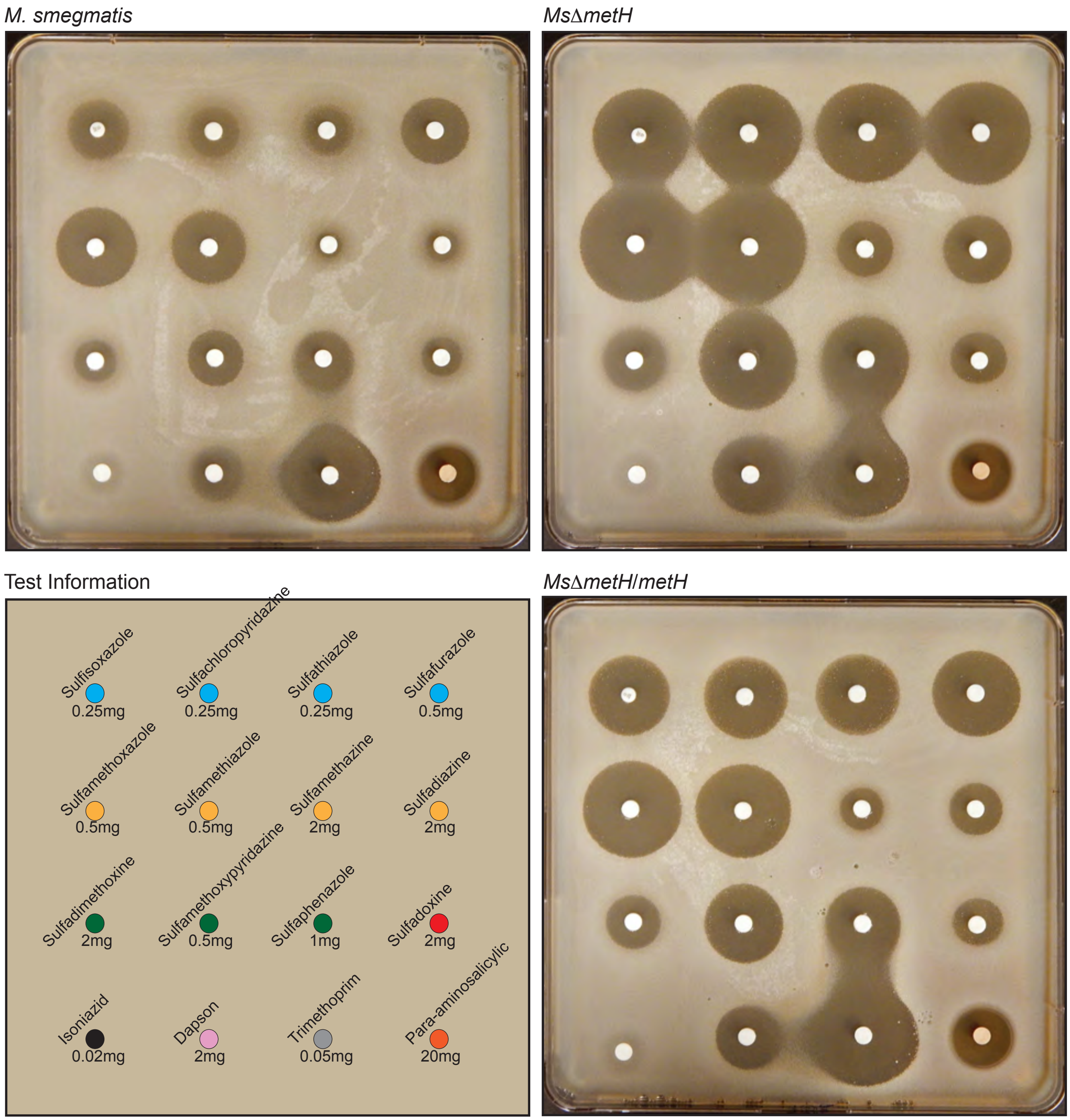

Short-acting Sulfa; Intermediate-acting Sulfa; Long-acting Sulfa; Ultra long-acting Sulfa

TB drug, potential folate inhibitor; Sulfone; DHFR inhibitor; TB drug, folate inhibitor
