# Supplementary material for: Methylfolate Trap Promotes Bacterial Thymineless Death by Sulfa Drugs
Source: PLoS Pathog. 2016 Oct 19;12(10):e1005949. doi: 10.1371/journal.ppat.1005949 (PMC5070874; doi:10.1371/journal.ppat.1005949)
Supplement: S3 Fig — A representative disc diffusion test shows that metH is not involved in M. smegmatis resistance to non-antifolate drugs. Cells of wild type (top left) and MsΔmetH (top right) were seeded onto the surface of NE. Antibiotic discs were applied at the positions indicated in the bottom left panel. Colors indicate the classification of the antibiotics tested (bottom right). (PDF) [file ppat.1005949.s003.pdf]

Figure S3

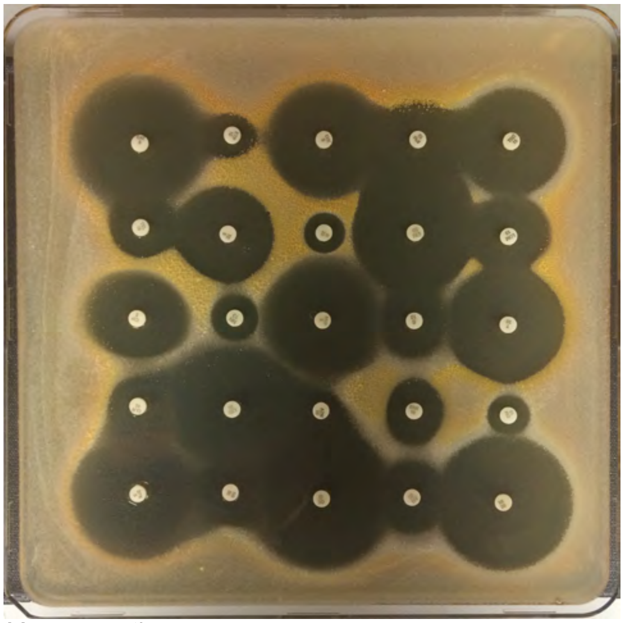

M. smegmatis

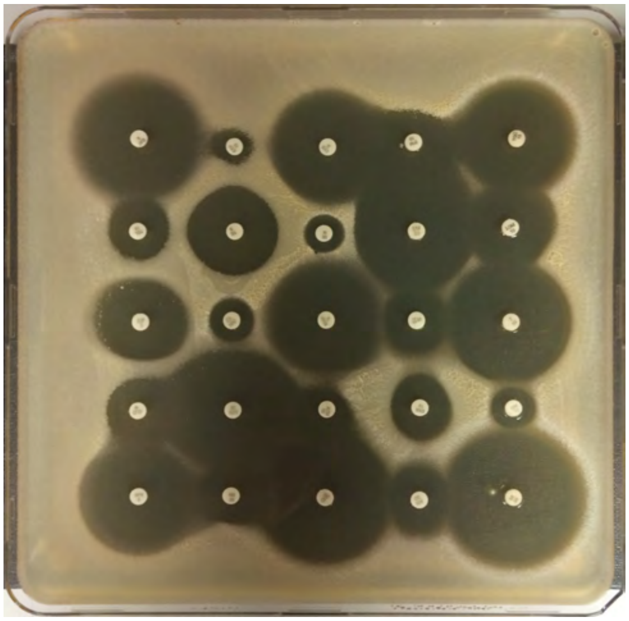

Ms∆metH

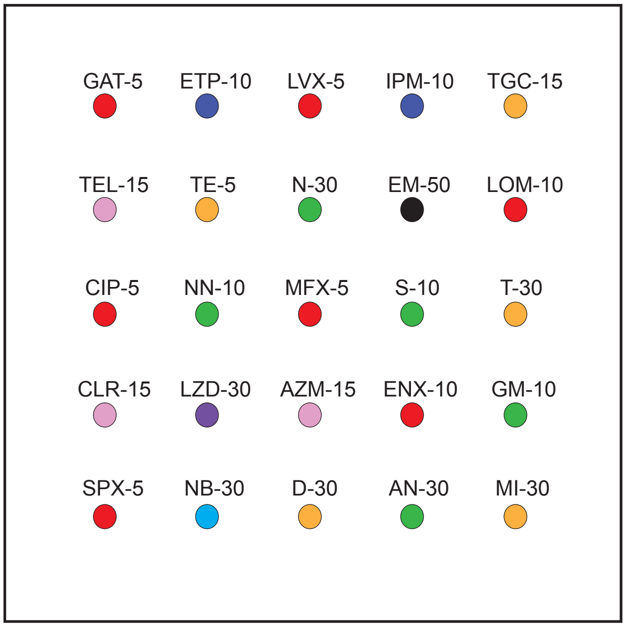

Code	Antibiotic	Class	Target	Pathway
ETP-10	Ertepenem	β-lactams	Peptidoglycan (PBPs)	Cell wall synthesis
IPM-10	Imipenem			
CLR-15	Clarithromycin	Macrolides	Ribosome	Protein synthesis
AZM-15	Azithromycin			
TEL-15	Telithromycin			
GM-10	Gentamycin	Aminoglycosides		
AN-30	Amikacin			
S-10	Streptomycin			
N-30	Neomycin			
NN-10	Tobramycin			
LZD-30	Linezolid	Oxazolidinones		
TE-5	Tetracycline	Tetracycline antibiotics		
MI-30	Minocycline			
D-30	Doxycycline			
T-30	Oxytetracycline			
TGC-15	Tygecycline			
NB-30	Novobiocin	Aminocoumarins	DNA gyrase	DNA replication
ENX-10	Enoxacin	Quinolones		
GAT-5	Gatifloxacin			
CIP-5	Ciprofloxacin			
LOM-10	Lomefloxacin			
SPX-5	Sparfloxacin			
LVX-5	Levofloxacin			
MFX-5	Moxifloxacin			
EM-50	Ethambutol	Antimycobacterial	Arabinogalactan (EmbC)	Mycobacterial cell wall synthesis
