# Supplementary material for: Methylfolate Trap Promotes Bacterial Thymineless Death by Sulfa Drugs
Source: PLoS Pathog. 2016 Oct 19;12(10):e1005949. doi: 10.1371/journal.ppat.1005949 (PMC5070874; doi:10.1371/journal.ppat.1005949)
Supplement: S8 Fig — A representative disc diffusion test shows that metH does not affect S. typhimurium resistance to non-antifolates. Cells of metH(+) (top left) and metH(-) (top right) were seeded onto the surface of LB agar. Antibiotic discs were applied at positions indicated in the bottom left panel. Bottom right panel indicates the antibiotics’ classification. (PDF) [file ppat.1005949.s008.pdf]

Figure S8

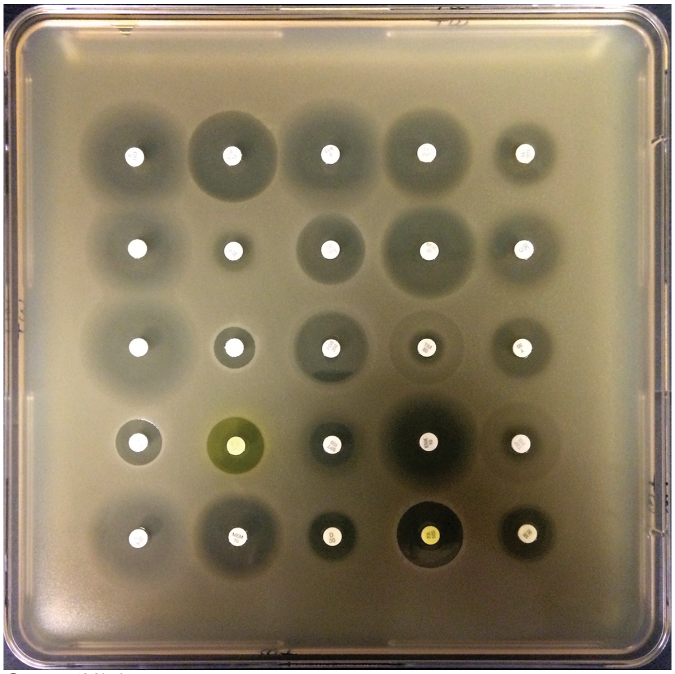

St. metH(+)

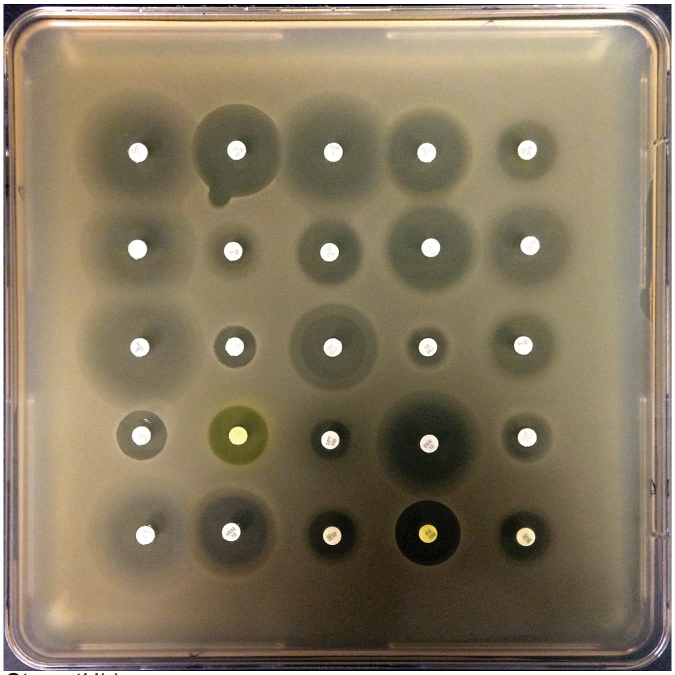

St. metH(-)

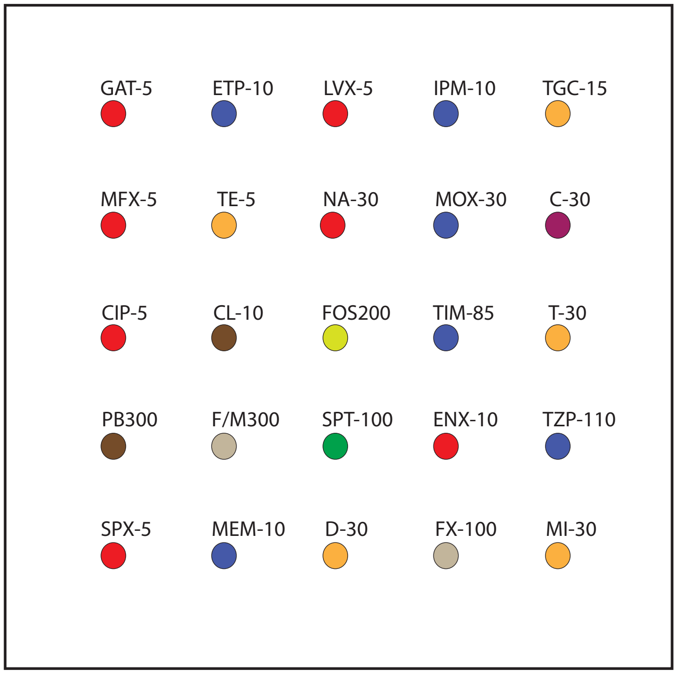

Code	Antibiotic	Class	Target	Pathway
ETP-10	Ertepenem	β-lactams	Peptidoglycan (PBPs)	Cell wall synthesis
IPM-10	Imipenem			
MEM-10	Meropenem			
MOX-30	Moxalactam			
TIM-85	Ticarcillin/Clavulanic			
TZP-110	Piperacillin/Tazobactam			
FOS200	Fosfomycin	Phosphonics	MurA	
CL-10	Colistin	Polymyxins	LPS	Membrane integrity
PB-300	Polymyxin B			
SPT-100	Spectinomycin	Aminocyclitols	Ribosome	Protein synthesis
C-30	Chloramphenicol	Amphenicols		
TE-5	Tetracycline	Tetracyclines		
MI-30	Minocycline			
D-30	Doxycycline			
T-30	Oxytetracycline			
TGC-15	Tygecycline			
NA-30	Nalidixic Acid	Quinolones	DNA gyrase	DNA replication
ENX-10	Enoxacin			
GAT-5	Gatifloxacin			
CIP-5	Ciprofloxacin			
MFX-5	Moxifloxacin			
SPX-5	Sparfloxacin			
LVX-5	Levofloxacin			
FX-100	Furazolidone	Nitrofurans	DNA	Mutagenesis
F/M300	Nitrofurantoin			
