# Supplementary material for: Methylfolate Trap Promotes Bacterial Thymineless Death by Sulfa Drugs
Source: PLoS Pathog. 2016 Oct 19;12(10):e1005949. doi: 10.1371/journal.ppat.1005949 (PMC5070874; doi:10.1371/journal.ppat.1005949)
Supplement: S1 Table — (DOC) [file ppat.1005949.s012.doc]

***Table S1.*** *Whole-genome antifolate resistance determinants in* M. smegmatis.

| **No** | | **Gene** | **Drug ^a^** | **Predicted function** | **Chem Compl** | | | |
| --- | --- | --- | --- | --- | --- | --- | --- | --- |
|  |  |  |  |  | **F** | **L** | **P** | **M** |
| 1 |  | *msmeg_0225* | S | Lipid transport (MmpL4) |  |  |  |  |
| 2 |  | *msmeg_0393* | S, T | Methionyl-tRNA formyltransferase homolog (Fmt, EC:2.1.2.9) |  |  |  |  |
| 3 |  | *msmeg_0400* | T | Peptide Synthetase (EC:5.1.1.3/6.2.1.3) |  |  |  |  |
| 4 |  | *msmeg_0410* | S | Lipid transport (MmpL) |  |  |  |  |
| 5 |  | *msmeg_0643* | S, T | Extracellular solute binding protein |  |  |  |  |
| 6 | ↓↓ | *msmeg_0677*  *msmeg_0678* | T | tRNA(Gly)  Deoxycytidine triphosphate deaminase (Dcd, EC:3.5.4.13) |  |  |  |  |
| 7 |  | *msmeg_0719* | S | Flavohemoprotein/NO dioxygenase (EC: 1.14.12.17) |  |  |  |  |
| 8 |  | *msmeg_0786* | S, T | Protein kinase G (PknG, EC:2,7,1.-) |  |  |  |  |
| 9 |  | *msmeg_1017* | T | Glutaredoxin (NrdH, EC:1.17.4.1) |  |  |  |  |
| 10 | ↓ | *msmeg_1244* | S | Hypothetical protein |  |  |  |  |
| 11 | ↓ | *msmeg_1245* | S | Phosphoadenyl-sulfate reductase (CysH, EC: 1.8.4.8) |  |  |  |  |
| 12 |  | *msmeg_1342* | S | (3R)-hydroxyacyl-ACP dehydratase (HadC, EC:4.2.1.61) |  |  |  |  |
| 13 |  | *msmeg_1859* | S | Hypothetical protein |  |  |  |  |
| 14 | ↓↓ | *msmeg_1869*  *msmeg_1868* | T | Hypothetical protein  Hypothetical protein |  |  |  |  |
| 15 |  | *msmeg_1930* | S, T | DEAD/DEAH box helicase (EC:3.6.1.-) |  |  |  |  |
| ­16 |  | *msmeg_1959* | S | Putative membrane protein |  |  |  |  |
| 17 | ↓↓ | *msmeg_2090*  *msmeg_2091* | S | Cell division protein (FtsX)  SsrA-binding protein (SmpB) |  |  |  |  |
| 18 |  | *msmeg_2163* | S | Succinic-semialdehyde dehydrogenase homolog (GabD, EC: 1.2.1.16) |  |  |  |  |
| 19 |  | *msmeg_2391* | S | Polyphosphate kinase (Ppk, EC:2.7.4.1) |  |  |  |  |
| 20 |  | *msmeg_2752* | S | Sigma factor SigB |  |  |  |  |
| 21 |  | *msmeg_3046* | T^R^ | Carbamoyl phosphate synthase (CarA, EC: 6.3.5.5) |  |  |  |  |
| 22 |  | *msmeg_3070* | S | Lipoprotein LprG |  |  |  |  |
| 23 |  | *msmeg_3078* | T | Excinuclease ABC subunit C (UvrC, EC:3.1.25.-) |  |  |  |  |
| 24 |  | *msmeg_3236* | T | ABC-type amino acid transport system, permease |  |  |  |  |
| 25 |  | *msmeg_3756* | S | 23S ribosomal RNA |  |  |  |  |
| 26 |  | *msmeg_3873* | S | Precorrin-2 C20/3B C17-methyltransferase (CobIJ, EC:1.2.1.130/1.2.1.131) |  |  |  |  |
| 27 |  | *msmeg_3890* | T | Proteasone accessory factor A (PafA, EC:6.3.2.-) |  |  |  |  |
| 28 | ↓↓↓ | *msmeg_3896*  *msmeg_3895*  *msmeg_3894* | T | Prokaryotic ubiquitin-like protein (Pup)  Proteasome beta subunit (PrcB, EC:3.4.25.1)  Proteasome alpha subunit (PrcA, EC:3.4.25.1) |  |  |  |  |
| 29 |  | *msmeg_4013* | S | 5,10-methylenetetrahydromethanopterin reductase (Hmd, EC:1.5.99.11) |  |  |  |  |
| 30 |  | *msmeg_4185* | S | Methionine synthase (MetH, EC:2.1.1.13) |  |  |  |  |
| 31 |  | *msmeg_4302* | S | Adenylate cyclase (EC:4.6.1.1) |  |  |  |  |
| 32 |  | *msmeg_5042* | S, T | ATP-dependent RNA Helicase (EC:3.6.4.13) |  |  |  |  |
| 33 |  | *msmeg_5072* | S | Sigma factor SigE |  |  |  |  |
| 34 |  | *msmeg_5129* | S | Mycothiol synthesis (MshB, EC:3.5.1.103) |  |  |  |  |
| 35 |  | *msmeg_5239* | S | Fructose 1,6-bisphosphatase II (GlpX, EC:3.1.3.11) |  |  |  |  |
| 36 |  | *msmeg_5249* | S | Serine Hydroxymethyltransferase (GlyA, EC:2.1.2.1) |  |  |  |  |
| 37 |  | *msmeg_5472* | S, T | 5-formyltetrahydrofolate cyclo-ligase (YgfA, EC:6.3.3.2) |  |  |  |  |
| 38 |  | *msmeg_5488* | S | Two-component system, response regulator (MprA) |  |  |  |  |
| 39 |  | *msmeg_5661* | S | ABC transporter |  |  |  |  |
| 40 |  | *msmeg_5696* | T | Cold shock protein (CspB) |  |  |  |  |
| 41 |  | *msmeg_5781* | S | Phosphate ABC transporter, permease protein (PstC) |  |  |  |  |
| 42 |  | *msmeg_5796* | S | Folate binding protein YgfZ (EC:2.1.2.10) |  |  |  |  |
| 43 |  | *msmeg_6154* | S, T | Adenylate cyclase (EC:4.6.1.1) |  |  |  |  |
| 44 |  | *msmeg_6398* | T | Mycolyltransferase (FbpA, EC:2.3.1.-) |  |  |  |  |
| 45 |  | *msmeg_6408* | S, T | Acyltransferase (EC:2.3.1.-) |  |  |  |  |
| 46 |  | *msmeg_6409* | S, T | Acyltransferase (EC:2.3.1.-) |  |  |  |  |
| 47 |  | *msmeg_6515* | T | Trehalose synthase (TreS, EC:5.4.99.16) |  |  |  |  |
| 48 |  | *msmeg_6562* | T | Acyl-CoA dehydrogenase (EC:1.3.99.3) |  |  |  |  |
| 49 |  | *msmeg_6828* | S | Transcriptional regulator |  |  |  |  |
| 50 |  | *msmeg_6914* | T | Trahalase-like protein (EC:3.2.1.-) |  |  |  |  |
| **^a^ S = SULFAs, T = TMP,** ↓ **= gene cluster, F = PteGlu_1_, L = 5-CHO-H_4_PteGlu_1_, P = pABA, M = 5-CH_3_-H_4_PteGlu_1_.**  **Chem Compl = chemical complementation. Shading indicates the level of growth.** | | | | | | | | |
