# Supplementary material for: Methylfolate Trap Promotes Bacterial Thymineless Death by Sulfa Drugs
Source: PLoS Pathog. 2016 Oct 19;12(10):e1005949. doi: 10.1371/journal.ppat.1005949 (PMC5070874; doi:10.1371/journal.ppat.1005949)
Supplement: S2 Table — (DOC) [file ppat.1005949.s013.doc]

**Table S2. Strains used in this study.**

| **Name** | **Relevant features** | **References** |
| --- | --- | --- |
| mc^2^155 | *Mycobacterium smegmatis* wild type |  |
| 63H1 | mc^2^155 derived, *Himar1* transposon *metH* mutant (TA^499-500^) | This study |
| 121D7 | mc^2^155 derived, *Himar1* transposon *metH* mutant (TA^2881-2882^) | This study |
| 58B10 | mc^2^155 derived, *Himar1* transposon *metH* mutant (TA^3091-3092^) | This study |
| 71B10 | mc^2^155 derived, *Himar1* transposon *cobIJ* mutant (TA^112-113^) | This study |
| 49H2 | mc^2^155 derived, *Himar1* transposon *cobIJ* mutant (TA^112-113^) | This study |
| *Ms*Δ*metH* | mc^2^155 derived, targeted *metH* deletion mutant | This study |
| *Ms*Δ*metH*/*metH* | *Ms*Δ*metH* strain expressing *M. smegmatis metH* from pVN915 | This study |
| *Ms*Δ*cobIJ* | mc^2^155 derived, targeted *cobIJ* deletion mutant | This study |
| *Ms*Δ*cobIJ*/*cobIJ* | *Ms*Δ*cobIJ* strain expressing *M. smegmatis cobIJ* from pVN958 | This study |
| *Ms*Δ*metE* | mc^2^155 derived, targeted *metE* deletion mutant | This study |
| *Ms*Δ*metE*/*metE* | *Ms*Δ*metE* expressing *M. smegmatis metE* from pVN969 | This study |
| *Ms*Δ*metE*Δ*metH* | mc^2^155 derived, targeted *metE metH* double deletion mutant | This study |
| *Ms*Δ*metE*Δ*metH*/*metH* | *Ms*Δ*metE*Δ*metH* expressing *M. smegmatis metE* from pVN969 | This study |
| H37Rv | *Mycobacterium tuberculosis* laboratory strain |  |
| *Rv*Δ*metH* | H37Rv-derived, targeted *metH* deletion mutant | This study |
| *Rv*Δ*metH*/*metH* | *Rv*Δ*metH* strain expressing *M. tuberculosis metH* from pVN867 | This study |
| *Rv*Δ*cobIJ* | H37Rv-derived, targeted *cobIJ* deletion mutant | This study |
| CDC1551 | *Mycobacterium tuberculosis* clinical isolate |  |
| CDC1551/*metH* | CDC1551 strain expressing H37Rv *metH* from pVN867 | This study |
| CDCΔ*cobIJ* | CDC1551-derived, targeted *cobIJ* deletion mutant | This study |
| CDCΔ*cobIJ*/*metH* | CDCΔ*cobIJ* strain expressing H37Rv *metH* from pVN867 | This study |
| CDCΔ*bacA* | CDC1551-derived, targeted *bacA* deletion mutant | BEI |
| CDCΔ*bacA*/*metH* | CDCΔ*bacA* strain expressing H37Rv *metH* from pVN867 | This study |
| phVN893 | TM4-derived *Mtb*Δ*metH*::hyg recombinant phage | This study |
| phVN1004 | TM4-derived *Mtb*Δ*cobIJ*::hyg recombinant phage | This study |
| BW25113 | *Escherichia coli* wild type | Keio |
| *Ec*Δ*metH* | BW25113 derived, targeted *metH* deletion mutant | Keio |
| *Ec*Δ*btuB* | BW25113 derived, targeted *btuB* deletion mutant | This study |
| *Ec*Δ*btuCED* | BW25113 derived, targeted *btuCED* deletion mutant | This study |
| *Ec*Δ*btuB btuCED* | BW25113 derived, targeted *btuB* *btuCED* mutant | This study |
| MPA01 | *Pseudomonas aeruginosa* wild type | Manoil Lab |
| PW3296 | MPA01 derived, *btuB* (PA1271) transposon mutant | Manoil Lab |
| PW5878 & PW5879 | MPA01 derived, *cobJ* (PA2903) transposon mutant | Manoil Lab |
| PW5880 & PW5881 | MPA01 derived, *cobI* (PA2904) transposon mutant | Manoil Lab |
| PW4235 & PW4236 | MPA01 derived, *metH* (PA1843) transposon mutant | Manoil Lab |
| PW5882 | MPA01 derived, *cobH* (PA2905) transposon mutant | Manoil Lab |
| TT10000 | *Salmonella typhimurium* wild type | Roth Lab |
| TT16729 | *Salmonella typhimurium metE* | Roth Lab |
| TT10842 | *Salmonella typhimurium metH* | Roth Lab |
| TT11202 | *Salmonella typhimurium metE metH* | Roth Lab |
| TT16830 | *Salmonella typhimurium metE btuB* | Roth Lab |
| TT16667 | *Salmonella typhimurium metE cobI cobJ* | Roth Lab |
| TT16831 | *Salmonella typhimurium metE cobI cobJ btuB* | Roth Lab |
| TT16848 | *Salmonella typhimurium metE cobI* | Roth Lab |
| TT16849 | *Salmonella typhimurium metE cob* deletion mutant | Roth Lab |

**References**

1. Snapper SB, Melton RE, Mustafa S, Kieser T, Jacobs WR, Jr. Isolation and characterization of efficient plasmid transformation mutants of *Mycobacterium smegmatis*. Mol Microbiol. 1990;4(11):1911-9. PubMed PMID: 2082148.

2. Cole ST, Brosch R, Parkhill J, Garnier T, Churcher C, Harris D, et al. Deciphering the biology of *Mycobacterium tuberculosis* from the complete genome sequence. Nature. 1998;393(6685):537-44. PubMed PMID: 9634230.

3. Valway SE, Sanchez MP, Shinnick TF, Orme I, Agerton T, Hoy D, et al. An outbreak involving extensive transmission of a virulent strain of *Mycobacterium tuberculosis*. N Engl J Med. 1998;338(10):633-9. Epub 1998/03/05. PubMed PMID: 9486991.

4. Lamichhane G, Zignol M, Blades NJ, Geiman DE, Dougherty A, Grosset J, et al. A postgenomic method for predicting essential genes at subsaturation levels of mutagenesis: application to *Mycobacterium tuberculosis*. Proc Natl Acad Sci U S A. 2003;100(12):7213-8. Epub 2003/05/31. doi: 10.1073/pnas.1231432100

1231432100 [pii]. PubMed PMID: 12775759; PubMed Central PMCID: PMC165855.

5. Baba T, Ara T, Hasegawa M, Takai Y, Okumura Y, Baba M, et al. Construction of *Escherichia coli* K-12 in-frame, single-gene knockout mutants: the Keio collection. Mol Syst Biol. 2006;2:2006 0008. Epub 2006/06/02. doi: msb4100050 [pii]

10.1038/msb4100050. PubMed PMID: 16738554; PubMed Central PMCID: PMC1681482.

6. Jacobs MA, Alwood A, Thaipisuttikul I, Spencer D, Haugen E, Ernst S, et al. Comprehensive transposon mutant library of *Pseudomonas aeruginosa*. Proc Natl Acad Sci U S A. 2003;100(24):14339-44. Epub 2003/11/18. doi: 10.1073/pnas.2036282100. PubMed PMID: 14617778; PubMed Central PMCID: PMC283593.

7. Sampson BA, Gotschlich EC. Elimination of the vitamin B12 uptake or synthesis pathway does not diminish the virulence of *Escherichia coli* K1 or *Salmonella typhimurium* in three model systems. Infect Immun. 1992;60(9):3518-22. Epub 1992/09/01. PubMed PMID: 1500158; PubMed Central PMCID: PMC257354.
