# Supplementary material for: Methylfolate Trap Promotes Bacterial Thymineless Death by Sulfa Drugs
Source: PLoS Pathog. 2016 Oct 19;12(10):e1005949. doi: 10.1371/journal.ppat.1005949 (PMC5070874; doi:10.1371/journal.ppat.1005949)
Supplement: S3 Table — (DOC) [file ppat.1005949.s014.doc]

**Table S3. Plasmids used in this study.**

| **Name** | **Relevant features** | **References** | |
| --- | --- | --- | --- |
| pMycoMar | *Himar1*, temperature-sensitive mycobacterial replicon | |  |
| pMV361 | *E. coli-Mycobacterium* shuttle integrative vector, Kan^R^, P_hsp60_ | |  |
| pCV125 | *E. coli-Mycobacterium* shuttle integrative vector, Kan^R^, *lacZ* | |  |
| pMS2 | *E. coli-Mycobacterium* shuttle replicative vector, Hyg^R^ | |  |
| pYUB854 | *E. coli* plasmid (Ωhyg) for constructing allelic exchange substrates | |  |
| pVN701B | *Mycobacterium* vector expressing the recombineering, ts-Ori, *sucA* | |  |
| pGH542 | *Mycobacterium* vector expressing the γδ resolvase | |  |
| pVN747 | pMS2 derivative, Hyg^R^, P_SOD_ | |  |
| pKD3 | *E. coli* vector used as template for Chl^R^ PCR amplification | |  |
| pKD13 | *E. coli* vector used as template for Kan^R^ PCR amplification | |  |
| pRsBs-ACbl-βgal-HC | pBAD derived plasmid for use as B_12_ probe, β-gal reporter | |  |
| pRsBs-ACbl-FFluc-HC | pBAD derived plasmid for use as B_12_ probe, FFluc reporter | |  |
| pRsBs-ACbl-RFP-HC | pBAD derived plasmid for use as B_12_ molecular probe, RFP reporter | |  |
| pVN867 | pCV125 derived, expressing *Mtb metH* from native promoter | | [12] |
| pVN869 | pYUB854 derived, carrying *Ms*Δ*metH*::Ωhyg AES | | [12] |
| pVN893 | pYUB854 derived, carrying *Mtb*Δ*metH*::Ωhyg AES | | [12] |
| pVN915 | pCV125 derived, expressing *Ms metH* from native promoter | | [12] |
| pVN930 | pVN747 derived, expressing *Mtb metH* from native promoter | | [12] |
| pVN957 | pYUB854 derived, carrying *Ms*Δ*cobIJ*::Ωhyg AES | | [12] |
| pVN958 | pMV361 derived, expressing *Ms cobIJ* from P_hsp60_ | | [12] |
| pVN973 | pYUB854 derived, carrying *Ms*Δ*metE*::Ωhyg AES | | [12] |
| pVN969 | pMV361 derived, expressing *Ms metE* from P_hsp60_ | | [12] |
| pVN1004 | pYUB854 derived, carrying *Mtb*Δ*cobIJ*::Ωhyg AES | | [12] |

AES, allelic exchange substrate; *Mtb*, *M. tuberculosis; Ms, M. smegmatis*

**References**

1. Rubin EJ, Akerley BJ, Novik VN, Lampe DJ, Husson RN, et al. (1999) In vivo transposition of mariner-based elements in enteric bacteria and mycobacteria. Proc Natl Acad Sci U S A 96: 1645-1650.

2. Lampe DJ, Akerley BJ, Rubin EJ, Mekalanos JJ, Robertson HM (1999) Hyperactive transposase mutants of the *Himar1* mariner transposon. Proc Natl Acad Sci U S A 96: 11428-11433.

3. Stover CK, de la Cruz VF, Fuerst TR, Burlein JE, Benson LA, et al. (1991) New use of BCG for recombinant vaccines. Nature 351: 456-460.

4. Alland D, Steyn AJ, Weisbrod T, Aldrich K, Jacobs WR, Jr. (2000) Characterization of the *Mycobacterium tuberculosis iniBAC* promoter, a promoter that responds to cell wall biosynthesis inhibition. J Bacteriol 182: 1802-1811.

5. Kaps I, Ehrt S, Seeber S, Schnappinger D, Martin C, et al. (2001) Energy transfer between fluorescent proteins using a co-expression system in *Mycobacterium smegmatis*. Gene 278: 115-124.

6. Braunstein M, Bardarov SS, Jacobs WR, Jr. (2002) Genetic methods for deciphering virulence determinants of *Mycobacterium tuberculosis*. Methods Enzymol 358: 67-99.

7. Wolff KA, Nguyen HT, Cartabuke RH, Singh A, Ogwang S, et al. (2009) Protein kinase G is required for intrinsic antibiotic resistance in mycobacteria. Antimicrob Agents Chemother 53: 3515-3519.

8. Piuri M, Hatfull GF (2006) A peptidoglycan hydrolase motif within the mycobacteriophage TM4 tape measure protein promotes efficient infection of stationary phase cells. Mol Microbiol 62: 1569-1585.

9. Datsenko KA, Wanner BL (2000) One-step inactivation of chromosomal genes in *Escherichia coli* K-12 using PCR products. Proc Natl Acad Sci U S A 97: 6640-6645.

10. Johnson JE, Lackner LL, Hale CA, de Boer PA (2004) ZipA is required for targeting of DMinC/DicB, but not DMinC/MinD, complexes to septal ring assemblies in *Escherichia coli*. J Bacteriol 186: 2418-2429.

11. Fowler CC, Brown ED, Li Y (2010) Using a riboswitch sensor to examine coenzyme B(12) metabolism and transport in *E. coli*. Chem Biol 17: 756-765.

12. This study.
