# Supplementary material for: Methylfolate Trap Promotes Bacterial Thymineless Death by Sulfa Drugs
Source: PLoS Pathog. 2016 Oct 19;12(10):e1005949. doi: 10.1371/journal.ppat.1005949 (PMC5070874; doi:10.1371/journal.ppat.1005949)
Supplement: S4 Table — (DOC) [file ppat.1005949.s015.doc]

**Table S4. Oligonucleotides used in this study.**

| **Name** | **Sequence** |
| --- | --- |
| Mar-Ext1 | gggaatcatttgaaggttggt |
| Mar-Ext2 | gtcaattcgagctcgggta |
| Mar-Int1 | tagcgacgccatctatgtgtc |
| Mar-Int2 | cttgaagggaactatgttg |
| ARB1 | ggccacgcgtcgactagtacnnnnnnnnnngatat |
| ARB6 | ggccacgcgtcgactagtacnnnnnnnnnnacgcc |
| ARB2 | ggccacgcgtcgactagtac |
| btuCED-del1 | accgtaaaatcaacgccatgacacttcagcagaacggatacctccggggatccgtcgacc |
| btuCED-del2 | cagacggcgaaaattcatcccataggcctgcgccagattgtgtaggctggagctgcttcg |
| btuCED-conf1 | gcgtggtgaccttcagaccc |
| btuCED-conf2 | atcagcattctgtgaccttcgatatc |
| btuB-del1 | tattgatgaaacctgcggcatccttcttctattgtggatgctttacaatgccatatgaatatcctccttag |
| btuB-del2 | agtcaaacaccagcacggtgggacgtggttcagaaggtgtagctgccagagtgtaggctggagctgcttcg |
| btuB-conf1 | gggaagtcatcatctcttagtatcttaga |
| btuB-conf2 | cgacgttatcgaaagcataaatgtaatgg |
| MSmetH-Del1 | actagtccttcgacgtgccgtcgagac |
| MSmetH-Del2 | aagcttgttcatgtgcactccttccgtagc |
| MSmetH-Del3 | tctagagcaagggttggacgctgcagg |
| MSmetH-Del4 | ggtaccgatgaaccaccgaccgtcgattc |
| metH-Del5L | gacgatggcgttctccagttcg |
| metH-Del6R | atgcagccagtccgcagattc |
| CobIJ-Del1 | tcatgagtgctcgactacatccgtgacg |
| CobIJ-Del2 | tctagagccgtgcttgttctcgctcattc |
| CobIJ-Del3 | aagcttagatctcgcacctaccccggctgac |
| CobIJ-Del4 | actagttcatcgcgtctgctccggttc |
| cobIJ-conf1 | tgctggtgttcgacctcgacga |
| cobIJ-conf2 | cgatctcgcagtggtagcagtg |
| MTBmetH-Del1 | actagtagcaactcgtcgcaaaacgtgcg |
| MTBmetH-Del2 | aagcttgacgtcgagcagatcggtgtcg |
| MTBmetH-Del3 | tctagaggcgttcgtcctgcaccatccg |
| MTBmetH-Del4 | tcatgacggagtcaccctggtgggtacc |
| Mtb-metH-conf1 | cccacacaggcagattggaagacg |
| Mtb-metH-conf2 | ccactgatctatttcggaccgttcg |
| Ms-metHpro1 | gaattctagatacgacgatgtcacactgccag |
| Ms-metH2 | ggtaccatatggattgttcgggtagcgctcagtc |
| Mtb-metHpro1 | tctagactggtcagcctacggcgtcca |
| Mtb-metH2 | catatgaagcttggtcagattaaacgttgaagtacttggc |
| CobIJ1-ENd | gaattccatatgagcgagaacaagcacggcac |
| CobIJ2-H | aagcttagtcaatgattgtcgacgtgcgtca |
| MetE-Del1 | TCTAGAgcagtggcatcacggtgatcgg |
| MetE-Del2 | accggtGGCCATcgccttgagacgtcgaa |
| MetE-Del3 | ctcGAGAAGCACGAGCGGATGCCTGA |
| MetE-Del4 | ACTAGTccgaactggtcgtcgatcgcag |
| MetE-conf1 | agttcgccaccagtcgcacgc |
| MetE-conf2 | cgagggtgtcgtagcccaacac |
| Ms-metE1 | GAATTCCATatggccttccgccgtaccgaag |
| Ms-metE2 | AAGCTTGGATCCtcaggcatccgctcgtgcttctc |
| MTBcobIJ-Del1 | ACTagtgctcgactacctacgcgacg |
| MTBcobIJ-Del2 | AAGCTTgagcgctcattcgcggtcgc |
| MTBcobIJ-Del3 | TCTAgattcgcaggaccgggtgttcac |
| MTBcobIJ-Del4 | GGTACctatctcacgaaagctcgggatttac |
| MTBcobIJ-conf1 | ggcatttcgtcggctgcgagc |
| MTBcobIJ-conf2 | ctcgttactaccgacgtatcatccg |
