# Supplementary material for: Methylfolate Trap Promotes Bacterial Thymineless Death by Sulfa Drugs
Source: PLoS Pathog. 2016 Oct 19;12(10):e1005949. doi: 10.1371/journal.ppat.1005949 (PMC5070874; doi:10.1371/journal.ppat.1005949)
Supplement: S1 Text — (DOC) [file ppat.1005949.s016.doc]

**Text S1. Additional methods**

Strains, plasmids, oligonucleotides and special chemicals.

Bacterial strains and plasmids used in this study are listed in Tables S2 and S3, respectively. Oligonucleotides (Table S4) were purchased from Eurofins MWG Operon (Huntsville, AL). Folate derivatives were purchased from Schirck’s Laboratories (Jona, Switzerland).

Bacterial growth conditions.

Wild type parental strain *M. smegmatis* mc^2^155 and its derived transposon mutants were grown in 7H9 (Difco) or Luria-Bertani (LB) broth supplemented with glucose and 0.5 % Tween 80. Strains were propagated on 7H10 or LB agar. Drug susceptibility was done on NE medium or otherwise stated. *M. tuberculosis* strains were grown in 7H10-OADC or Dubos-ADC media (Difco). Kanamycin (50 μg/ml) was used for maintaining transposon mutants. Hygromycin was used at 75 and 100 μg/ml for *M. smegmatis* and *E. coli*, respectively. Transformation and genetic manipulations were carried as described .

Construction of *M. smegmatis* transposon library and screen for antifolate sensitive mutants.

The temperature-sensitive vector pMycoMar was used to deliver the *Himar1* mini-transposon to *M. smegmatis* mc^2^155 by electroporation . Transformants recovered overnight at 28°C were plated on LB agar plates containing 50 μg/ml kanamycin. After 5 days of growth at 39°C, single colonies were picked and grown separately in 96-deep well plates in LB medium containing 50 μg/ml kanamycin for 2 days. Five wells at different positions in each 96-well plate were inoculated with a kanamycin resistant *M. smegmatis* mc^2^155 strain to serve as growth controls. Using a 96-prong replicator, cultures from these “master plates” were replicated onto NE solid medium containing kanamycin in the absence or presence of serial concentrations of SCP (10, 15, 20, 25 and 50 μg/ml) or TMP (1.25, 1.5, 2, 2.5 and 3 μg/ml). After 5 days of growth at 37^o^C, strains that grew on NE-kanamycin plates but failed to grow on plates supplemented with antifolates were selected. These mutants were subjected to two additional rounds (each from a different colony) of replication to confirm drug susceptibility patterns.

## Arbitrary PCR identification of antifolate resistance determinants*.*

## Mapping of transposon insertion sites in the mutants using arbitrary PCR was carried out as previously described . Briefly, DNA sequences covering the chromosome-transposon transitions were first PCR-amplified using a random-sequenced primer (ARB1 or ARB6, Table S4) and a *Himar1-*specific primer (Mar-Ext1 or Mar-Ext2). Next, products of the first PCR were used as templates for a second round PCR, which used primers that recognize sequences of the first round primers (ARB2 and Mar-Int1 or Mar-Int2), to enrich the DNA of the transitional sequences. PCR products were purified using Qiagen PCR purification Kit, followed by sequencing (ACGT, Inc.). Sequences were Blast searched against the *M. smegmatis* genome sequence deposited in the National Center for Biotechnology Information. Transposon insertions were confirmed by PCR using primers that recognize chromosomal sequences outside the insertion sites, followed by sequencing, repeated Blast searches and sequence alignments.

Chemical complementation profiling of antifolate-sensitive mutants.

Antifolate sensitive mutants selected from the screen were deposited in 96-well plates. The replication was repeated using antifolates-containing NE plates in the absence or presence of folic acid (PteGlu_1_), folinic acid (5-CHO-H_4_PteGlu_1_), 5-methyltetrahydrofolate (5-CH_3_-H_4_PteGlu_1_), or pABA at 0.1 or 0.3 mM final concentrations. Chemical restoration of antifolate resistance was recorded on day 7 after replication (Fig. 2).

Extraction and analysis of folate derivatives.

Folate extraction was performed under subdued light to minimize degradation using established procedures . Strains growing in LB medium were treated with 285 µg/ml SCP (*M. smegmatis*) or 2.5 mg/ml SMZ (Gram-negative bacteria) for 15-30 min. When needed, vitamin B_12_ was used at 11 µM final concentration. Cells were harvested by centrifugation and resuspended in chilled extraction buffer (80 % methanol, 0.1 % ascorbic acid, and 20 mM ammonium acetate, pH 6.2) containing [^13^C]-5-CHO-H_4_PteGlu_1_ as an internal standard. Cell disruption was done by sonication (9x20 sec) with 1-min intervals on ice. Unbroken cells and debris were removed by centrifugation and filtration with a filter cutoff of 0.45 µm. The extracts were dried in N_2_ gas and resuspended in a solvent mixture consisting of 95 % solution A (0.1 % of formic acid in water) and 5 % solution B (0.1 % of formic acid in acetonitrile). Precipitated protein was removed by centrifugation, and the supernatant was analyzed by high performance liquid chromatography and tandem mass spectrometry (HPLC-MS/MS) as previously described . Run conditions for HPLC-MS/MS, preparation of calibrators, and data analyses were carried out as described . The procedures used in the current work are similar to previously published work in terms of recovery efficiency, yield and folate stability . All detectable folate species with varied number of glutamate residue (n=1-7) were recorded. Values of 5-CH_3_-H_4_PteGlu_n_, R-H_4_PteGlu_n_ (with R#CH_3_), and “total folate” represent the sum of all detectable species of methylated folate, non-methylated folate, and both classes, respectively.

Extraction and analysis of homocysteine thiolactone.

Overnight cultures, grown from single colonies at 37°C and 245 r.p.m., were centrifuged at 4,000 r.p.m. at room temperature for 30 min. Cell pellets were washed with fresh M9 media and diluted to OD_600nm_ of 7. 20 ml-volumes of cell suspensions were treated with 2.5 mg/ml SMZ and cultures continued to grow at 37°C with shaking at 50 r.p.m. At each designated time point, 1-ml cell aliquots were collected and spun at 14,000 r.p.m. and 4°C for 40 min. Supernatants were transferred to a clean tube and analyzed by HPLC with fluorescence detection as described .

Metabolite extraction and targeted metabolomics.

Overnight cultures, grown from single colonies at 37°C and 245 r.p.m., were used as seeds to inoculate fresh LB medium [final OD of 0.1]. Cultures were then grown [37°C, 245 r.p.m.] until OD reached 1. Samples (15 ml) from each strain were collected (t=0) before SMZ was added to final concentration of 2.5 mg/ml. At specified time points following SMZ addition, 15-ml samples from each strain were collected and OD_600nm_ measured.

Samples were centrifuged at 4,000 r.p.m. and 4°C for 30 min and pellets washed with PBS. Cells were resuspended in 1 ml PBS, transferred to clean Eppendorf tubes and spun at 14,000 r.p.m. and 4°C for 30 min. To each cell pellet, 300 μl 60 % methanol was added, and samples homogenized by sonication [Model Q700 QSonica, amplitude set at 95] at 4°C for a duration of 30 min, 30 sec pulse-on times followed by 55 sec chill. The extracts were centrifuged at 14,800 r.p.m. and 4°C for 10 min. Aliquots of 100 μl supernatant were placed in a sample vial spiked with 3 μl of 10 μg/ml DL-p-Chlorophenenylalanine before LC/MS-MS injection.

Samples were analyzed with the 5500 QTRAP LC/MS-MS system (Sciex, Framingham, MA) in the Metabolomics Lab at the Roy J. Carver Biotechnology Center, University of Illinois at Urbana-Champaign. Software Analyst 1.6.2 was used for data acquisition and analysis. The 1200 series HPLC system (Agilent Technologies, Santa Clara, CA) equipped with a degasser, an autosampler, and a binary pump.  Two methods were performed for LC/MS-MS analysis with DL-p-Chlorophenenylalanine as the internal standard.  In method 1, the LC separation was performed on a Thermo Hypercarb column (4.6x100 mm, 5 μm) with mobile phase A (0.1 % formic acid in water) and mobile phase B (0.1 % formic acid in acetontrile) with a flow rate of 0.4 ml/min. The linear gradient was as follows: 0-2 min, 100 % A; 8-13 min, 5 % A; 13.5-20 min, 100 % A. The autosampler was set at 10°C and injection volume was 5μl. Mass spectra were acquired under positive electrospray ionization (ESI) with the ion spray voltage at +5500V. The source temperature was 450^o^C. The curtain gas, ion source gas 1, and ion source gas 2 were 35, 65, and 55 p.s.i., respectively.  Multiple reaction monitoring (MRM) was used for quantitation. In method 2, the LC separation was performed on a Phenomenex Luna 5u C18(2) column (4.6x250 mm, 5 μm) with mobile phase A (10 mM ammonia formate) and mobile phase B (methanol) with a flow rate of 0.55 ml/min. The linear gradient was as follows: 0-1 min, 95 % A; 6-14 min, 1 % A; 14.5-22 min, 95 % A. The autosampler was set at 10^o^C and injection volume was 5 μl.  Mass spectra were acquired under both positive (ion spray voltage was +5500V) and negative (ion spray voltage was -4500V) ESI. The source temperature was 500^o^C. The curtain gas, ion source gas 1, and ion source gas 2 were 35, 65, and 55 p.s.i., respectively.  Multiple reaction monitoring (MRM) was used for quantitation.

Measurement of cellular synthesis of macromolecules.

Overnight cultures, grown from single colonies at 37°C and 245 r.p.m., were used as seeds to inoculate fresh LB medium [final OD of 0.5]. Cultures were then grown [37°C, 245 r.p.m.] until OD reached 1. Cultures were treated with SMZ at a final concentration of 2.5 mg/ml (t=0) and returned to 37°C with shaking at 150-175 r.p.m. At designated time points, triplicate 1.4-ml samples from each strain were transferred into separate wells of a 6-well plate. To each well, 14 μCi [^3^H]-uracil/200 μM cold uracil or 11 μCi [^35^S]-methionine/160 μM cold methionine or 14 μCi [^3^H]-thymidine was added. Following 20 min of incubation at 37°C with gentle shaking, 200 μl 1 M NaOH was added to each well, mixed thoroughly, and incubated at 50°C for 30 min. Thereafter, 800 μl 100 % cold TCA was added to each well, mixed thoroughly, and filtered onto Whatman® glass microfiber filters (binder free, Grade GF/C). Filters were washed several times with cold 5 % TCA and air-dried. Each filter was fully submerged in 5 ml Cytoscint™-Esliquid Scintillation Cocktail and read in a LS6500 Scintillation System using a count window of 0-500 for [^3^H]-uracil and [^3^H]-thymidine, and 500-1000 for [^35^S]-methionine.

Measurement of SULFA uptake.

Overnight cultures, grown from single colonies at 37°C and 245 r.p.m., were used as seeds to inoculate fresh LB medium [final OD of 0.5]. Cultures were then grown [37°C, 245 r.p.m.] until OD reached 1 and 7.2 μCi [^3^H]-SMZ was added to 7 ml aliquots of each sample (t=0), in triplicate, followed by incubation at 37°C with shaking. At each designated time point, 1 ml of culture from each sample was collected and immediately filtered onto Whatman® glass microfiber filters (binder free, Grade GF/C). Filters were washed several times with cold 5 % TCA and air-dried. Each filter was fully submerged in 5 ml Cytoscint™-Esliquid Scintillation Cocktail and read in a LS6500 Scintillation System using a count window of 0-500 for [^3^H].

Targeted gene deletion.

The entire open reading frame of *M. smegmatis* *metH* (*msmeg_4185*) was deleted using the recombineering method as previously described . The 477-bp DNA region upstream of *msmeg_4185* was PCR-amplified using primers MSmetH-Del1 and MSmetH-Del2. Similarly, the 541-bp downstream region was amplified using primers MSmetH-Del3 and MSmetH-Del4. These DNA-arms were cloned orientationally into pYUB854 , flanking the built-in hygromycin cassette to create pVN869. The *Ms*Δ*metH*::Ωhyg linear allelic exchange substrate was removed from pVN869 by *Spe*I/*Kpn*I digestion, and used to transform *M. smegmatis* mc^2^155 cells that had been induced to express the recombineering system from pVN701B . Successful allelic recombination was confirmed by PCR amplification using primers metH-Del5L and metH-Del6R that anneal to chromosomal sequences extending the homologous sequences of the allelic exchange substrate.

The *cobIJ* (*msmeg_3873*) gene was similarly deleted from the chromosome of *M. smegmatis*. Briefly, its 656-bp upstream DNA was PCR-amplified using primers CobIJ-Del1 and CobIJ-Del2 while the 757-bp downstream DNA was amplified using primers CobIJ-Del3 and CobIJ-Del4. These DNA-arms were cloned orientationally into pYUB854 , flanking the built-in hygromycin cassette to create pVN957. The *Ms*Δ*cobIJ*::Ωhyg linear allelic exchange substrate was removed from pVN957 by *Bsp*HI/*Spe*I digestion, and used to transform *M. smegmatis* mc^2^155 cells induced to express the recombineering system . Successful allelic recombination was confirmed by PCR amplification using primers cobIJ-conf1 and cobIJ-conf2 that anneal to chromosomal sequences extending the homologous sequences of the allelic exchange substrate.

The *metE* (*msmeg_6638*) gene was similarly deleted from the chromosome of *M. smegmatis*. Briefly, its 851-bp upstream DNA was PCR-amplified using primers MetE-Del1 and MetE-Del2 while the 803-bp downstream DNA was amplified using primers MetE-Del3 and MetE-Del4. These DNA-arms were cloned orientationally into pYUB854 , flanking the built-in hygromycin cassette to create pVN973. The *Ms*Δ*metE*::Ωhyg linear allelic exchange substrate was removed from pVN973 by *Xba*I/*Spe*I digestion, and used to transform *M. smegmatis* mc^2^155 or *Ms*Δ*metH* cells induced to express the recombineering system . Transformants were selected on Luria Broth agar supplemented with hygromycin, kanamycin and methionine. Successful allelic recombination was confirmed by PCR amplification using primers MetE-conf1 and MetE-conf2, followed by *Bam*HI digestion of the PCR products.

The temperature sensitive plasmid pVN701B was later removed from *Ms*Δ*metH*, *Ms*Δ*cobIJ*, *Ms*Δ*metE,* and the *Ms*Δ*metE*Δ*metH* double mutant as previously described . Furthermore, the hygromycin cassette in these mutants were excised using plasmid pGH542 that expressed the γδ resolvase .

Specialized transduction was used to delete the entire gene encoding the putative MetH protein (*rv2124c*) in *M. tuberculosis*, as previously described . Briefly, the 565-bp 3’-downstream sequence of *rv2124c* (Right-Flank) was PCR amplified using primers MTBmetH-Del3 and MTBmetH-Del4. PCR products were cloned to pGEM-T Easy and sequence confirmed by sequencing. The cloned DNA sequence was then subcloned to pYUB854 at *Xba*I and *Kpn*I sites. Thereafter, the 629-bp 5’-upstream DNA region of *rv2124c* was similarly PCR-cloned using primers MTBmetH-Del1 and MTBmetH-Del2, followed by subcloning to the pYUB854:Right-Flank plasmid at *Spe*I and *Hind*III, to create pVN893 . The temperature-sensitive mycobacteriophage phVN893 was generated by cloning *Pac*I-digested pVN893 into the TM4-derived temperature-sensitive Phae87 genome . Transduction of *M. tuberculosis* H37Rv was carried out as described previously , and *Rv*Δ*metH* mutants were selected on 7H10-OADC plates supplemented with 75 µg/ml hygromycin at 37^o^C. The replacement of *rv2124c* by the hygromycin cassette in *Rv*Δ*metH* was confirmed by PCR using primers Mtb-metH-conf1 and Mtb-metH-conf2, which annealed to chromosomal sequences outside of the allelic exchange substrate, followed by sequencing.

Similarly, specialized transduction was used to delete the entire gene (*rv2066*) encoding the CobIJ protein in *M. tuberculosis*, as previously described . Briefly, the 651-bp 3’-downstream sequence of *rv2066* (Right-Flank) was PCR amplified using primers MTBcobIJ-Del3 and MTBcobIJ-Del4 (Table S4). PCR products were cloned to pGEM-T Easy and sequence confirmed by sequencing. The cloned DNA fragment was then subcloned to pYUB854 (Table S3) at *Xba*I and *Kpn*I sites followed by sequencing to confirm correct orientation. Thereafter, the 643-bp 5’-upstream DNA region of *rv2066* was similarly PCR-cloned using primers MTBcobIJ-Del1 and MTBcobIJ-Del2 (Table S4), followed by subcloning to the pYUB854:Right-Flank plasmid at *Spe*I and *Hind*III sites, to create pVN1004 (Table S3) . Orientation of the cloned DNA fragments was confirmed by sequencing using primers annealing to hygromycin cassette in pYUB854. The temperature-sensitive mycobacteriophage phVN1004 (Table S2) was generated by cloning pVN1004 into the unique *Pac*I site of the TM4-derived temperature-sensitive phAE87 genome as previously reported . Briefly, *Pac*I-digested pVN1004 was ligated to the *Pac*I-digested concatemerized phAE87 genomic DNA and *in vitro* packaged to the λ phage heads using λ packaging extracts (GIGAPack® III GOLD kit, Stratagene). After transduction, *E. coli* NM554 was plated on hygromycin-containing LB agar plates. Phasmid DNA prepared from a pool of hygromycin resistant transductants was electroporated into *M. smegmatis* mc^2^155. Plaques that grew at permissive temperature (28^o^ C) were purified, checked for temperature sensitivity, and then used to transduce *M. tuberculosis* strains (H37Rv, CDC1551, and CDC1551/*metH*) as previously described . *Mtb*Δ*cobIJ* transductants were selected on 7H10-OADC or NE agar plates supplemented with 75 µg/ml hygromycin at 37^o^C. The replacement of *rv2066* by the hygromycin cassette in *Mtb*Δ*cobIJ* strains was confirmed by PCR using primers MTBcobIJ-conf1 and MTBcobIJ-conf2 (Table S4), which annealed to chromosomal sequences outside of the allelic exchange substrate, followed by digestions using *Not*I or *Sph*I, and sequencing.

Gene deletion in *E. coli* was done using a one-step phage λ-Red-based recombineering method . Deletions were first created in *E. coli* TB10 (MG1655, *nadA*::Tn*10* λ*c*I857 Δ(*cro-bioA*)) expressing the λ-Red system, followed by P1 phage-mediated transduction to move the mutated loci to BW25113 or its derived mutants. Primers carrying extensions, which are homologous to the flanking DNA sequences of *btuCED* (btuCED-del1 and btuCED-del2) or *btuB* (btuB-del1 and btuB-del2) (Table S4), were used to amplify the kanamycin or chloramphenicol resistance cassettes in pKD13 or pKD3 vectors, respectively. The PCR products were purified and directly transformed into TB10 cells that had been heat-induced for expression of λ-Red. Transformants were selected on LB agar plates supplemented with appropriate selective antibiotics. Homologous recombination was verified by PCR using primers annealing to chromosomal regions outside the homologous sequences. P1 phage-mediated transduction was done as previously described .

*In trans* expression.

For *in trans* expression of *M. smegmatis metH* in mycobacteria, the 4,267 bp DNA fragment including the entire open reading frame of *metH* (*msmeg_4185*, 3741 bp) and its upstream DNA region (P_METH-MS_, 473 bp) was PCR amplified from *M. smegmatis* genomic DNA using primers Ms-metHpro1 and Ms-metH2. PCR products were ligated to pGEM-T Easy vector (Promega, Madison, WI) and the nucleotide sequence was verified by sequencing. DNA fragments were then excised with *Xba*I and *Nde*I and cloned into integrative vector pCV125 (Table S3) cut with the same enzymes to create pVN915. Similarly, the 3,829 bp DNA fragment including *M. tuberculosis metH* gene (*rv2124c*) and its 225 bp upstream region was PCR cloned from *M. tuberculosis* genomic DNA using primers Mtb-metHpro1 and Mtb-metH2. The DNA fragments were then subcloned to the *Xba*I and *Nde*I sites of pCV125 and pVN747 to create pVN867 and pVN930, respectively. Plasmid pVN915, pVN867, or pVN930 (Table S3) was transformed into the *metH* mutants (*Ms*Δ*metH*, *Rv*Δ*metH*, and CDC1551) by electroporation. Transformants were selected by appropriate selective antibiotics.

For *in trans* overexpression of *M. smegmatis cobIJ* in mycobacteria, the gene (*msmeg_3873*, 1512 bp) was PCR cloned from *M. smegmatis* genomic DNA using primers CobIJ1-ENd and CobIJ2-H. The DNA fragments were then subcloned to pMV361[*Eco*RI/*Hind*III] to create pVN958. Expression of *cobIJ* from pVN958 was therefore coupled to the built-in promoters P_hsp60_. Plasmid pVN958 was transformed to mycobacterial cells and transformants selected for kanamycin resistance.

For overexpression of the B_12_-independent methionine synthase (MetE) in mycobacteria, the encoding gene (*msmeg_6638*, 2337 bp) was PCR cloned from *M. smegmatis* genomic DNA using primers Ms-metE1 and Ms-metE2. The DNA fragments were then subcloned to pMV361[*Eco*RI/*Hind*III] to create pVN969. Expression of *metE* from pVN969 was therefore coupled to the built-in strong promoters P_hsp60_. Plasmid pVN969 was transformed to mycobacterial cells and transformants selected for kanamycin resistance.

Chemical complementation for disc diffusion tests.

Cultures of *M. smegmatis* strains were grown until mid-log phase was reached, and normalized to OD_600nm_ of 1. Aliquots (50 µl) of the OD1-cultures were casted in soft agar (0.5 %) and placed on top of NE medium plates supplemented with or without the control antifolate antidote, pABA, or a folate derivative (unless otherwise stated, supplements were used at 0.3 mM final concentrations). Paper discs embedded with 0.5 or 1 mg SCP (BD Diagnostic Systems) were placed at the center of the plates. Growth inhibition was visualized as the inhibition zones surrounding the antibiotic disc after 5 days of incubation at 37^o^C. Successful complementation was regarded as the disappearance of the inhibition zones.

Antibiotic susceptibility tests.

Determination of minimal inhibitory concentration (MIC) in Gram-negative bacteria using a Steers replicator was performed following the procedures described by the Clinical and Laboratory Standards Institute (CLSI). Antibiotic stocks were made fresh on the day of use. SMZ was dissolved in water/NaOH or DMSO, while TMP was prepared in 70 % ethanol. SMZ and TMP were mixed in a ratio of 1:19 when they were used in combination. Morphologically similar colonies were emulsified in 0.85 % saline. Turbidity was adjusted until an OD_600_ reached 0.1-0.15 (1-1.5×10^8^ c.f.u./ml) and underwent a 10 fold dilution to obtain a final inoculum of 1-1.5×10^7^ c.f.u./ml. To avoid changes in cell density, the bacterial suspensions were used within 15 min of turbidity adjustment. Next, a Steers Replicator (3 mm inoculating pins) was used to apply the bacterial suspensions onto plates with inocula of 10^4^-10^5^ c.f.u./spot. The inoculated plates were incubated at 37°C overnight and MICs were determined the next day as the concentration at which there was no visible growth or an 80 % reduction in growth when compared to growth under control conditions.

For mycobacteria, MIC was determined using MTT method as previously described . Strains were grown in 7H9-glucose (*M. smegmatis*) or 7H9-OADC (*M. tuberculosis*) until late-log phase, then normalized to OD_600_ of 1. The cell suspension was then diluted 10,000 fold in Dubos or 7H9-S medium (7H9 plus 0.1 % trypsin digest of casein, 0.5 % glycerol, and OADC). When indicated, vitamin B_12_ or/and methionine were added at 0.3 and 1 mM, respectively. SMZ was prepared in serial dilutions in same media and placed in wells of 96-well plates. Wells were inoculated with 100 μl of bacterial suspension and the plates were incubated at 37°C for 3 days (*M. smegmatis*) or 7 days (*M. tuberculosis*). MTT solution prepared in PBS buffer, pH 6.8, was added to each well, and plates were incubated at 37°C for 24 h. The reaction was stopped by adding SDS-DMF (N,N-dimethylformamide) solution to each well, followed by further incubation at 37°C for additional 24 h. The MIC was recorded as the lowest concentration of SMZ that prevented the growth-mediated conversion of MTT (yellow) to formazan (violet).

Serial dilution-spotting assays.

For Gram-negative bacteria, single colonies were used to inoculate LB broth, followed by incubation at 37°C with shaking at 250 r.p.m. until OD_600_ reached 1. Cultures underwent a series of 10-fold serial dilutions in LB. 5 µl-aliquots of each dilution were spotted onto LB agar plates containing SMZ (125 or 150 μg/ml), SCP (50 μg/ml), TMP (0.25 μg/ml), or without antibiotic. Antibiotic stocks were prepared fresh the day of use. Exogenous vitamin B_12_ was used at 2 nM final concentration. Plates were incubated overnight at 37°C and read the following day.

For *M. smegmatis*, cultures were grown in LB medium containing 0.5% Tween 80 at 37°C and 250 r.p.m. until OD_600_ reached 1. Cultures underwent 10-fold serial dilutions in the same medium. 5µl-aliquotes of each dilution were spotted onto medium plates with or without a SULFA drug. SCP was used at 5 and 10.5 μg/ml for 7H10 and NE, respectively. Exogenous folate derivatives and B_12_ were used at 0.1, 0.3 or 1 mM final concentration. Plates were incubated 4-5 days at 37°C.

siRNA transfection*.*

Cells were seeded in 6-well plates and transfected with 30 nM scrambled siRNA or CblC (MMACHC) specific siRNA (Origene) using Lipofectamine RNAiMAX (Invitrogen). After 48 h, cells were harvested for *Salmonella* infection and immunodetection using a CblC monoclonal antibody (NeuroMab).

Bacterial infection and intracellular survival assays.

*Salmonella* infection and survival assays were carried out as previously described with modifications. Briefly, 5x10^5^ macrophages (U937 and THP-1 were gifts from Dr. Clifford Harding - Case Western Reserve University; J774.A1 was a gift from Dr. Jean Pieters - University of Basel) in RPMI-1640 medium supplemented with 10 % fetal bovine serum and 2 mM L-glutamine were allowed to adhere for 24 h in duplicate 24-well plates. When it is required, 50 ng/ml PMA (Sigma) was added to the medium. Exponentially growing *Salmonella* cells were added at ratio of 50 bacteria per macrophage, and plates were centrifuged at 1,000 r.p.m. for 10 min at room temperature. Phagocytosis was allowed for 60 min at 37°C. Thereafter, extracellular bacteria were removed by three washes with PBS, followed by a 60 min treatment with gentamycin (12 µg/ml). Infected macrophages were then treated without or with 1 mg/ml SMZ for 18 h. Macrophages were lysed with 1 % Triton X-100, and cell lysates plated on LB agar. Colony forming units (c.f.u.) were counted after overnight incubation at 37^o^C. All experiments were performed in triplicates.

*M. tuberculosis* infection and survival assays were carried out as previously described with modifications. Briefly, 5x10^5^ J774.A1 macrophages were grown in DMEM medium supplemented with 10% fetal bovine serum for 24 h at 37°C, 5% CO_2_ in 24-well plates. Growing *M. tuberculosis* cultures were used for infection at ratio of 10 bacteria per macrophage for 3 h at 37°C and 5% CO_2_. Thereafter, extracellular bacteria were removed by three washes with PBS, followed by a 60 min treatment with amikacin (200 µg/ml). Infected macrophages were then treated without or with 40 µg/ml SMZ for 72 h. Macrophages were lysed with 0.05% SDS, and cell lysates plated on 7H10-OADC medium. Colony forming units (c.f.u.) were counted after 4 weeks of incubation at 37^o^C. All experiments were performed in triplicates.

References
